# Differential Effects of Oligosaccharides, Antioxidants, Amino Acids and PUFAs on Heat/Hypoxia-Induced Epithelial Injury in a Caco-2/HT-29 Co-Culture Model

**DOI:** 10.3390/ijms24021111

**Published:** 2023-01-06

**Authors:** Puqiao Lian, Paul A. J. Henricks, Harry J. Wichers, Gert Folkerts, Saskia Braber

**Affiliations:** 1 Division of Pharmacology, Utrecht Institute for Pharmaceutical Sciences, Faculty of Science, Utrecht University, 3584 CG Utrecht, The Netherlands; 2 Food & Biobased Research, Wageningen University & Research, 6700 AA Wageningen, The Netherlands

**Keywords:** intestinal epithelial cells, hypoxia, heat stress, epithelial integrity, nutritional components, tight junction

## Abstract

(1) Exposure of intestinal epithelial cells to heat and hypoxia causes a (heat) stress response, resulting in the breakdown of epithelial integrity. There are indications that several categories of nutritional components have beneficial effects on maintaining the intestinal epithelial integrity under stress conditions. This study evaluated the effect of nine nutritional components, including non-digestible oligosaccharides (galacto-oligosaccharides (GOS), fructo-oligosaccharides (FOS), chitosan oligosaccharides (COS)), antioxidants (α-lipoic acid (ALA), resveratrol (RES)), amino acids (l-glutamine (Glu), l-arginine (Arg)) and polyunsaturated fatty acids (PUFAs) (docosahexaenoic acid (DHA) and eicosapentaenoic acid (EPA)), on heat/hypoxia-induced epithelial injury. (2) Two human colonic cell lines, Caco-2 and HT-29, were co-cultured and pre-treated with the nutritional components for 48 h. After pre-treatment, the cells were exposed to heat/hypoxia (42 °C, 5% O_2_) for 2 h. Epithelial integrity was evaluated by measuring trans-epithelial electrical resistance (TEER), paracellular Lucifer Yellow (LY) permeability, and tight junction (TJ) protein expression. Heat stress and oxidative stress levels were evaluated by determining heat-shock protein-70 (HSP-70) expression and the concentration of the lipid peroxidation product malondialdehyde (MDA). (3) GOS, FOS, COS, ALA, RES, Arg, and EPA presented protective effects on epithelial damage in heat/hypoxia-exposed Caco-2/HT-29 cells by preventing the decrease in TEER, the increase in LY permeability, and/or decrease in TJ proteins zonula occludens-1 (ZO-1) and claudin-3 expression. COS, RES, and EPA demonstrated anti-oxidative stress effects by suppressing the heat/hypoxia-induced MDA production, while Arg further elevated the heat/hypoxia-induced increase in HSP-70 expression. (4) This study indicates that various nutritional components have the potential to counteract heat/hypoxia-induced intestinal injury and might be interesting candidates for future in vivo studies and clinical trials in gastrointestinal disorders related to heat stress and hypoxia.

## 1. Introduction

The homeostasis of the human gastrointestinal (GI) tract is affected by multiple factors, including changes in diet and internal environment, and exposure to stressors, such as infectious agents and toxins. Strenuous exercise is an important stressor that predisposes athletes and other persons to different intestinal disorders [1,2]. During strenuous exercise such as marathon running and cycling, blood redistribution from the intestine to peripheral limbs combined with increased body temperature may lead to local hypoxic conditions and potential heat stress in the GI tract, which results in alterations in the integrity of the intestinal epithelial barrier [3].

Junctional complexes between adjacent intestinal epithelial cells form the critical structures responsible for the integrity of the intestinal barrier. This barrier is composed of a series of tight junction (TJ), adherens junction (AJ), and desmosome proteins that regulate paracellular transport and stability and tightness of the epithelium [4]. Our previous study proved that 2-h exposure to heat (40 or 42 °C) and hypoxia (5% of oxygen) significantly decreased TJ protein expression and disturbed the cellular TJ distribution, while expression of the AJ protein E-cadherin was enhanced in a co-culture model using two human colonic epithelial cell lines, Caco-2 and HT-29 [5]. Furthermore, trans-epithelial electrical resistance (TEER) was decreased, and the epithelial permeability was increased after hypoxia and heat treatment [5]. A dysfunctional or leaky intestinal epithelial tight junction barrier allows augmented permeation of luminal antigens, endotoxins, and bacteria into the local tissues and blood circulation, possibly leading to severe local and systemic inflammatory conditions [6]. Any intervention, which prevents abnormal expression of TJ/AJ proteins or restores the epithelial integrity, may contribute to alleviate heat stress (HS)-induced intestinal injury and associated disorders.

A broad range of nutritional supplements, such as non-digestible oligosaccharides, polyunsaturated fatty acids (PUFAs), antioxidants, and amino acids, may be effective in the prevention and treatment of HS-induced intestinal disorders, not only by restoring the expression of TJ/AJ proteins, but also by modulating immune responses and stress resilience pathways [3]. Our group demonstrated that galacto-oligosaccharides (GOS) and α-lipoic acid (ALA) modulate heat shock protein (HSP)-70 expression, the key regulator of the HS response, in HS-exposed Caco-2 cells and chickens [7,8,9].

Although the association between exercise-induced heat and enhanced intestinal permeability is clear [10], the exact mechanism behind the pathology of hypoxia- and heat-induced epithelial breakdown still needs to be elucidated. The TJ protein claudin-1 can act as a target protein for hypoxia-inducible factors (HIFs) suggesting a direct relationship between hypoxia and intestinal barrier damage [11]. Additionally, hypoxia and heat can lead to increased ROS activity and thus cause peroxidative damage to epithelial cells and is one of the hypotheses proposed so far [11]. A suitable intervention strategy for this intestinal barrier injury is of great importance. In the present study, we used the combination of heat stress and hypoxia in an in vitro set up using a well-characterized Caco-2/HT-29 co-culture model (epithelial cells forming a tight intestinal epithelial barrier combined with mucus-producing cells) mimicking the human intestinal epithelial layer [12,13]. These monolayers were pre-treated with different nutritional components, including GOS, fructo-oligosaccharides (FOS), chitosan oligosaccharides (COS), ALA, resveratrol (RES), l-glutamine (Glu), l-arginine (Arg), docosahexaenoic acid (DHA), and eicosapentaenoic acid (EPA), prior to two hours of heat (42 °C) and hypoxia (5% of oxygen) treatment, followed by measuring markers of intestinal integrity, including TEER, Lucifer Yellow (LY) permeability, and the expression of TJ proteins. In addition, heat stress and oxidative stress was measured via determination of HSP-70 and HIF-1α expression levels and products of lipid peroxidation.

The goal of this study is to explore the possible impact of nutritional components on altered intestinal barrier function exerted by heat and hypoxia exposure. The outcome of this study might bring us one step closer to the goal of finding suitable dietary components for people with gastrointestinal problems that are related to heat stress and hypoxia.

## 2. Results

### 2.1. Hypoxia and Heat Exposure in Combination with the Nutritional Components Do Not Affect the Cell Viability of Caco-2/HT-29 Cell Monolayers

Cytotoxicity of all nutritional components on the Caco-2/HT-29 monolayers was evaluated in a concentration- and time-dependent manner by using the MTT assay. For each nutritional component, four biologically relevant concentrations and three time points (24, 48, and 72 h) were used (Supplementary Figure S1). These results demonstrated that after 48 h of incubation, none of the selected concentrations of the nutritional components used in the following study caused cytotoxicity in the Caco-2/HT-29 monolayers (Supplementary Figure S1).

**Figure 1:**
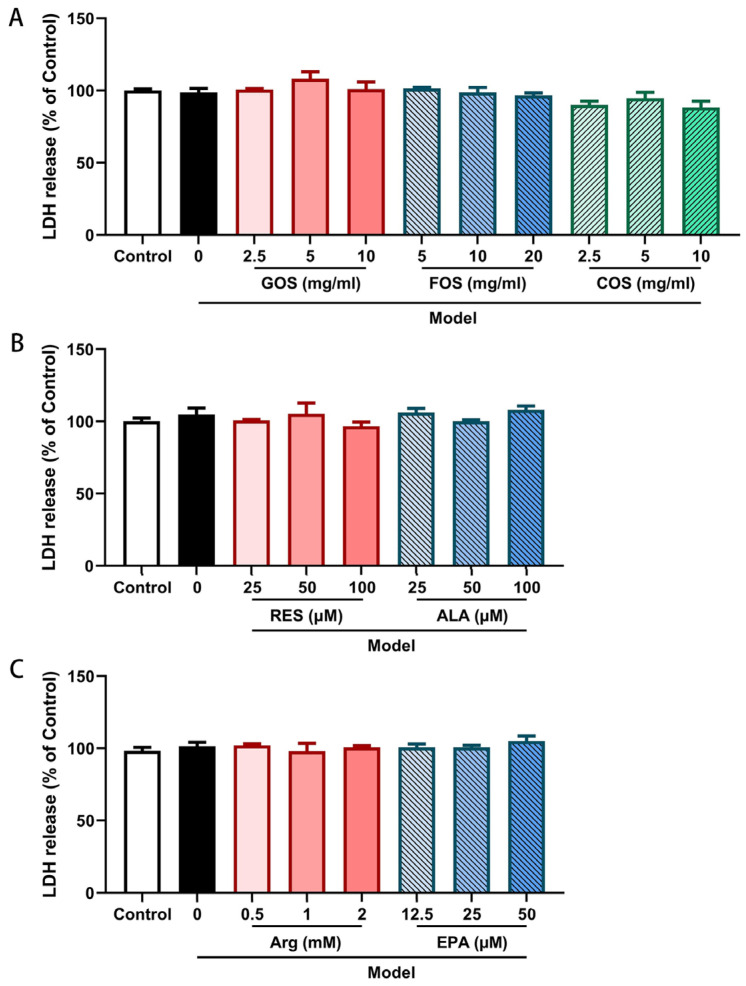
LDH release of Caco-2/HT-29 cell monolayers pre-incubated with GOS, FOS, COS (**A**), RES, ALA (**B**), arginine, and EPA (**C**) for 48 h then exposed to 2 h of hypoxia and heat treatment. LDH release into supernatants was assayed using a LDH assay. All values were presented as means ± SEM (N = 3, n = 2 (GOS, FOS, COS group) and n = 3 (all other groups)). Statistical differences were analyzed by two-way ANOVA followed by the Bonferroni’s multiple comparison test. LDH, lactate dehydrogenase; GOS, galacto-oligosaccharides; FOS, fructo-oligosaccharides; COS, chitosan oligosaccharides; ALA, α-lipoic acid; RES, resveratrol; Arg, l-arginine; EPA, eicosapentaenoic acid. The detailed raw OD data are listed in Table S1.

Cytotoxicity of hypoxia and heat exposure in the presence of the promising nutritional components in Caco-2/HT-29 monolayers was further analyzed through the determination of LDH release at the end of the exposure time. No significant differences were observed between the different groups: control versus heat/hypoxia (model) and control versus model with the nutritional components (Figure 1). The results demonstrated that either heat/hypoxia exposure alone or combined with 48 h pre-incubation with one of the nutritional components did not result in lethal damage to the Caco-2/HT-29 cell monolayers.

### 2.2. GOS, FOS, COS, Arg, RES, ALA and EPA Prevent the Heat/Hypoxia-Induced TEER Decrease in Caco-2/HT-29 Cell Monolayers

**Figure 2:**
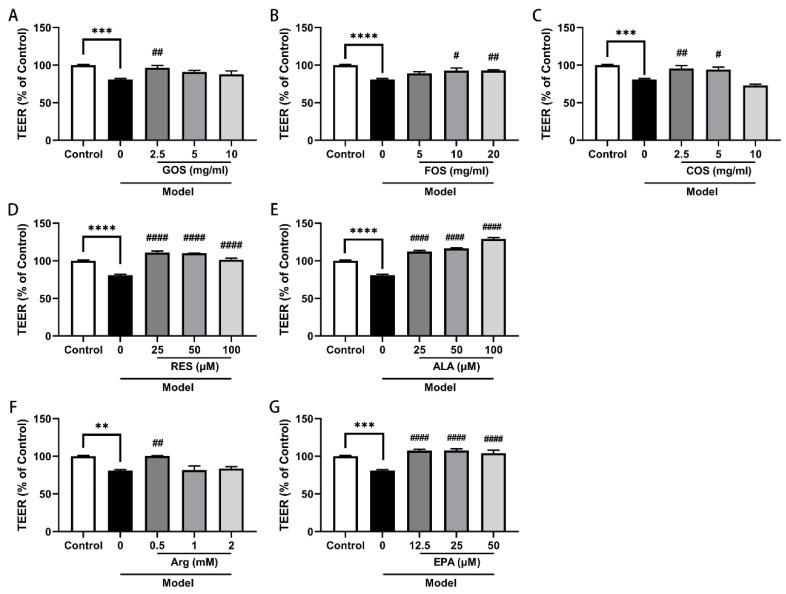
Relative TEER values of Caco-2/HT-29 cell monolayers pre-incubated with GOS (**A**), FOS (**B**), COS (**C**), RES (**D**), ALA (**E**), Arg (**F**), and EPA (**G**) for 48 h then exposed to hypoxia and heat treatment (2 h). After heat/hypoxia exposure, TEER values were determined by using an epithelial volt-ohm meter. All values were presented as means ± SEM (N = 3, n = 3). Same control and model values were depicted in separate figures for different nutritional components. Statistical differences were analyzed by two-way ANOVA followed by the Bonferroni’s multiple comparison test. ** *p* < 0.01, *** *p* < 0.001, and **** *p* < 0.0001 versus control; # *p* < 0.05, ## *p* < 0.01, and #### *p* < 0.0001 versus model. TEER, trans-epithelial electrical resistance; GOS, galacto-oligosaccharides; FOS, fructo-oligosaccharides; COS, chitosan oligosaccharides; ALA, α-lipoic acid; RES, resveratrol; Arg, l-arginine; EPA, eicosapentaenoic acid. The detailed raw TEER readings are listed in Table S2.

TEER values are strong indicators of epithelial cell barrier integrity and permeability. The results showed that after 2 h of hypoxia and heat treatment, TEER values of the co-culture model decreased significantly, which indicates an increased leakiness of the monolayer (Figure 2). Forty-eight hours of pre-treatment with GOS (2.5 mg/mL, Figure 2A), FOS (10 and 20 mg/mL, Figure 2B), COS (2.5 and 5 mL/mg, Figure 2C), RES (25, 50, and 100 μM, Figure 2D), ALA (25, 50, and 100 μM, Figure 2E), Arg (0.5 mM, Figure 2F), and EPA (12.5, 25, and 50 μM, Figure 2G) prevented the heat/hypoxia-induced TEER decrease. It is worth mentioning that after 48 h of pre-treatment, all these nutritional components (and DHA) already significantly increased the TEER values prior to hypoxia and heat exposure (Supplementary Figure S2A–C,E,G–I). 

No significant effect was observed in the Glu- and DHA-treated group after heat/hypoxia treatment (Supplementary Figure S2D,I). Therefore, Glu and DHA-treated groups were excluded from the subsequent measurements.

### 2.3. GOS, FOS, COS, ALA, and EPA Decrease the Heat/Hypoxia-Induced Paracellular Lucifer Yellow Flux in Caco-2/HT-29 Monolayers

**Figure 3:**
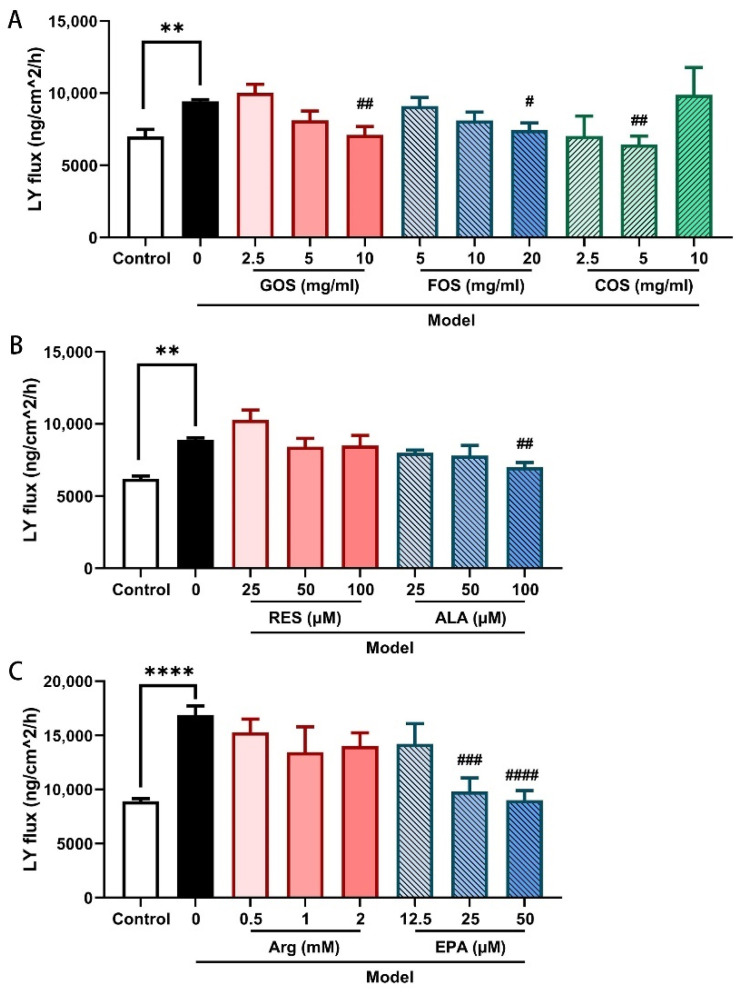
Lucifer Yellow flux of Caco-2/HT-29 cell monolayers pre-incubated with GOS, FOS, COS, (**A**), RES, ALA (**B**), Arg, and EPA (**C**) for 48 h then exposed to hypoxia and heat treatment (2 h). After hypoxia and heat treatment, Lucifer Yellow was added to the apical compartment, and the fluorescent intensity of the medium in the basolateral compartment was measured 4 h after application. All values were presented as means ± SEM (N = 3, n = 2 (GOS, FOS, COS group), and n = 3 (all other groups)). Statistical differences were analyzed by two-way ANOVA followed by the Bonferroni’s multiple comparison test. ** *p* < 0.01 and **** *p* < 0.0001 versus control; # *p* < 0.05, ## *p* < 0.01, ### *p* < 0.001, and #### *p* < 0.0001 versus model. LY, Lucifer Yellow; GOS, galacto-oligosaccharides; FOS, fructo-oligosaccharides; COS, chitosan oligosaccharides; ALA, α-lipoic acid; RES, resveratrol; Arg, l-arginine; EPA, eicosapentaenoic acid. The detailed fluorescent readings are listed in Table S3.

The barrier function was also evaluated by the flux of LY (0.4 kD), an indicator of paracellular permeability, across the epithelial cell layers. After 2 h of hypoxia and heat exposure, LY flux increased significantly in Caco-2/HT-29 cell monolayer (Figure 3). This increase in LY flux was attenuated by pre-incubation with GOS (10 mg/mL, Figure 3A), FOS (20 mg/mL, Figure 3A), COS (5 mg/mL, Figure 3A), ALA (100 μM, Figure 3B), and EPA (25 and 50 μM, Figure 3C) for 48 h. Although RES and Arg prevented the heat/hypoxia-induced TEER decrease, these nutritional components did not significantly affect the increased LY permeability induced by hypoxia and heat exposure.

### 2.4. GOS, ALA, and EPA Prevent the Heat/Hypoxia-Induced Decrease in ZO-1 Protein Expression in Caco-2/HT-29 Cell Monolayers

**Figure 4:**
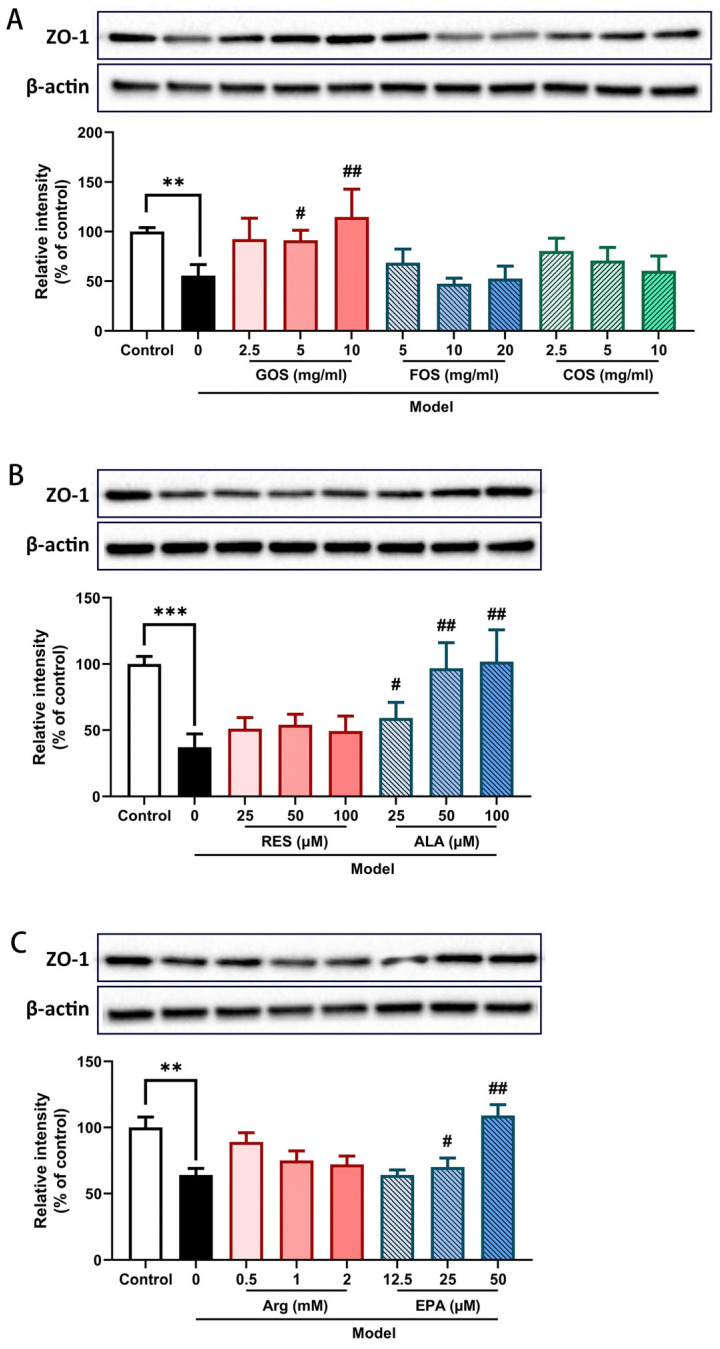
Relative ZO-1 protein expression level in Caco-2/HT-29 cell monolayers pre-incubated with GOS, FOS, COS (**A**), RES, ALA (**B**), Arg, and EPA (**C**) for 48 h then exposed to hypoxia and heat treatment (2 h). ZO-1 protein expression was determined by WB and normalized to β-actin. All values were presented as means ± SEM (N = 3, n = 2 (GOS, FOS, COS group), and n = 3 (all other groups)). Statistical differences were analyzed by two-way ANOVA followed by the Bonferroni’s multiple comparison test. ** *p* < 0.01 and *** *p* < 0.001 versus control; # *p* < 0.05 and ## *p* < 0.01 versus model. GOS, galacto-oligosaccharides; FOS, fructo-oligosaccharides; COS, chitosan oligosaccharides; ALA, α-lipoic acid; RES, resveratrol; Arg, l-arginine; EPA, eicosapentaenoic acid. The original uncropped bands are included in the Supplementary Materials.

ZO-1, an important TJ protein, is a critical structural protein for maintaining intestinal barrier integrity. The ZO-1 protein expression was investigated in the Caco-2/HT-29 co-cultured monolayers via WB analysis. Two hours of heat and hypoxia exposure significantly decreased ZO-1 protein expression (Figure 4). Among all tested components, 5 and 10 mg/mL GOS (Figure 4A), 25–100 μM ALA (Figure 4B), and 25 and 50 μM EPA (Figure 4C) prevented the heat/hypoxia-induced decrease in ZO-1 expression in the Caco-2/HT-29 cells. There was no effect of FOS, COS, RES, and Arg on ZO-1 protein expression in the heat/hypoxia-stimulated Caco-2/HT-29 cell monolayers (Figure 4A–C).

### 2.5. GOS, FOS, ALA, and EPA Prevent the Heat/Hypoxia-Induced Decrease in Claudin-3 Protein in Caco-2/HT-29 Cell Monolayers

**Figure 5:**
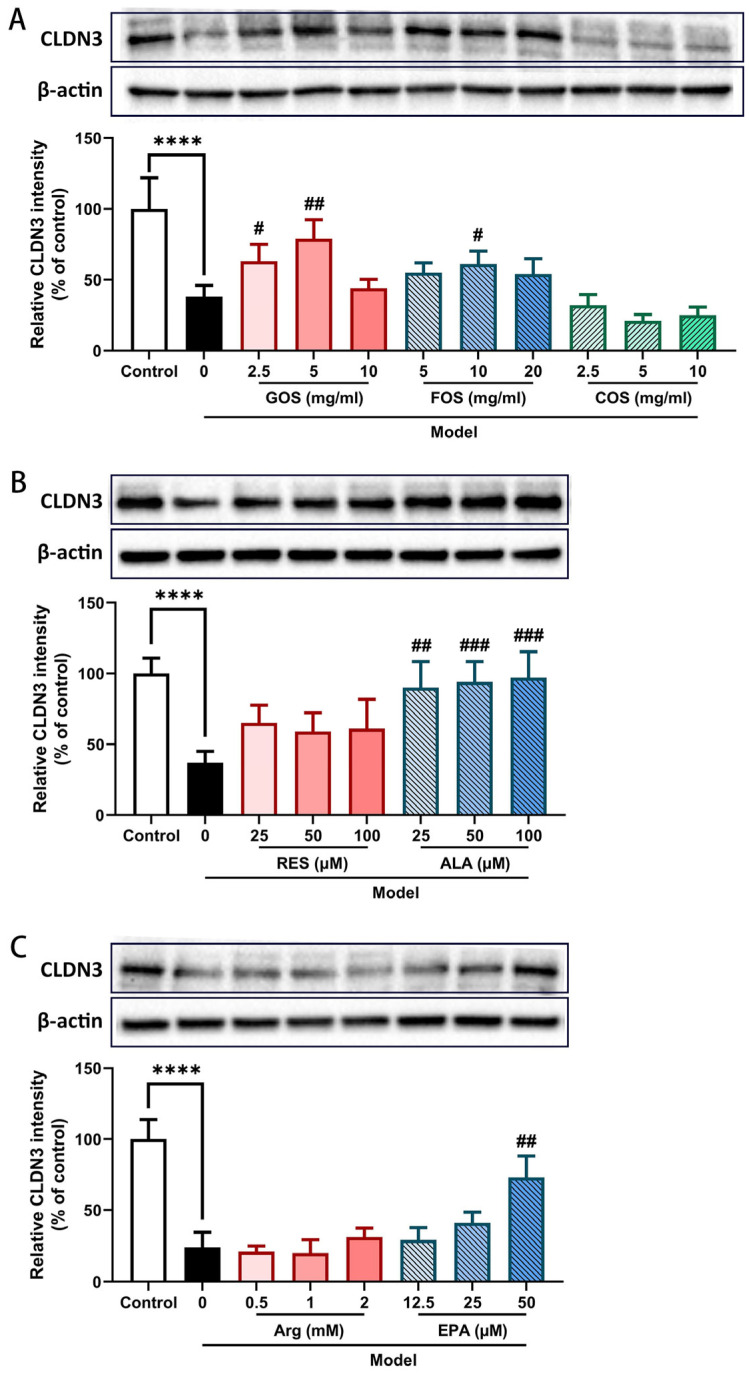
Relative CLDN3 protein expression level in Caco-2/HT-29 cell monolayer pre-incubated with GOS, FOS, COS (**A**), RES, ALA (**B**), Arg, and EPA (**C**) for 48 h then exposed to hypoxia and heat treatment (2 h). CLDN3 protein expression was determined by WB and normalized to β-actin. All values were presented as means ± SEM (N = 3, n = 2 (GOS, FOS, COS group), and n = 3 (all other groups)). Statistical differences were analyzed by two-way ANOVA followed by the Bonferroni’s multiple comparison test. **** *p* < 0.0001 versus control; # *p* < 0.05, ## *p* < 0.01 and ### *p* < 0.001 versus model. GOS, galacto-oligosaccharides; FOS, fructo-oligosaccharides; COS, chitosan oligosaccharides; ALA, α-lipoic acid; RES, resveratrol; Arg, l-arginine; EPA, eicosapentaenoic acid. The original uncropped bands are included in the Supplementary Materials.

In addition to ZO-1, CLDN3 is another member of the junctional complex that regulate paracellular intestinal barrier permeability. The CLDN3 protein expression was examined in the Caco-2/HT-29 co-cultured monolayers via WB analysis. The results showed that 2 h of heat and hypoxia exposure significantly decreased ZO-1 protein expression (Figure 5). Among all tested components, 2.5 and 5 mg/mL GOS (Figure 5A), 10 mg/mL FOS (Figure 5A), 25–100 μM ALA (Figure 5B), and 50 μM EPA (Figure 5C) prevented the heat and hypoxia-induced decrease in CLDN3 expression in the Caco-2/HT-29 cells. No significant effect was observed for COS, RES, and Arg in regulating CLDN3 protein expression in this Caco-2/HT29 co-culture model (Figure 5A–C).

### 2.6. COS, RES and EPA Decrease the Heat/Hypoxia-Induced MDA Production in Caco-2/HT-29 Monolayers

HIF-1α, a critical regulator of the response to hypoxia, was determined via WB analysis. Two hours of heat and hypoxia exposure significantly increased the HIF-1α protein expression in the Caco-2/HT-29 monolayers (Supplementary Figure S3), indicating the occurrence of hypoxic stress. None of the components in this study affected heat/hypoxia-induced HIF-1α accumulation (Supplementary Figure S3).

**Figure 6:**
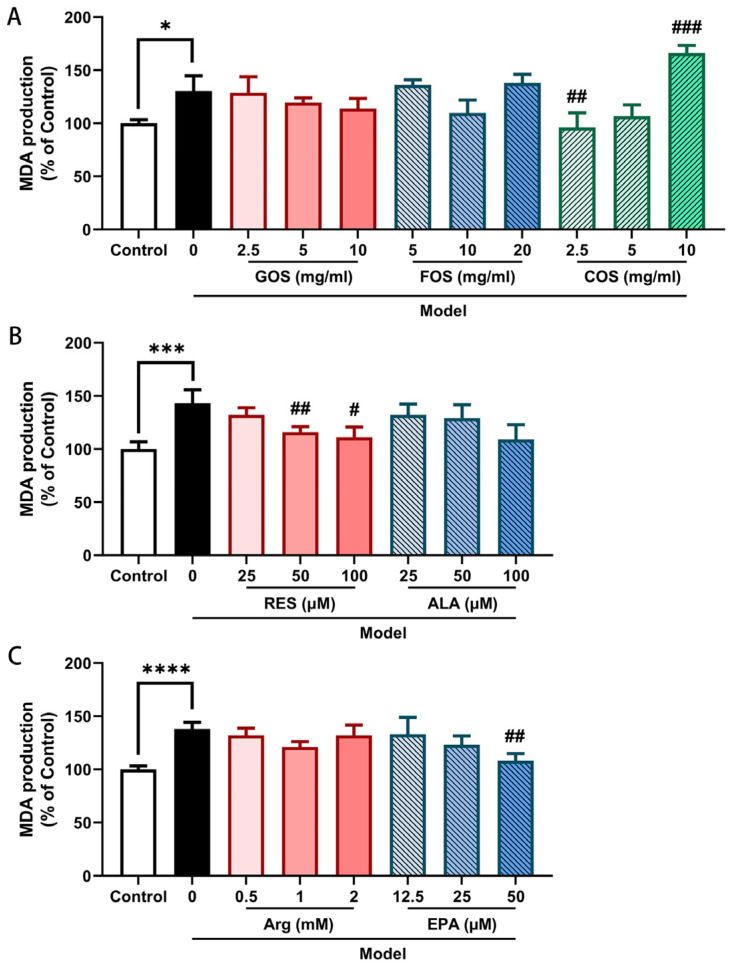
MDA production of Caco-2/HT-29 cell monolayers pre-incubated with GOS, FOS, COS (**A**), RES, ALA (**B**), Arg, and EPA (**C**) for 48 h then exposed to hypoxia and heat treatment (2 h). After hypoxia and heat treatment, MDA in the supernatant was determined by a thiobarbituric acid assay. All values were presented as means ± SEM (N = 3, n = 2 (GOS, FOS, COS group), and n = 3 (all other groups)). Statistical differences were analyzed by two-way ANOVA followed by the Bonferroni’s multiple comparison test. * *p* < 0.05, *** *p* < 0.001, and **** *p* < 0.0001 versus control; # *p* < 0.05, ## *p* < 0.01, and ### *p* < 0.001 versus model. MDA, malondialdehyde; GOS, galacto-oligosaccharides; FOS, fructo-oligosaccharides; COS, chitosan oligosaccharides; ALA, α-lipoic acid; RES, resveratrol; Arg, l-arginine; EPA, eicosapentaenoic acid. The detailed raw OD data are listed in Table S4.

The production of MDA, a biomarker for lipid peroxidation, was used to measure the cellular level of oxidative stress, as a result of hypoxia and subsequent reoxygenation, in the Caco-2/HT-29 co-culture model. The results showed that after 2 h of hypoxia and heat treatment, MDA production was significantly enhanced, indicating that lipid peroxidation occurred in Caco-2/HT-29 monolayers (Figure 6). Pre-incubation with COS (2.5 mg/mL, Figure 6A), RES (50 and 100 μM, Figure 6B), and EPA (50 μM, Figure 6C) for 48 h decreased the heat/hypoxia-induced MDA release compared to the model group. Contrary to the lower COS concentrations, supplementation of 10 mg/mL COS to the heat/hypoxia-exposed cells induced MDA production. The studied concentrations of GOS, FOS, ALA, and Arg did not show a significant effect on heat/hypoxia-induced MDA production (Figure 6A–C).

### 2.7. Arg Further Enhances the Heat/Hypoxia-Induced Increase in HSP-70 Protein Expression in Caco-2/HT-29 Monolayers

**Figure 7:**
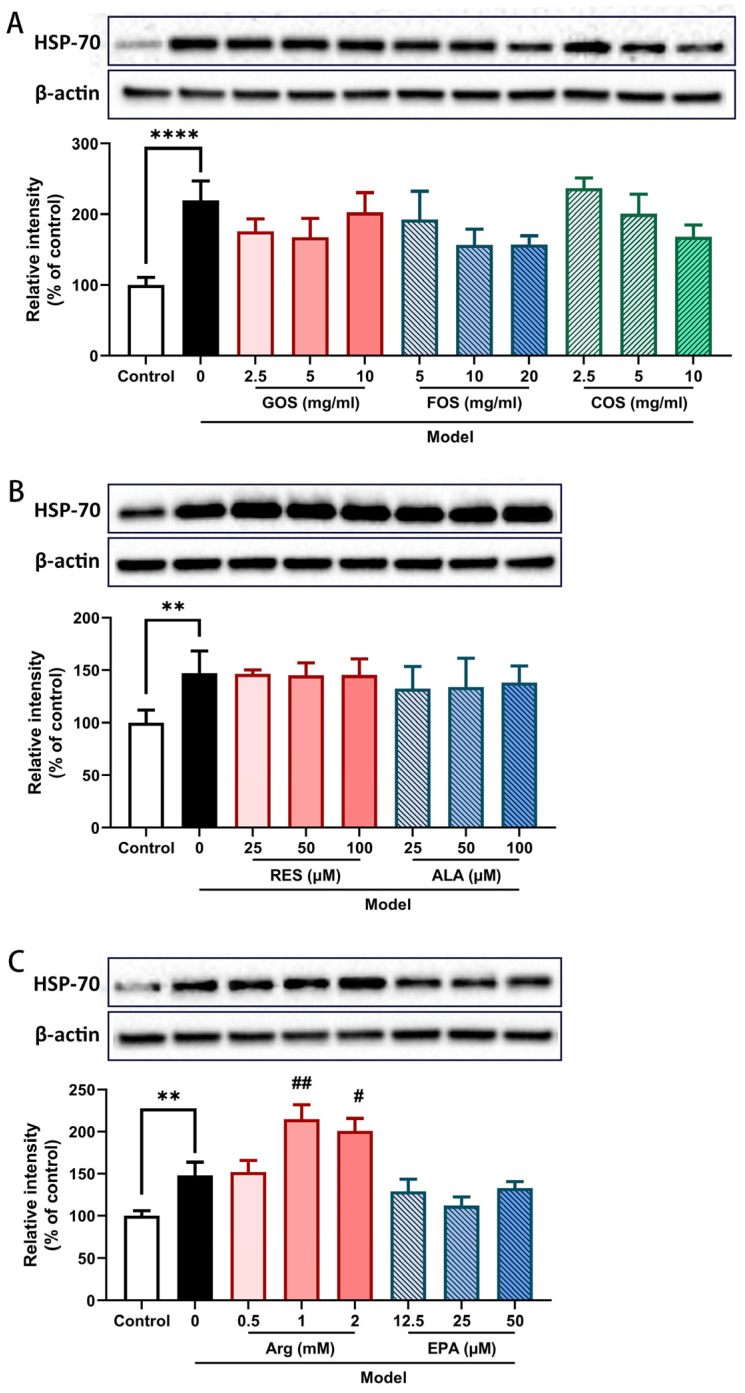
Relative HSP-70 protein expression levels in Caco-2/HT-29 cell monolayer pre-incubated with GOS, FOS, COS (**A**), RES, ALA (**B**), Arg, and EPA (**C**) for 48 h then exposed to hypoxia and heat treatment (2 h). HSP-70 protein expression was determined by WB and normalized to β-actin. All values were presented as means ± SEM (N = 3, n = 2 (GOS, FOS, COS group), and n = 3 (all other groups)). Statistical differences were analyzed by two-way ANOVA followed by the Bonferroni’s multiple comparison test. ** *p* < 0.01 and **** *p* < 0.0001 versus control; # *p* < 0.05 and ## *p* < 0.01 versus model. GOS, galacto-oligosaccharides; FOS, fructo-oligosaccharides; COS, chitosan oligosaccharides; ALA, α-lipoic acid; RES, resveratrol; Arg, l-arginine; EPA, eicosapentaenoic acid. The original uncropped bands are included in the Supplementary Materials.

HSP-70 performs chaperone functions in protein folding and can protect cells from the adverse effects of (heat) stress. HSP-70 expression was determined by WB analysis. HSP-70 levels were significantly enhanced after 2 h of heat and hypoxia exposure compared to control Caco-2/HT-29 monolayers (Figure 7). High concentrations of Arg pre-treatment (1 and 2 mM) further increased HSP-70 expression (Figure 7C). The other nutritional components did not significantly alter the heat/hypoxia-induced increase in HSP-70 expression (Figure 7A–C).

The aforementioned effects of the nine nutritional components on preserving epithelial integrity, anti-lipid peroxidation, and regulating heat shock response are summarized in Table 1.

## 3. Discussion

In this study, an in vitro co-culture model comprising two human colonic cell lines, Caco-2 and HT-29, was used to investigate the effect of nine nutritional components on heat/hypoxia-induced intestinal epithelial injury by measuring intestinal barrier function, oxidative stress, and heat shock responses. We have reviewed potential promising nutritional intervention strategies and found evidence that non-digestible oligosaccharides GOS, FOS, and COS, antioxidants ALA and RES, amino acids Glu and Arg, and PUFAs DHA and EPA might be potential candidates to prevent HS/hypoxia-induced alterations in the integrity of the intestinal epithelial barrier and support intestinal homeostasis [3]. 

Our co-culture model consists of 10% HT-29 cells and 90% Caco-2 cells to simulate the environment of the human intestine. HT-29 cells are mucus-producing cells and represent the specialized secretory cells present in the intestinal epithelium, such as Goblet cells [14]. Goblet cells make up 10–20 percent of total intestinal cells in the epithelium and produce mucins to form a mucosal layer, which is an important physical and chemical barrier to preserve intestinal epithelial integrity [15]. The human intestinal absorptive enterocytes, the Caco-2 cells, form confluent monolayers consisting of well-polarized columnar cells representing a tight intestinal epithelial barrier [16]. Several previous studies have examined the optimal Caco-2:HT-29 ratio for in vitro studies mimicking the in vivo intestinal anatomy and physiology. The physiologically most relevant ratios are considered between 9:1 and 7:3 (Caco-2/HT-29), where TEER, indicative of the barrier properties of the monolayer, reached values quite similar to the human intestine [17,18,19]. Our previous study demonstrated that the addition of 25% HT-29 cells significantly reduced the TEER values of the Caco-2 monolayer and increased paracellular permeability, while 10% HT-29 cells had no effect on the epithelial integrity compared to only Caco-2 cells. The monolayer consisting of Caco-2/HT-29 in a 9:1 ratio expressed mucins, TJ/AJs and heat shock, and oxidative stress-related proteins and genes [5]. Therefore, this Caco-2/HT-29 co-culture model is a valid and versatile model for studying detrimental effects of heat and/or hypoxia on intestinal barrier function.

### 3.1. HSP-70, a Key Regulatory Protein in the HS Response Process Induced by Heat and Hypoxia

Generally, environmental heat or strenuous exercise results in vasodilation and blood re-distribution in the body, leading to hypoxia in different organs and tissues, including the intestinal epithelium [20]. Two hours of heat/hypoxia exposure resulted in a significantly increase in HSP-70 (Figure 5) and HIF-1α (Supplementary Figure S3) expression, indicating the occurrence of heat stress and hypoxia in this Caco-2/HT-29 co-culture model. The expression of HIF-1α, the key regulator of the cellular hypoxic response, can be upregulated by heat and is essential for heat acclimatisation [21]. HIF-1α mRNA levels significantly increased after 5 days of heat stress in the ilea of chickens [8]. On the other hand, hypoxia/reoxygenation increased superoxide generation, which induced cellular protein damage and protein HSP-70 production in Caco-2 cells [22]. Both protective proteins, HSP-70, and HIF-1α display a (synergistic) regulatory role in the heat shock and oxidative stress responses, which contribute to an increased resilience to these stressors [3].

In this study, none of the nine nutritional components affected heat/hypoxia-induced HIF-1α or HSP-70 protein expression, except Arg, which further enhanced the HSP-70 protein expression in heat/hypoxia-stimulated Caco-2/HT-29 cells (Figure 5C). HSP-70 is an important modulator of intestinal epithelial barrier function, performing chaperone functions and helping to protect cells from the adverse effects of physiological stresses [23]. Our previous study demonstrated that ALA stimulates intestinal epithelial recovery after heat stress by enhancing HSP-70 expression in Caco-2 cells [9]. Similarly, other studies proved that enhancement of HSP-70 or administration of exogenous HSP-70 significantly improved intestinal barrier integrity [24,25]. We show for the first time that the amino acid Arg contribute to the regulation of HSP-70 expression under hypoxia and hyperthermia conditions in the co-culture model, playing a potential modulatory role in the stress resilience pathways. HS-induced damage within the intestine is a complicated process involving a benefit–damage balance and a complex modulatory network. Unfortunately, the upregulation of HSP-70 did not lead to improved intestinal epithelial integrity, which might be associated with the inhibition of Glu transport [26,27], as discussed in more detail below. 

### 3.2. TEER Values and TJ Protein Expression, Indicators of Intestinal Epithelial Barrier Integrity

The hypoxia and heat-induced damage to the small intestinal epithelium is reflected by decreased TEER and increased paracellular permeability associated with decreased epithelial TJ protein expression. TJ proteins include claudins, occludins, and zonula occludens (ZO). Claudins and occludins interact with each other on their extracellular sides to promote junction assembly, while the ZO family provides intracellular structural support [28,29]. E-cadherin, the most essential cadherin present on the epithelial surface, is responsible for AJ formation by connecting to E-cadherin on the neighbouring cell [30]. Destruction and dissociation of epithelial TJ/AJ structures leads to increased leakage of luminal toxins or bacteria into blood circulation [31]. Therefore, attenuating or preventing the disruption of TJ/AJ protein breakdown in epithelial cells is critical for increasing cellular adaptation to hypoxia and heat. Our previous studies proved the beneficial effects of GOS, antioxidant ALA, and amino acid Arg in maintaining and supporting the intestinal homeostasis under heat stress conditions in Caco-2 cells [7,9,32].

Here, we observed that seven nutritional components (GOS, FOS, COS, RES, ALA, Arg, and EPA) prevented the heat/hypoxia-induced TEER decrease (Figure 2). After 48 h of pre-treatment, all these nutritional components (and DHA) already significantly increased the TEER values prior to hypoxia and heat exposure (Supplementary Figure S2). These results indicate that these nutritional components not only “treated” intestinal epithelial damage after heat/hypoxia exposure, but also exhibited a direct and preventive beneficial effect on barrier tightness prior to heat/hypoxia exposure. 

In agreement with the TEER observations, GOS, FOS, COS, ALA, and EPA also attenuated the LY paracellular permeability (Figure 3). WB results showed that GOS, FOS, ALA, and EPA prevented the heat/hypoxia-induced decrease in claudin-3 and ZO-1 TJ protein expression in Caco-2/HT-29 cells (Figure 4 and Figure 5).

Although the lack of in-depth mechanistic studies, there are some indications how non-digestible oligosaccharides, such as GOS, FOS, and COS, can reinforce intestinal epithelial integrity and TJ function. This reinforcing effect is mediated via direct interaction with epithelial cell receptors, such as Toll-like receptors, via stimulation of intracellular calcium signalling, via inhibition of pathogen-induced mitogen-activated protein kinase (MAPK) and downstream pro-inflammatory nuclear factor kappa B (NF-κB) hypersensitivity, or by promoting TJ re-assembly, for instance via 5′ adenosine monophosphate-activated protein kinase (AMPK) stimulation [33,34,35,36,37]. The antioxidant ALA preserves intestinal barrier integrity, in part, by regulating oxidative stress as oxidative stress is associated with a tyrosine-kinase-dependent dissociation of E-cadherin—β-catenin and occludin—ZO-1 complexes, which leads to barrier integrity loss [38]. The results related to members of the omega-3 fatty acid family, EPA, and DHA, are in agreement with two studies of Xiao et al., who showed that EPA is more potent than DHA in protecting against heat-induced permeability dysfunction and intestinal epithelial TJ damage in Caco-2 cells as well as in rats [39]. This protective effect of DHA might be related to inhibition of the necroptosis signalling pathway [40,41]. The improved intestinal barrier function induced by Arg observed in rats and IEC-6 cells during heat stress might be associated with AMPK signalling and promoting autophagy [42]. 

In addition to these direct barrier-protective mechanisms, suppressing local inflammatory responses might also play a role in ameliorating intestinal epithelial injury and enhancing intestinal barrier function as these nutritional components also demonstrate immune-regulatory and anti-inflammatory properties [3]. Further research is required to better understand the regulation of intestinal epithelial integrity by these nutritional components. 

Surprisingly, the lower concentrations of GOS, COS, and Arg were more effective in preventing the heat/hypoxia-induced TEER decrease as compared to the higher concentrations. The decreased mitochondrial activity (Supplementary Figure S3) and the increased lipid peroxidation as measured by increased MDA levels in heat/hypoxia-stimulated cells incubated with the higher COS concentration (10 mg/mL) (Figure 6) might explain this lower efficacy of 10 mg/mL COS on intestinal epithelial barrier integrity. 

The TEER values after GOS incubation were not in agreement with the LY flux data: the lowest GOS concentration restored the heat/hypoxia-decreased TEER values, while only the highest GOS concentration attenuated the paracellular LY permeability. Although these findings are difficult to interpret, TEER measurement is more sensitive as it captures changes in ionic permeability, implying that a small leakage is sufficient to alter TEER values [43]. In contrast, the LY flux is only affected when the intestinal epithelial damage is sufficient to cause leakage of this 0.4 kDa molecule. 

Furthermore, our study showed that 1.4 mM to 2.4 mM Arg (including the 0.4 mM Arg already present in the medium) is not beneficial to heat/hypoxia-induced epithelial barrier dysfunction in intestinal epithelial cells in contrast to 0.9 mM Arg. Supra-physiological concentrations of Arg (>10 mM) are reported to be deleterious in the intestine [44,45]. In almost every cell type, Glu transport is partly inhibited by Arg [27]; therefore, it might be possible that the high Arg concentrations affect the basic Glu uptake from the culture medium. Glu is important for maintaining the intestinal mucosal barrier [46,47]. Moreover, the main glutamine transporter alanine-serine-cysteine transporter 2 (ASCT2) was significantly down-regulated in hypoxia-exposed Caco-2 cells [26], and deprivation of glutamine decreased expression of multiple TJ proteins, including ZO-1, in Caco-2 cells [48]. This can explain why no significant protective effect was observed in heat/hypoxia-stimulated Caco-2/HT-29 cells incubated with Glu in the current study (Supplementary Figure S2). This may also (partly) clarify why high concentrations of Arg induced HSP-70 protein expression under heat/hypoxia environment, but neither enhanced TEER values nor elevated tight junction protein expression. These findings point to an extreme narrow therapeutic window for these nutritional components, which should be further investigated and be taken into account in future in vivo or clinical studies.

### 3.3. Oxidative Stress, One of the Sources of Hypoxia and Heat Shock-Induced Tissue Injury

Under hypoxia/reoxygenation and hyperthermia conditions, the oxidative stress and heat shock responses induce excessive production of reactive oxygen species and reactive nitrogen species causing (intestinal) inflammation and tissue damage [49]. In this study, COS, RES, and EPA reversed the heat/hypoxia-induced increase in MDA, a product of lipid breakdown by oxidative stress, in Caco-2/HT-29 cells, demonstrating a promising role of these components in combating oxidative stress (Figure 4). COS, as non-digestible oligosaccharides, exert antioxidative properties, possibly via modulating the intestinal microbiota composition and subsequently the metabolism of short-chain fatty acids [3,50,51,52]. Here, we found that COS act directly as an antioxidant in this intestinal epithelial co-culture system without the presence of intestinal microbiota. 

The polyphenolic compound RES, as an antioxidant, displays a strong antioxidant and anti-inflammatory capacity [53]. In addition, the PUFA EPA might also act as an antioxidant as EPA supplementation leads to a substantial antioxidant response, which might mainly occur via restoring imbalanced endogenous antioxidant moieties [54,55]. The role of oxidative stress in intestinal barrier dysfunction, e.g., following heat/hypoxia exposure, is well established [56] and therefore, restoring the imbalance of the antioxidative system by nutritional components might contribute to a stronger intestinal epithelial barrier, which can more easily deal with the adverse effects of heat/hypoxia.

### 3.4. Clinical Significance

One quarter to one half of elite athletes suffer from gastrointestinal symptoms, which may deter them from participating in training and competitive events. Strenuous exercise and dehydrated states play an important role in the development of gastrointestinal symptoms referred by 70% of the athletes [57]. Recent research suggests that the gut microbiota may play a role in athlete health and performance [58]. Different studies reported increased intestinal permeability and/or endotoxemia during strenuous exercise and/or heat stress in humans [59,60,61].This is in agreement with the in vitro model described in this study showing heat/hypoxia-induced intestinal epithelial barrier disruption. Moreover, the heat/hypoxia-induced increase in HSP-70 protein expression in this Caco-2/HT-29 model is also in correspondence with several studies showing the upregulation of HSP-70 during different types of exercise in normal or increased temperatures, suggesting a role of HSP-70 in the cellular heat adaptation to heat acclimatization [62,63,64,65]. 

However, we need to be careful with the translation of this in vitro study using two intestinal epithelial cell lines to the more complex in vivo situation and ultimately to future clinic trials. The safety of the multiple nutritional components used in this study has been evaluated in different clinical trials [66,67,68,69,70,71], and these compounds are already available. A systematic review pointed out that in most of the studies probiotics, prebiotics and synbiotics induce positive health effects in athletes and active individuals, but these studies are currently limited in number and quality [72]. Our in vitro data suggest that a combination of non-digestible oligosaccharides (e.g., GOS, FOS), antioxidants (e.g., ALA, RES) and PUFAs (DHA) might be an interesting preventive (or therapeutic) approach to counteract heat/hypoxia-induced intestinal damage in humans. The clinical relevance of these nutritional components, the doses required, and the duration of treatment to exert potential influences on heat/hypoxia-induced intestinal damage remains to be elucidated. 

## 4. Materials and Methods

### 4.1. Cell Culture

Caco-2 (HTB-37™, ATCC, Manassas, VA, USA) and HT-29 (HTB-38™, ATCC, Manassas, VA, USA) cells were separately grown in 75-cm^2^ tissue culture flasks (#430641, Corning, Corning, NY, USA) at 37 °C, 5% CO_2_, and 90% relative humidity environment. The cells were sub-cultured at ~90% confluence (∼6 days) by 0.25% trypsin and 0.02% EDTA solution (#25200056, Thermo Fisher Scientific, Waltham, MA, USA). The DMEM culture medium (#42430025, Thermo Fisher Scientific), supplemented with 10% fetal calf serum (#10099, Thermo Fisher Scientific), 1 × non-essential amino acids (#11140035, Thermo Fisher Scientific), 2 mM L-glutamine (#25030081, Thermo Fisher Scientific), and 1% penicillin and streptomycin (#15140122, Thermo Fisher Scientific), was refreshed every 2 days [5].

### 4.2. Cell Co-Culture

Caco-2 and HT-29 cells were counted by an automated cell counter (Cellometer Auto T4, Nexcelom Bioscience, Lawrence, MA, USA), mixed in a ratio of 9:1 (Caco-2: HT-29) and seeded into the apical chambers of 24-well Transwell™ inserts (#353495, Corning, Corning, NY, USA) with a final density of 1 × 10^5^ cells/cm^2^ in each insert. Cells were cultured as described in Section 4.1 and allowed to grow for 17 days. The medium (300 μL in the apical chamber and 700 μL in the basolateral chamber) was refreshed every 2 days. Caco-2 and HT-29 co-cultures with TEER values > 300 Ω × cm^2^ which reached a plateau (~17 days after seeding) were used for further experiments [5].

### 4.3. Pre-Treatment with the Nutritional Components

Chicory FOS (purity > 97%) were obtained from Orafti (Wijchen, The Netherlands). GOS (Vivinal^®^ GOS Powder, purity > 70%) produced from lactose were provided by Friesland Campina (Amersfoort, The Netherlands). COS (purity >90%) derived from marine biological sources, such as shrimp and crab shells, were purchased from BZ Oligo Biotech (Qingdao, China). FOS, GOS, and COS were dissolved in DMEM medium and sterilized by 0.2 μM pore filters (#431227, Corning, Corning, NY, USA) prior to each experiment. The acidic pH of the prepared COS solution was adjusted to 7.0 (pH neutral) with 1 M NaOH. DHA (#D2534, Sigma Aldrich, St. Louis, MO, USA), EPA (#E2011, Sigma Aldrich), ALA (#T1395, Sigma Aldrich), and RES (#R5010, Sigma Aldrich, St. Louis, MO, USA) stock solutions (100 mM) were prepared with 100% ethanol. Arg (#A8094, Sigma Aldrich) and Glu (#G8540, Sigma Aldrich) stock solutions (400 mM) were prepared with PBS. The stock solutions were sterilised by 0.2 μM pore filters and kept in −20 °C (dark environment) and dissolved in the culture medium prior to each experiment.

### 4.4. Hypoxia and Heat Exposure

Hypoxia was induced by using a multi-functional incubator (Galaxy 48R, Eppendorf AG, Hamburg, Germany) for 2 h. The O_2_ concentration in the chamber was maintained at 5%, with a residual gas mixture composed of 5% CO_2_ and balanced N_2_. For heat exposure, the environmental temperature was set at 42 °C. The relative humidity was kept at 90% as described before [5].

### 4.5. Cytotoxicity Assays

Possible cytotoxicity of the nutritional components on the co-cultured cells was evaluated with the 3-[4,5-dimethylthiazol-2-yl]-2,5-diphenyl-tetrazolium bromide (MTT) assay. At the end of nutritional component pre-treatment (48 h), the cells were refreshed with medium containing 0.1% (*w/v*) MTT (#M5655, Sigma-Aldrich, St. Louis, MO, USA) and incubated for an additional period of 4 h. The amount of violet formazan dye formed from MTT as a marker of changes in mitochondrial activity is proportional to the amount of viable cells. The supernatant was carefully removed, and 200 μL DMSO was added to the cells to terminate the MTT reaction and dissolve the formazan at the end of the 4 h incubation period. The absorbance (λ_ab_ 570 nm) of DMSO containing formazan was read using a microplate spectrophotometer. The group in which all cells were intentionally lysed by lysis buffer was included as a positive control.

Since hypoxia and heat treatment would interfere with mitochondrial function, cytotoxicity of the components after hypoxia and heat treatment was determined via the lactate dehydrogenase (LDH) production, indicating cytomembrane damage and intracellular contents release. After exposure to hypoxia and heat, the supernatants were collected and immediately assayed for cytotoxicity using CytoTox 96^®^ Cytotoxicity Non-Radioactive Assay kit (Promega, Madison, WI, USA) according to manufacturer’s instructions. 

### 4.6. Trans-Epithelial Electrical Resistance (TEER) Measurement

The cell monolayer integrity was determined by TEER measurement using an epithelial volt-ohm meter (Millicell ERS-2, Merck, Rahway, MJ, USA). The electrodes were placed into two chambers of each Transwell™ insert. TEER values > 300 Ω × cm^2^ were regarded as valid for further permeability studies.

### 4.7. Lucifer Yellow (LY) Permeability Test

The fluorescent chemical LY (0.44 kDa, L0144, Sigma-Aldrich) was used to measure the paracellular permeability across the co-culture monolayer. At the end of heat/hypoxia treatment, 30 μL LY (200 μg/mL) was added into the apical chamber of the Transwell™ inserts. The inserts were kept at 37 °C in the dark for 4 h, then the medium from the basolateral chamber was collected for fluorescent intensity measurements (λ_ex_ 428 nm, λ_em_ 540 nm) using a fluorometer (Fluoroskan Ascent^®^ FL, Thermo Fisher Scientific). The fluorescent emission intensity was converted into fluorescein flux per hour by using a standard curve.

### 4.8. Lipid Peroxidation Assay

Lipid peroxidation was determined in the supernatant by the reaction of malondialdehyde (MDA) with thiobarbituric acid (TBA) following the instructions of the Lipid Peroxidation (MDA) Assay Kit (#MAK085, Sigma-Aldrich). The results were presented as MDA production, indicating oxidative attack and cytomembrane damage.

### 4.9. Protein Extraction

For total protein extraction, the cells were lysed using 50 μL Pierce™ RIPA buffer (#89901, Thermo Fisher Scientific) containing protease inhibitor cocktail (#11836170001, Roche, Basel, Switzerland). Total protein concentration was assessed and standardized with Pierce™ BCA Protein Assay Kit according to the manufacturer’s instructions (Thermo Fisher Scientific). The protein samples used for determining hypoxia-inducible factor-1α (HIF-1α) expression were preincubated with dimethyloxalylglycine (DMOG; #D3695, Sigma-Aldrich), a HIF prolyl-hydroxylase inhibitor, which prevents HIF-1α degradation under normoxic conditions.

### 4.10. Western Blot (WB) Analysis

Equal amounts (20 μg) of boiled protein samples were separated by electrophoresis (Criterion™ Gel, 4–20% Tris-HCl, Bio-Rad, Hercules, CA, USA) and electrotransferred onto Trans-Blot^®^ Turbo™ polyvinylidene difluoride (PVDF) membranes (midi format 0.2 μm, Bio-Rad, Hercules, CA, USA). After being blocked with 5% skimmed milk (in PBS containing 0.05% Tween-20 (PBST)), the membranes were incubated overnight at 4 °C with the primary antibodies of HSP-70 (1:1000, #C92F3A5, Enzo life science, Bruxelles , Belgium), HIF-1α (1:2000, #ab113642, Abcam, Cambridge, UK), claudin-3 (CLDN3) (1:1000, #341700, Invitrogen, Waltham, MA, USA), and ZO-1 (1:500, #339100, Invitrogen, Waltham, MA, USA). Housekeeping protein β-actin (1:2000, #13E5, Cell Signaling, Danvers, MA, USA) was also assessed in parallel with each target protein and used for normalization. Thereafter, the membranes were incubated with horseradish peroxidase (HRP)-conjugated secondary antibodies (1:10,000, Dako, Glostrup, Denmark) for 2 h at room temperature. After being rinsed with PBST and incubated with ECL detection reagent (#RPN2235, GE Healthcare, Chicago, IL, USA), the membranes were exposed to ECL imaging system (ChemiDoc MP, Bio-Rad, Hercules, CA, USA). The optical intensity of the blots was recorded and analyzed by using Image Lab (version 6.01, Bio-Rad, Hercules, CA, USA) and ImageJ (version 1.80, NIH, Bethesda, MD, USA) software. Membranes were stripped by using Restore PLUS Western Blot Stripping Buffer (#46430, Thermo Fisher Scientific) and re-blotted with the different primary antibodies.

### 4.11. Statistical Analysis

Results are expressed as means ± SEM of 3 independent experiments (N = 3), each performed in duplicate (or triplicate unless otherwise stated. Statistical analyses were performed by using GraphPad Prism^®^ (version 9.3.1, GraphPad, San Diego, CA, USA). Differences between groups were determined by using two-way analysis of variance (ANOVA), with Bonferroni post-hoc test. Results were considered statistically significant when *p* < 0.05.

## 5. Conclusions

In conclusion, the non-digestible oligosaccharides, especially GOS and FOS, the antioxidant ALA, and the PUFA EPA, exhibited the ability to protect Caco-2/HT29 cells from heat/hypoxia-induced intestinal injury, as these components preserved TEER values, inhibited paracellular LY permeability and increased tight junction protein expression. Amino acid Arg acts more like a “double-edged sword” as the beneficial effect of Arg on intestinal barrier function (TEER) was only confined close to its physiological level, while higher concentrations further enhanced the heat/hypoxia-induced increase in HSP-70 expression. Besides the barrier-protective properties of EPA and antioxidant RES under heat/hypoxia conditions, they also exhibited antioxidative activity. The amino acid Glu and the PUFA DHA were less effective in mitigating heat/hypoxia-induced intestinal injury in this specific Caco-2/HT-29 co-culture model. Combinations of non-digestible oligosaccharides (e.g., GOS, FOS), antioxidants (e.g., ALA, RES), and PUFAs (DHA) might be an interesting preventive (or therapeutic) approach to combat heat/hypoxia-induced intestinal injury and could be an important research area for future in vivo studies and clinical trials.

## Figures and Tables

**Table 1:** The effects of nutritional components on heat/hypoxia-induced intestinal epithelial injury.

	GOS	FOS	COS	Glu	Arg	RES	ALA	DHA	EPA
Preserving epithelial integrity	+++	+++	++	-	+	+	+++	-	+++
Anti-lipid peroxidation	-	-	+	ND	-	+	-	ND	+
Regulating heat shock response	-	-	-	ND	+	-	-	ND	-

-, no significant effect was observed; ND, not determined. “Preserving epithelial integrity”: +, significant effect was observed on TEER values; ++, significant effect was observed on TEER values and LY permeability; +++, significant effect was observed on TEER values, LY permeability, and TJ protein expression. “Anti-lipid peroxidation”: +, MDA production was significantly decreased. “Regulating heat shock response”: +, HSP-70 protein expression was significantly enhanced.

## Data Availability

The datasets used and/or analyzed during the current study are available from the corresponding author on reasonable request.

## Supplementary Materials

The supporting information can be downloaded at: https://www.mdpi.com/article/10.3390/ijms24021111/s1.
