# Supplementary material for: Differential Effects of Oligosaccharides, Antioxidants, Amino Acids and PUFAs on Heat/Hypoxia-Induced Epithelial Injury in a Caco-2/HT-29 Co-Culture Model
Source: Int J Mol Sci. 2023 Jan 6;24(2):1111. doi: 10.3390/ijms24021111 (PMC9861987; doi:10.3390/ijms24021111)
Supplement: Supplementary file 1 [file ijms-24-01111-s001.zip › ijms-2089841-Figure S4-Original images of all blot results.pdf]

Original images of all blot results reported in the main article and the supplementary materials of

“Differential effects of oligosaccharides, antioxidants, amino acids and PUFAs on heat/hypoxia-induced epithelial injury in a Caco-2/HT-29 co-culture model”

Puqiao Lian[1], Paul A. J. Henricks[1], Harry J. Wichers[2], Gert Folkerts[1], Saskia Braber[1]

[1] Division of Pharmacology, Utrecht Institute for Pharmaceutical Sciences, Faculty of Science, Utrecht University, Utrecht, The Netherlands

[2] Food & Biobased Research, Wageningen University, Wageningen, the Netherlands

Corresponding author:

S. Braber

Utrecht University, Department of Pharmaceutical Sciences, Division of Pharmacology

Universiteitsweg 99, 3584 CG, Utrecht, The Netherlands

Telephone: +31 302531599

Email: s.braber@uu.nl

Original blot images supporting Figure 4:

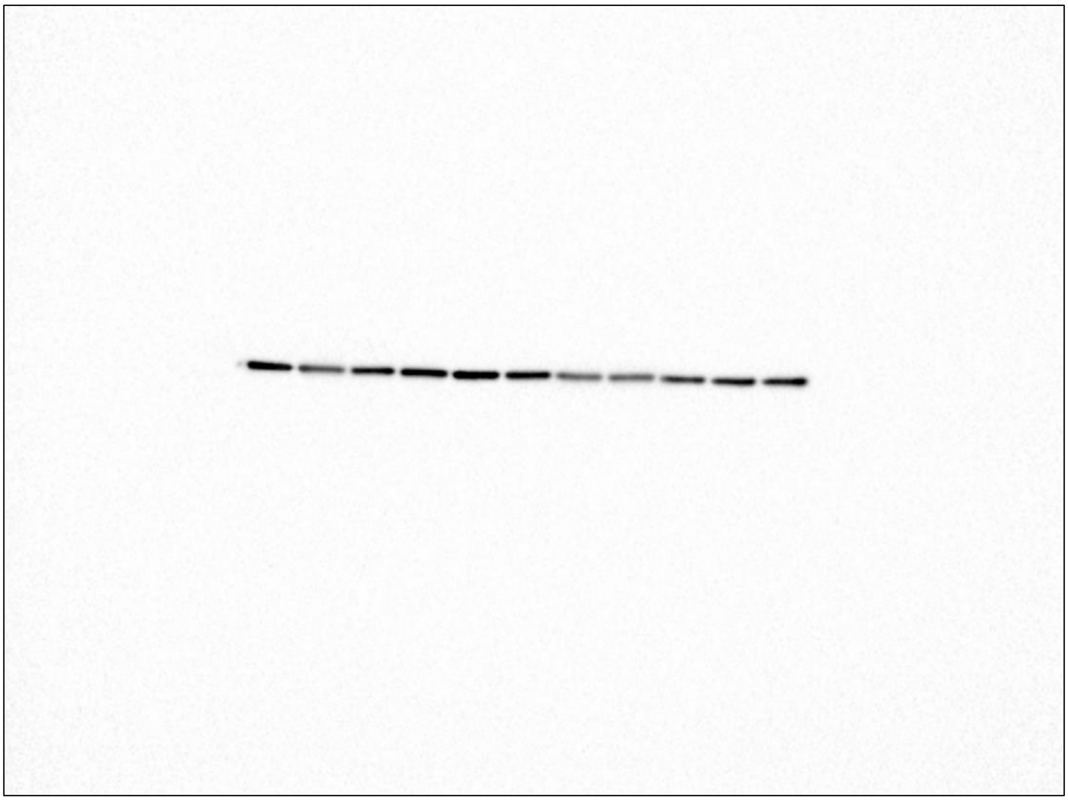

Repeat 1 of ZO-1, lanes from left to right: Control, Model, GOS 2.5, 5, 10 mg/ml, FOS 5, 10, 20 mg/ml, COS 2.5, 5, 10 mg/ml.

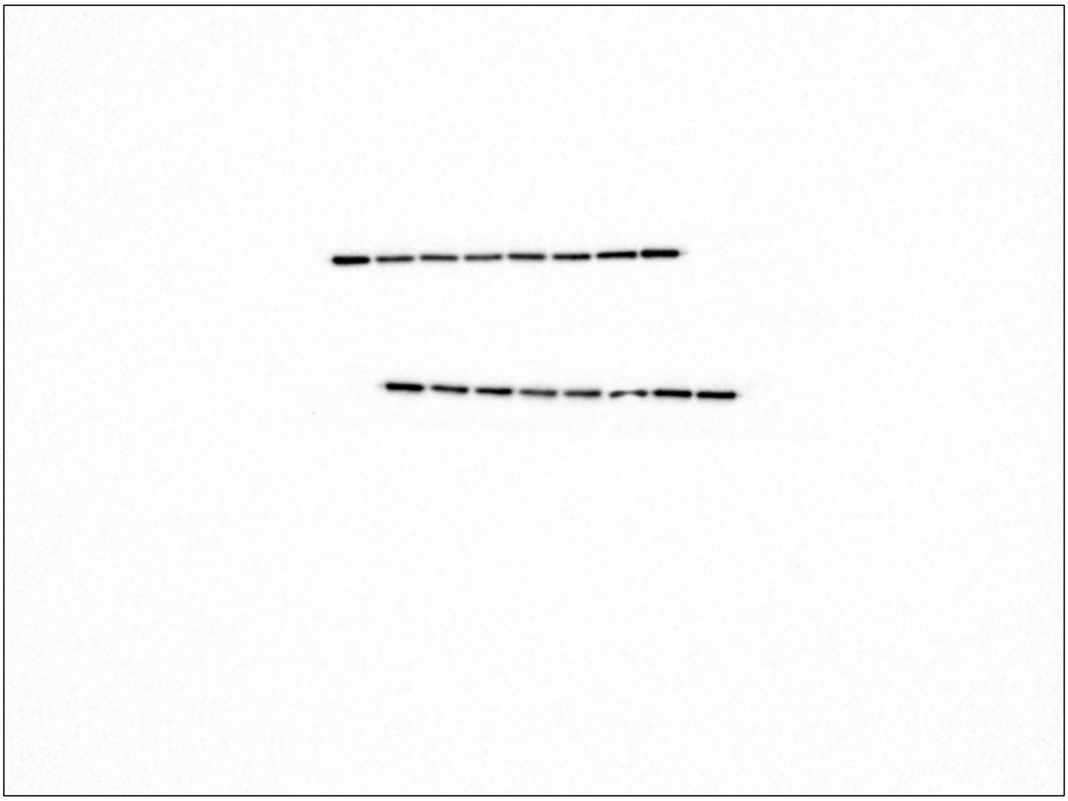

Repeat 1 of ZO-1, upper lanes from left to right: Control, Model, RES 25, 50, 100 µM, ALA 25, 50, 100 µM; lower lanes from left to right: Control, Model, Arg 0.5, 1.0, 2.0 mM, EPA 12.5, 25, 50 µM.

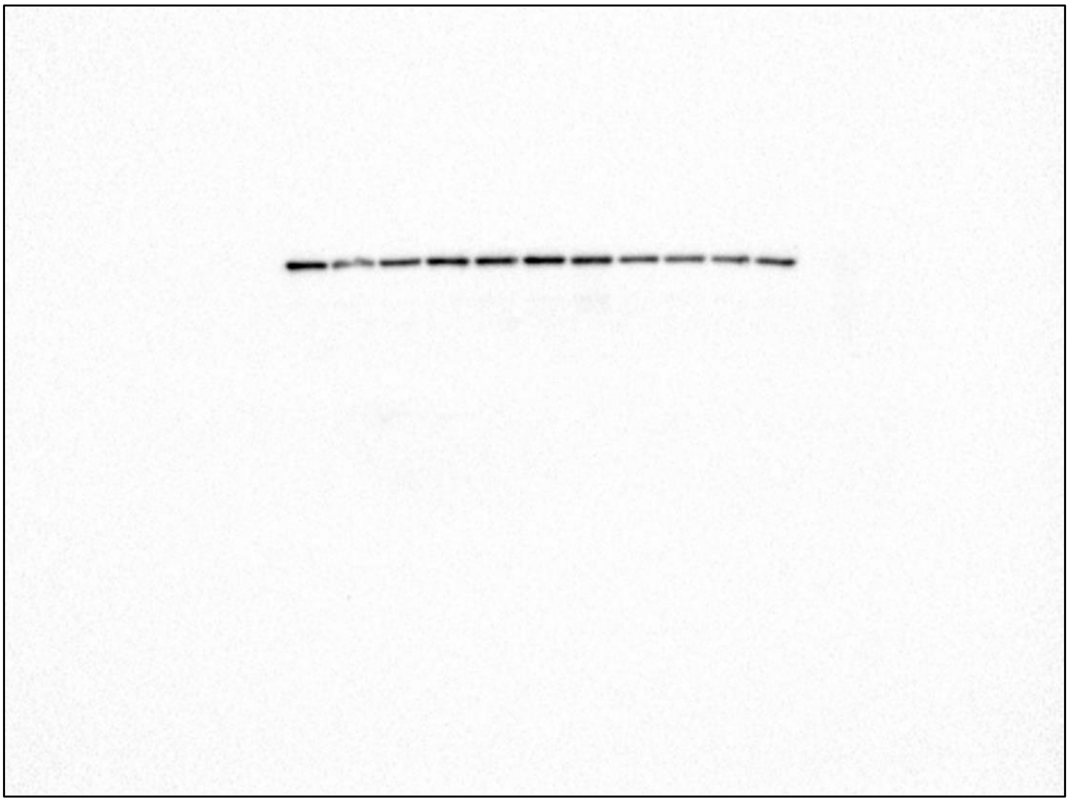

Repeat 2 of ZO-1, lanes from left to right: Control, Model, GOS 2.5, 5, 10 mg/ml, FOS 5, 10, 20 mg/ml, COS 2.5, 5, 10 mg/ml.

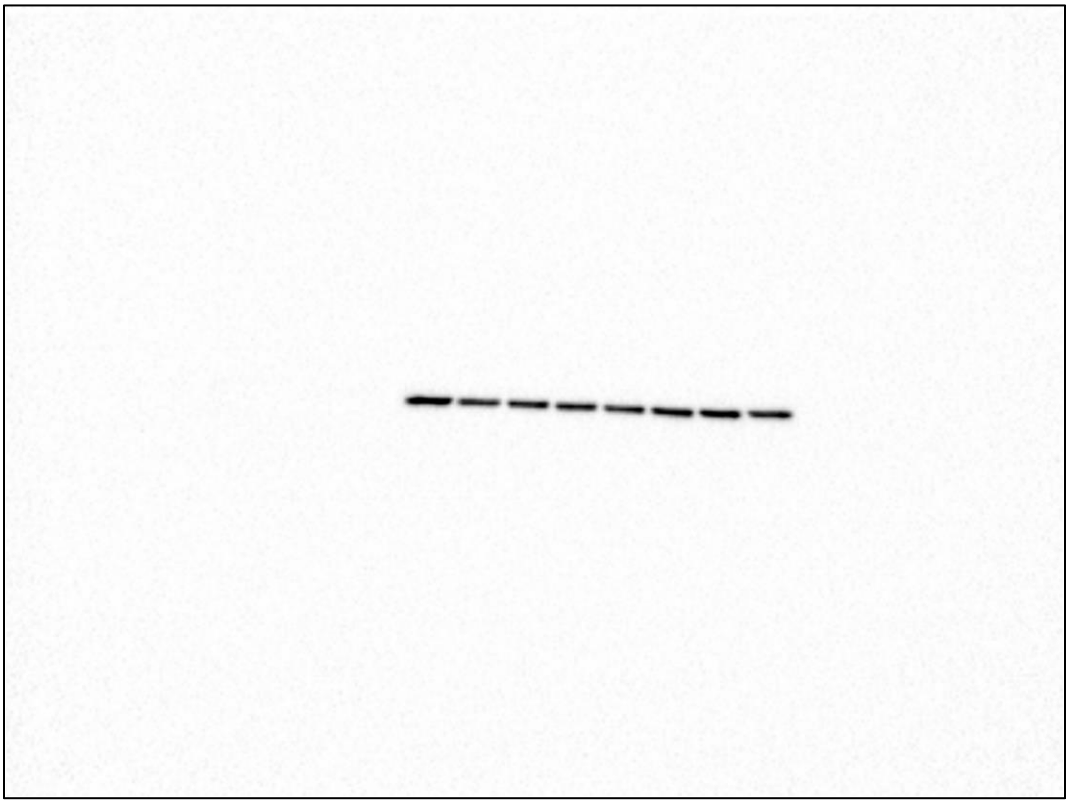

Repeat 2 of ZO-1, lanes from left to right: Control, Model, RES 25, 50, 100 μM, ALA 25, 50, 100 μM;

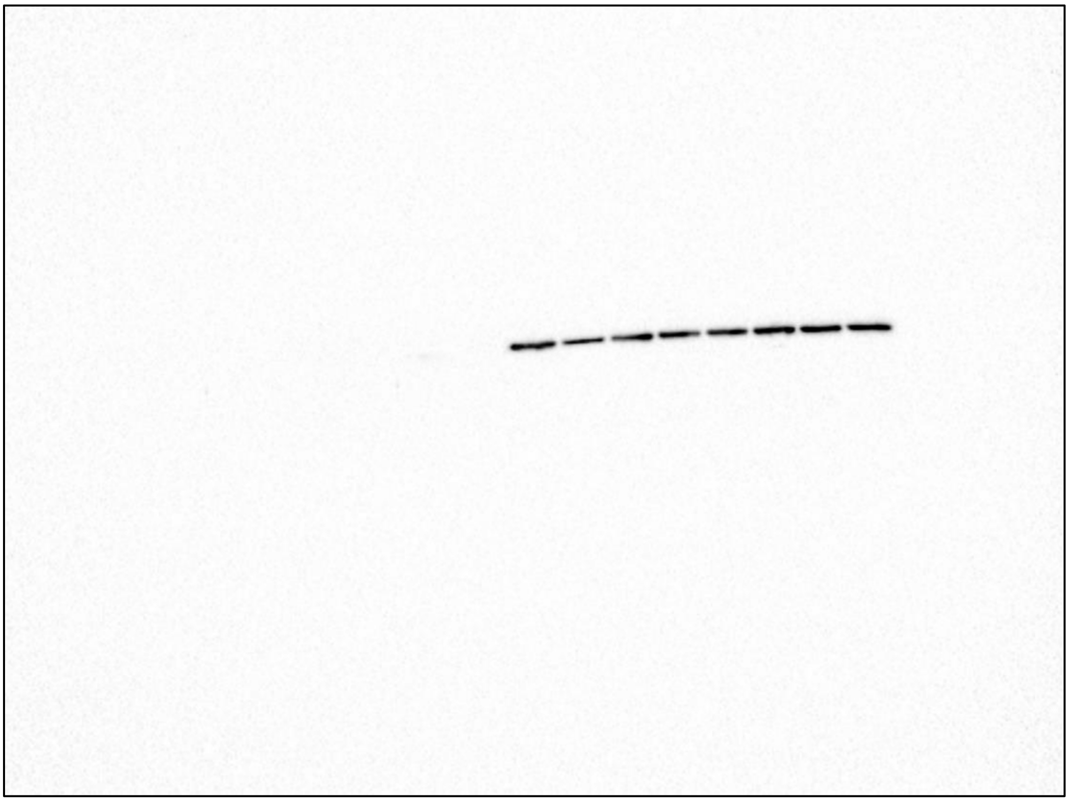

Repeat 2 of ZO-1, lanes from left to right: Control, Model, Arg 0.5, 1.0, 2.0 mM, EPA 12.5, 25, 50 μM.

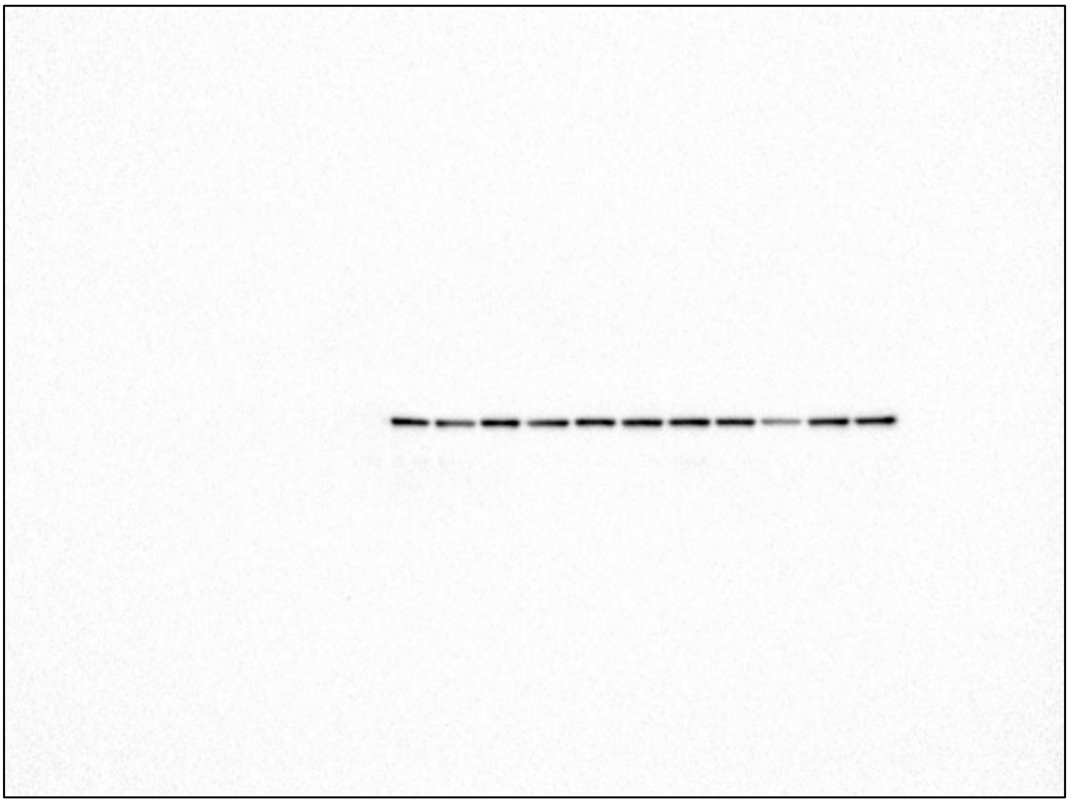

Repeat 3 of ZO-1, lanes from left to right: Control, Model, GOS 2.5, 5, 10 mg/ml, FOS 5, 10, 20 mg/ml, COS 2.5, 5, 10 mg/ml.

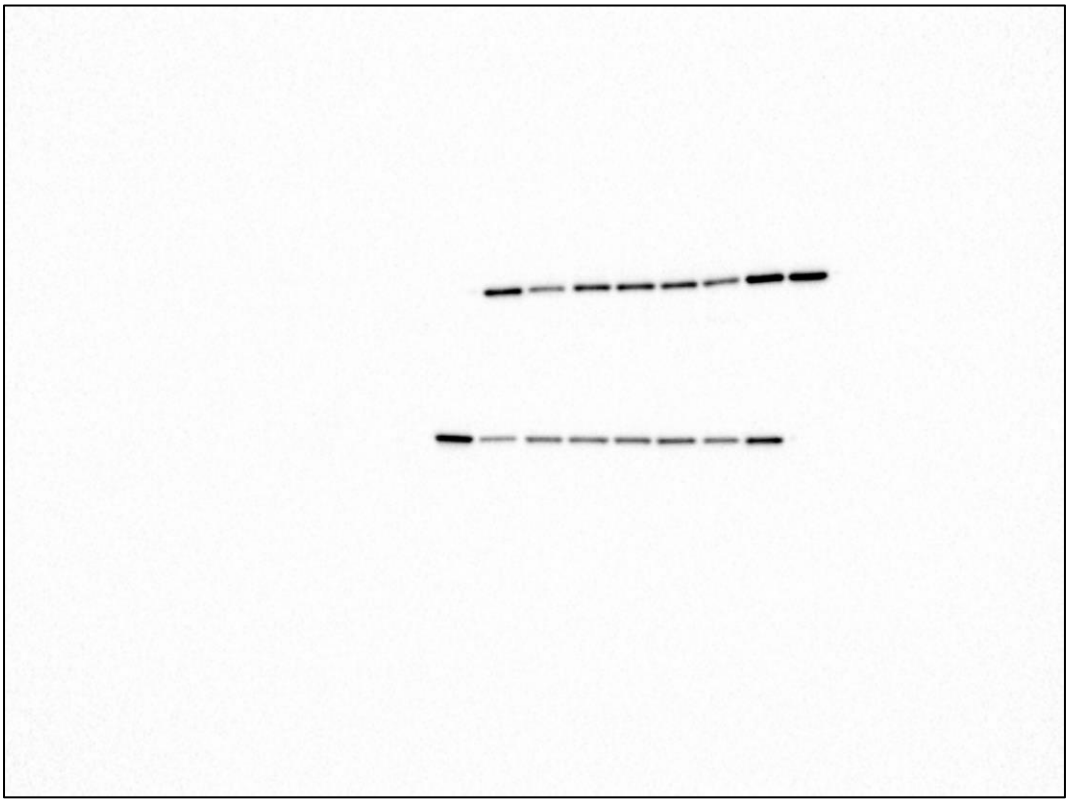

Repeat 3 of ZO-1, upper lanes from left to right: Control, Model, RES 25, 50, 100 μM, ALA 25, 50, 100 μM; lower lanes from left to right: Control, Model, Arg 0.5, 1.0, 2.0 mM, EPA 12.5, 25, 50 μM

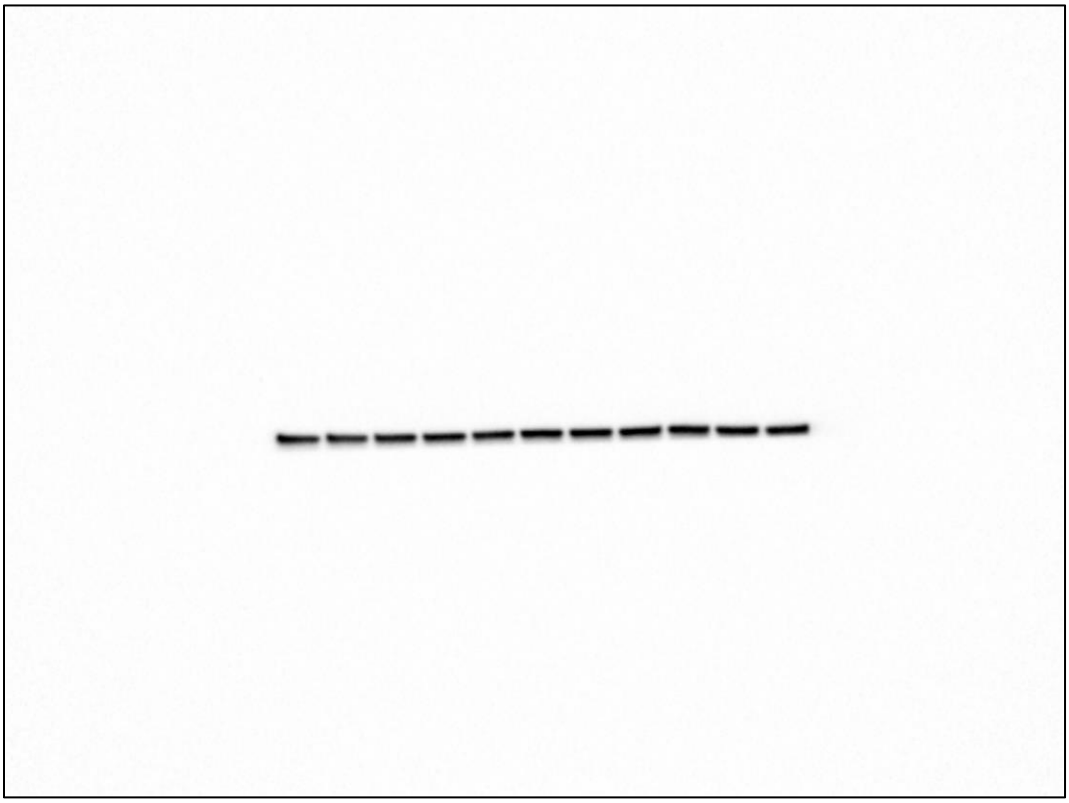

Repeat 1 of β-actin, lanes from left to right: Control, Model, GOS 2.5, 5, 10 mg/ml, FOS 5, 10, 20 mg/ml, COS 2.5, 5, 10 mg/ml.

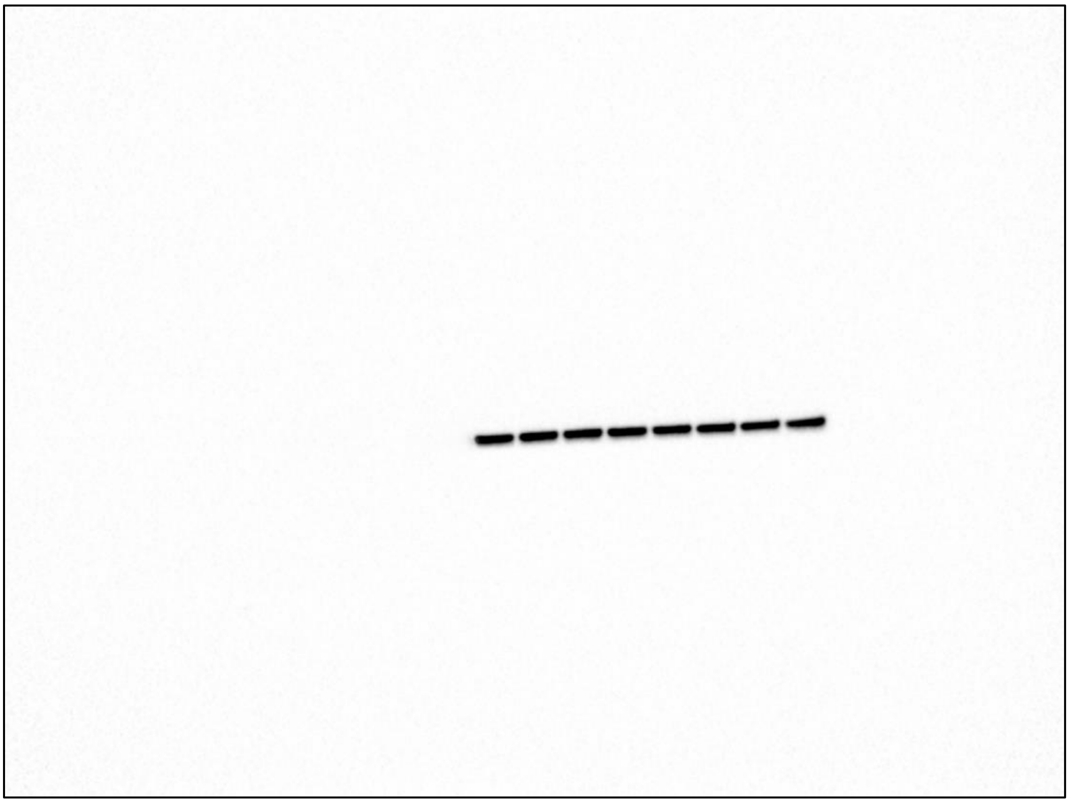

Repeat 1 of β-actin, lanes from left to right: Control, Model, RES 25, 50, 100 µM, ALA 25, 50, 100 µM.

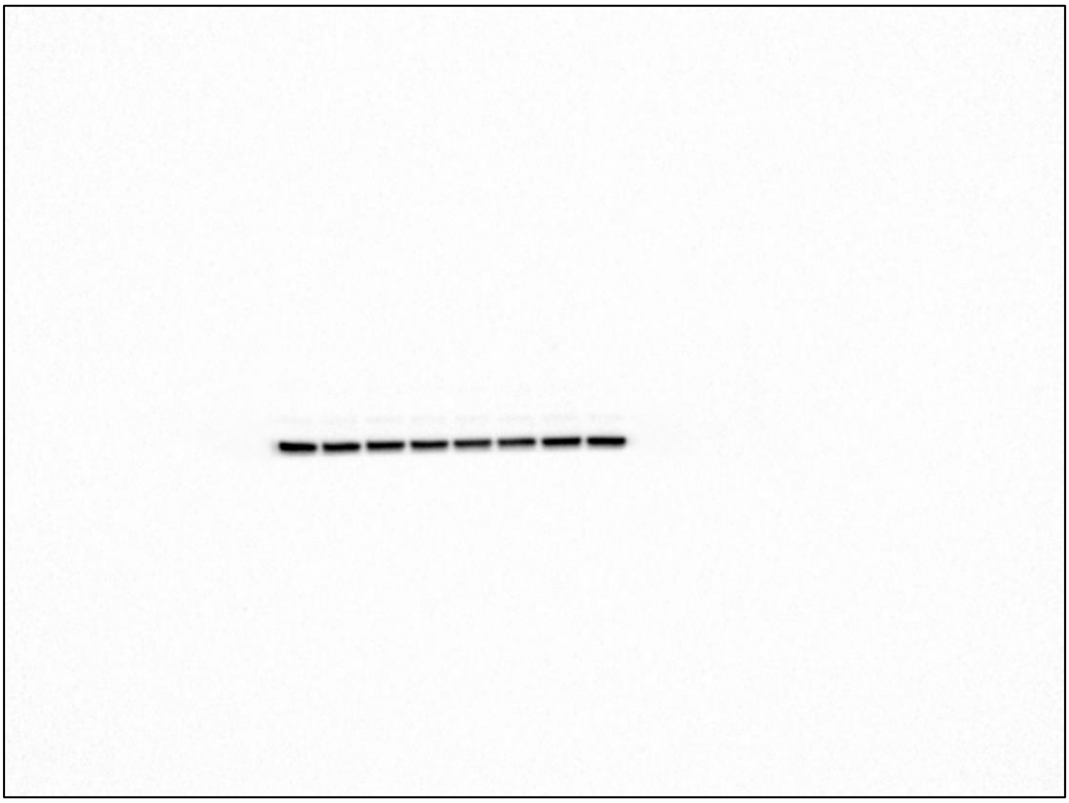

Repeat 1 of β-actin, lanes from left to right: Control, Model, Arg 0.5, 1.0, 2.0 mM, EPA 12.5, 25, 50 µM.

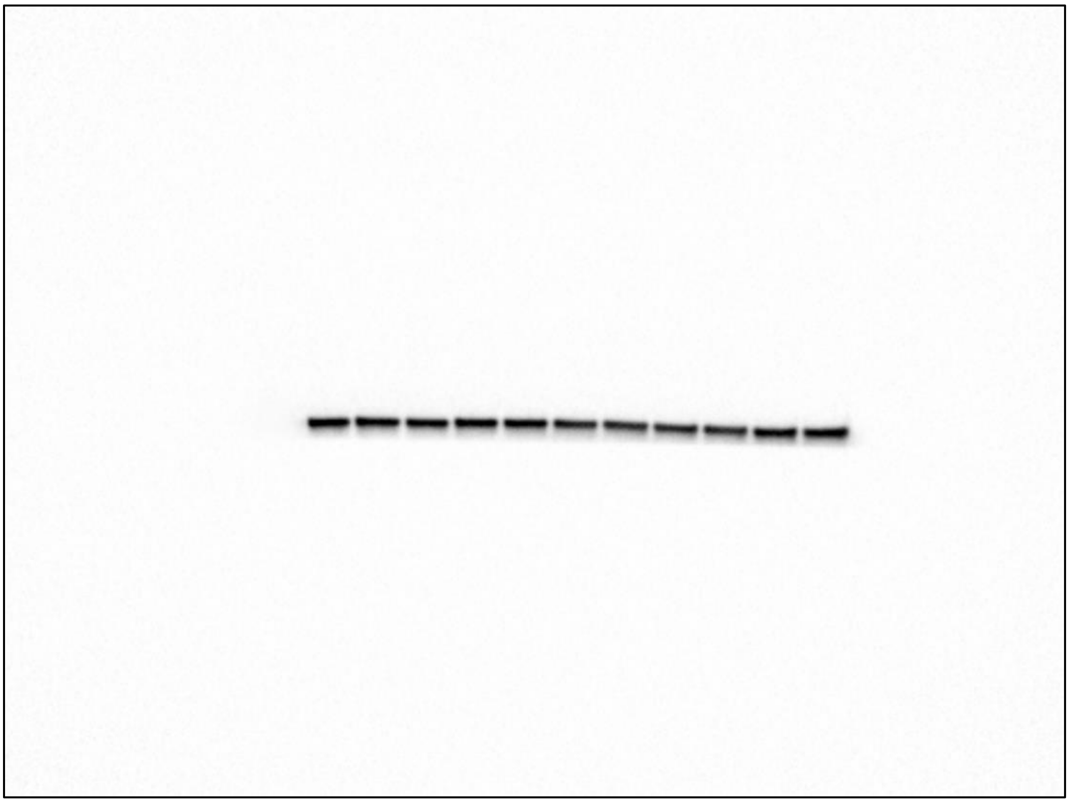

Repeat 2 of β-actin, lanes from left to right: Control, Model, GOS 2.5, 5, 10 mg/ml, FOS 5, 10, 20 mg/ml, COS 2.5, 5, 10 mg/ml.

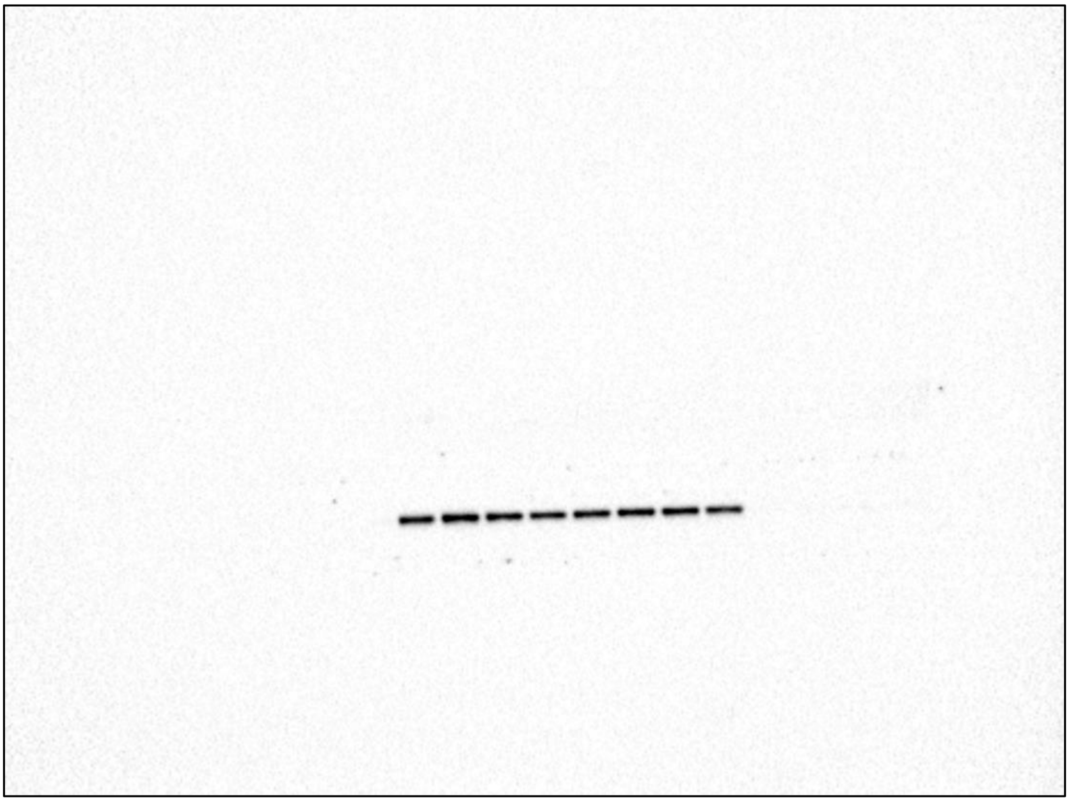

Repeat 2 of β-actin, lanes from left to right: Control, Model, RES 25, 50, 100 μM, ALA 25, 50, 100 μM.

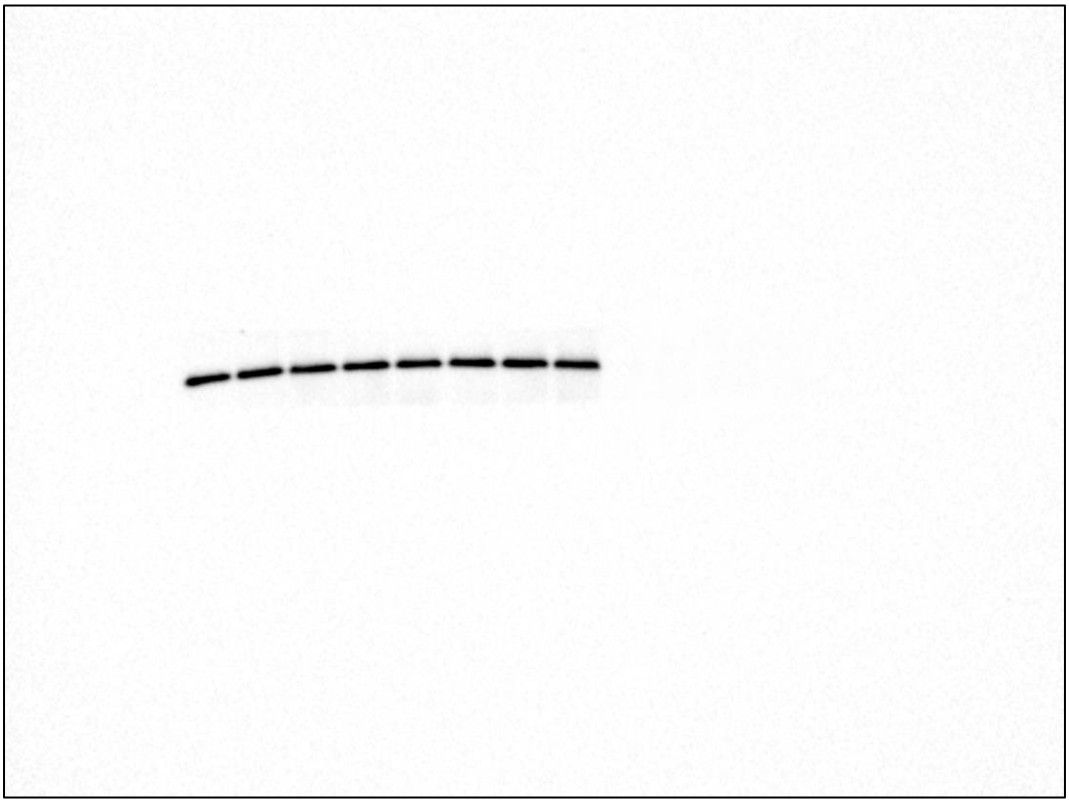

Repeat 2 of β-actin, lanes from left to right: Control, Model, Arg 0.5, 1.0, 2.0 mM, EPA 12.5, 25, 50 μM.

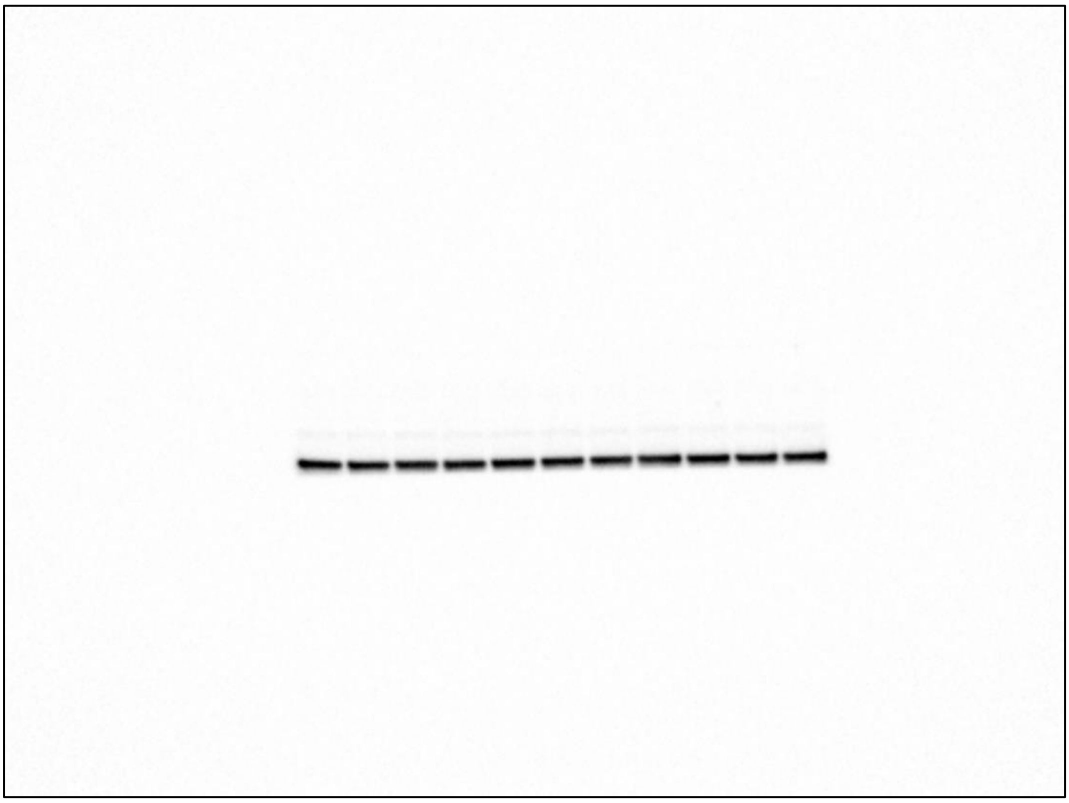

Repeat 3 of β-actin, lanes from left to right: Control, Model, GOS 2.5, 5, 10 mg/ml, FOS 5, 10, 20 mg/ml, COS 2.5, 5, 10 mg/ml.

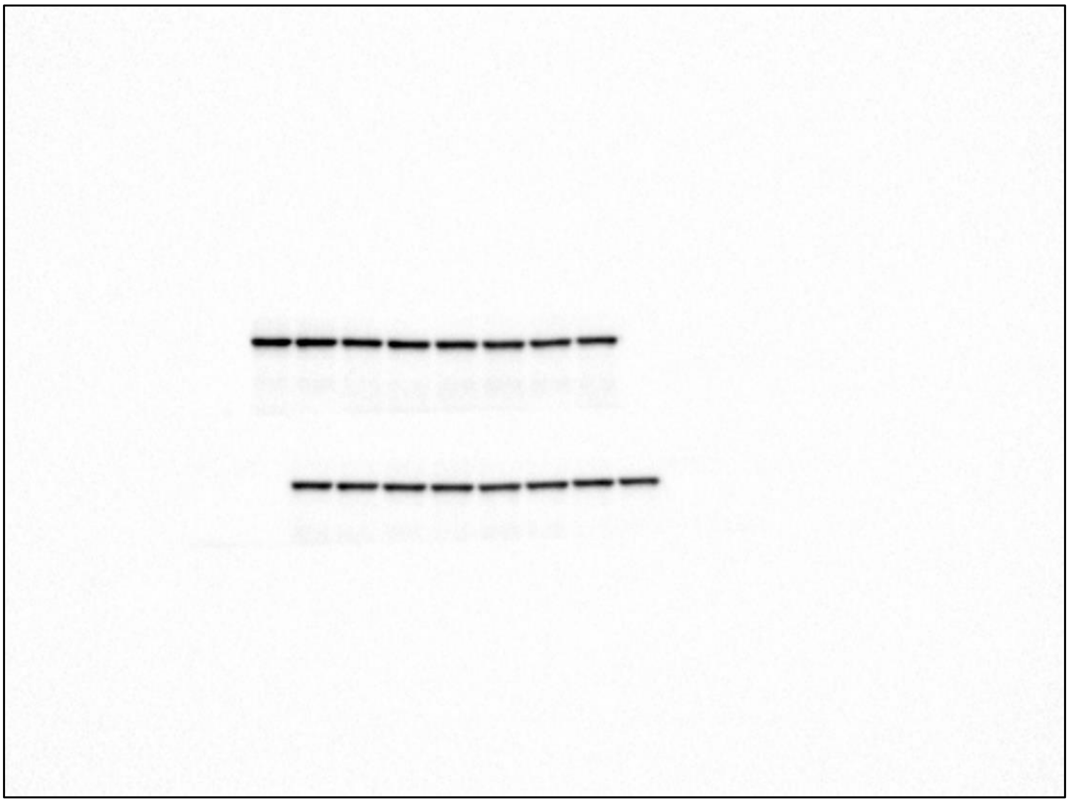

Repeat 3 of β-actin, upper lanes from left to right: Control, Model, RES 25, 50, 100 μM, ALA 25, 50, 100 μM; lower lanes from left to right: Control, Model, Arg 0.5, 1.0, 2.0 mM, EPA 12.5, 25, 50 μM.

Original blot images supporting Figure 5:

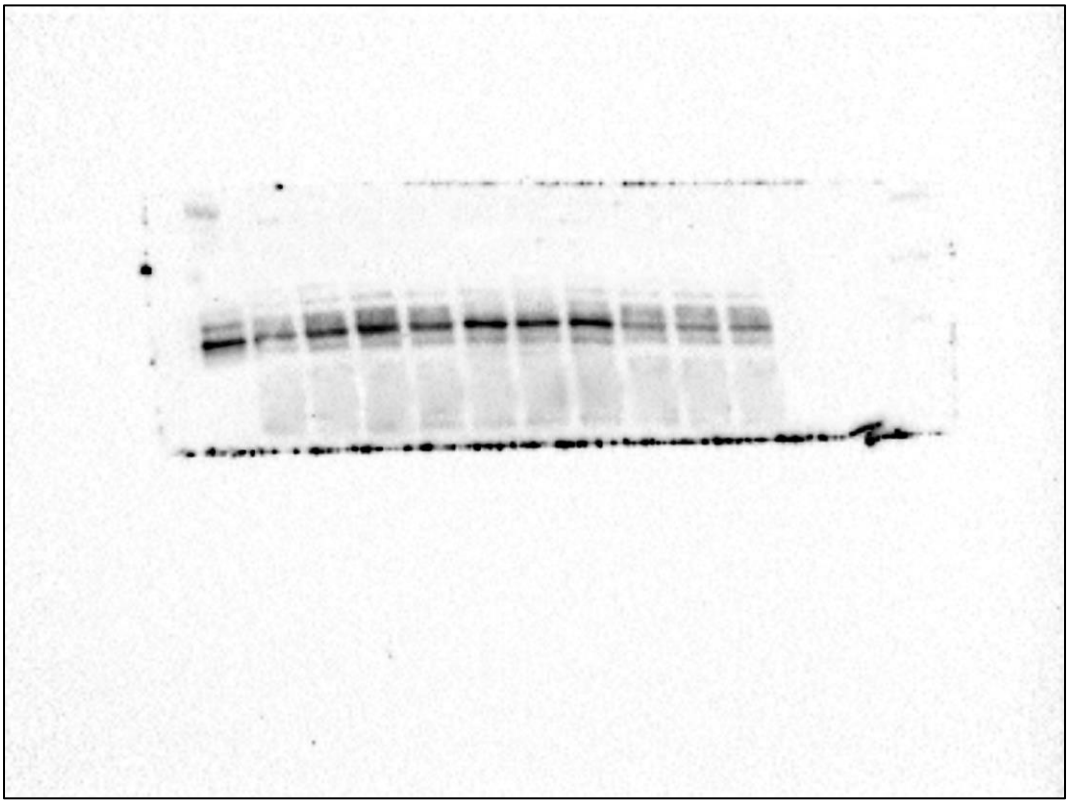

Repeat 1 of CLDN3, lanes from left to right: Control, Model, GOS 2.5, 5, 10 mg/ml, FOS 5, 10, 20 mg/ml, COS 2.5, 5, 10 mg/ml.

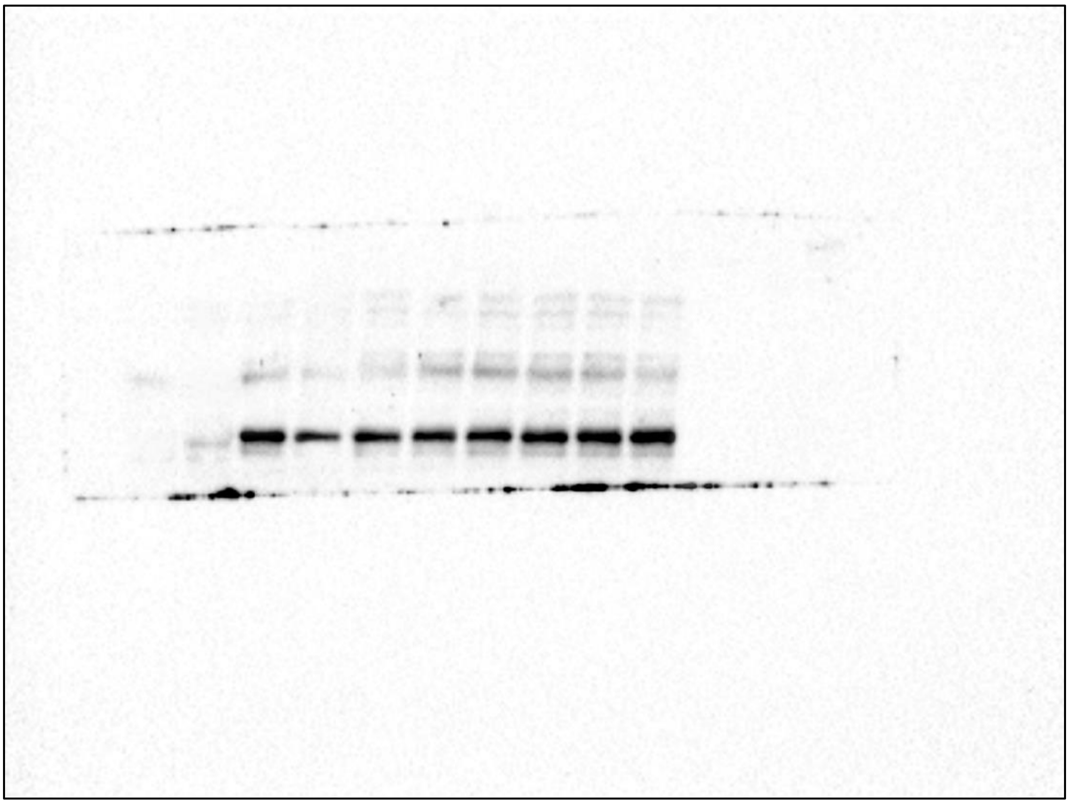

Repeat 1 of CLDN3, lanes from left to right: Control, Model, RES 25, 50, 100 µM, ALA 25, 50, 100 µM.

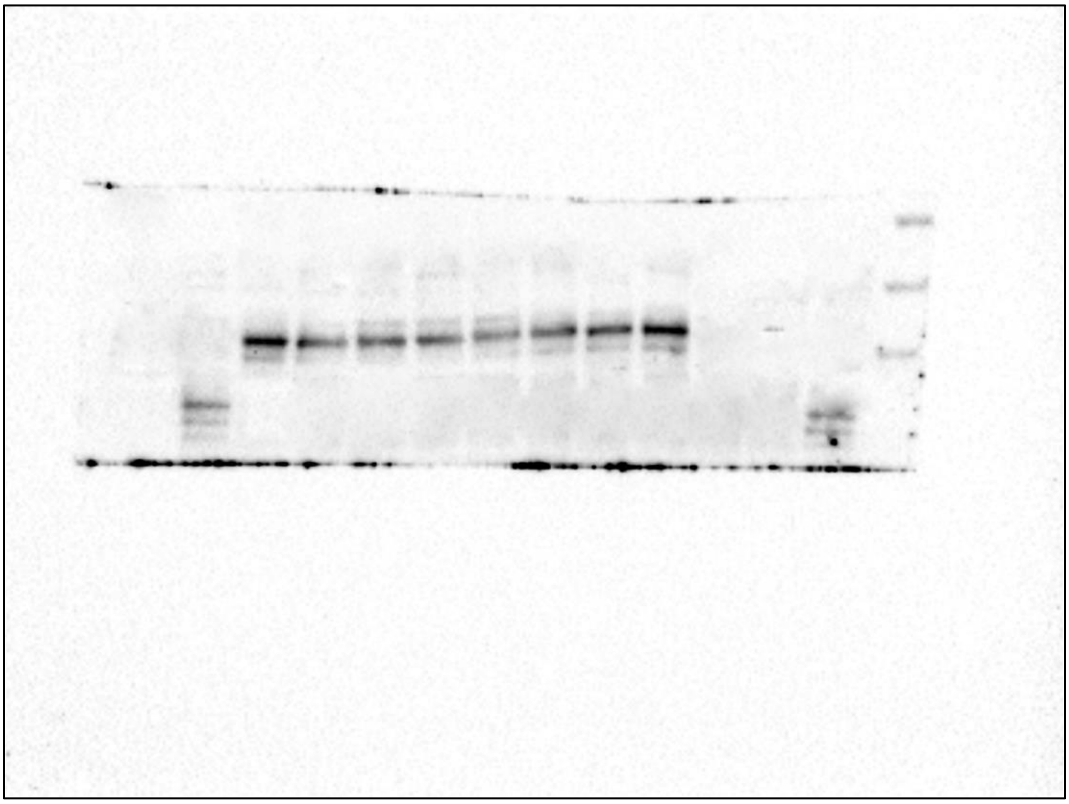

Repeat 1 of CLDN3, lanes from left to right: Control, Model, Arg 0.5, 1.0, 2.0 mM, EPA 12.5, 25, 50 µM.

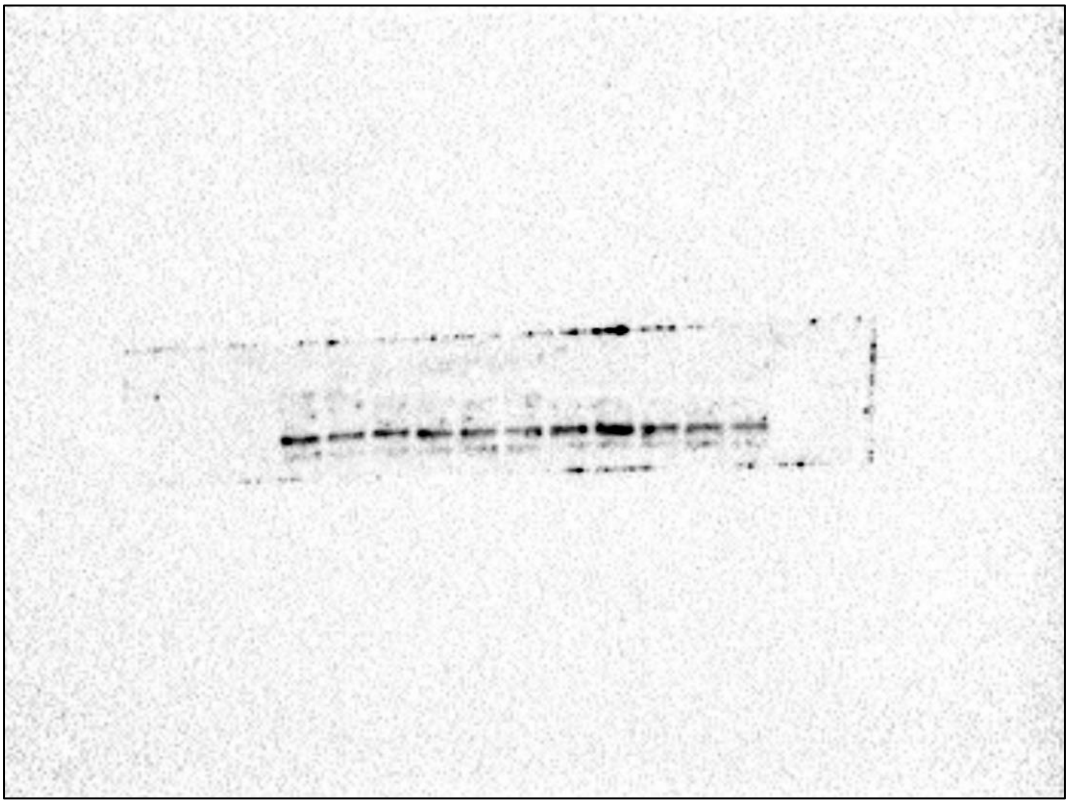

Repeat 2 of CLDN3, lanes from left to right: Control, Model, GOS 2.5, 5, 10 mg/ml, FOS 5, 10, 20 mg/ml, COS 2.5, 5, 10 mg/ml.

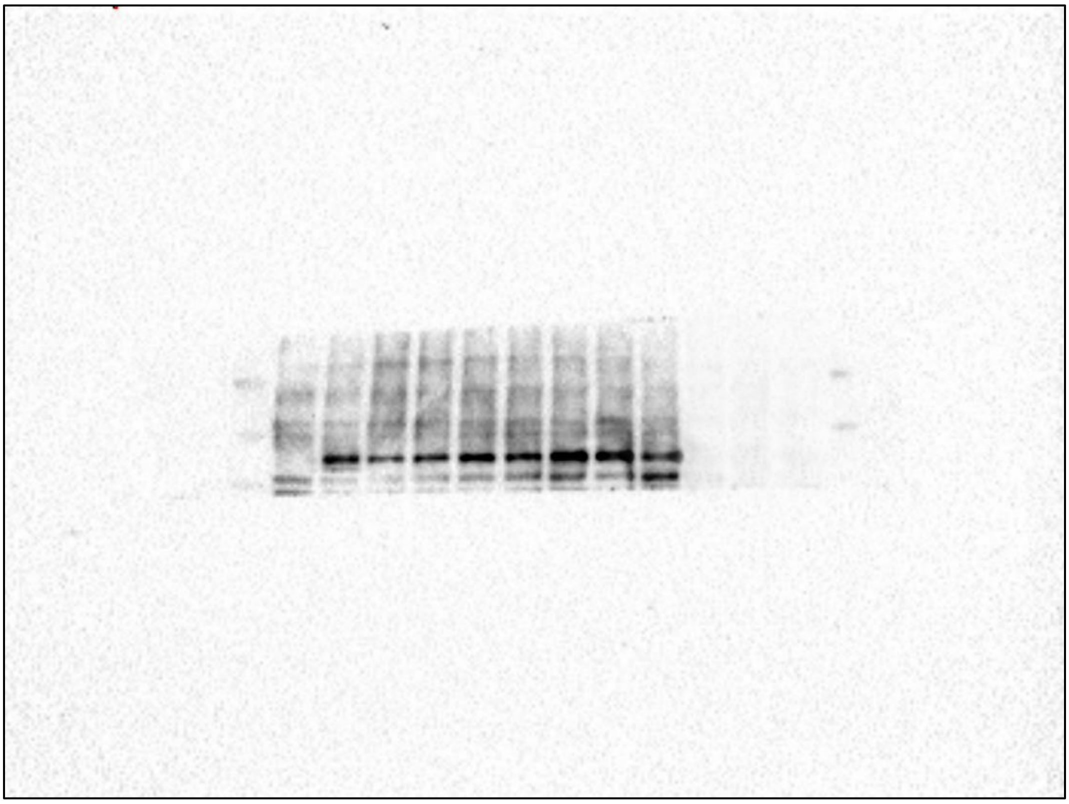

Repeat 2 of CLDN3, lanes from left to right: Control, Model, RES 25, 50, 100 µM, ALA 25, 50, 100 µM.

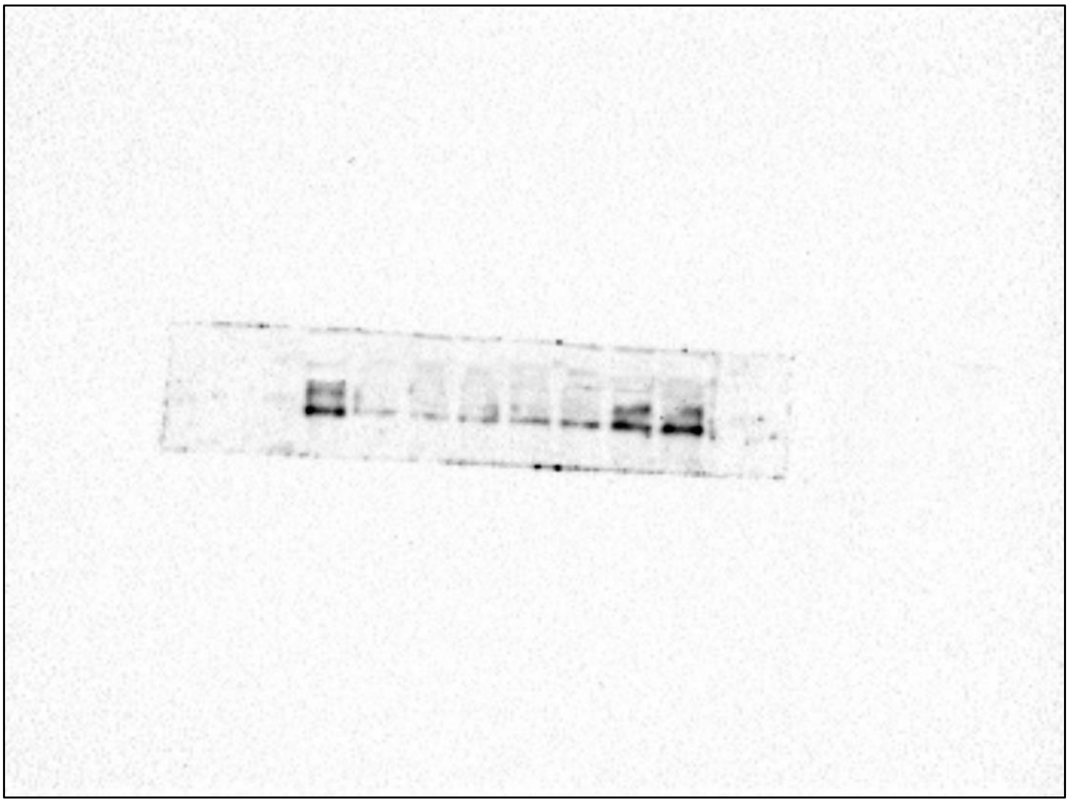

Repeat 2 of CLDN3, lanes from left to right: Control, Model, Arg 0.5, 1.0, 2.0 mM, EPA 12.5, 25, 50 μM.

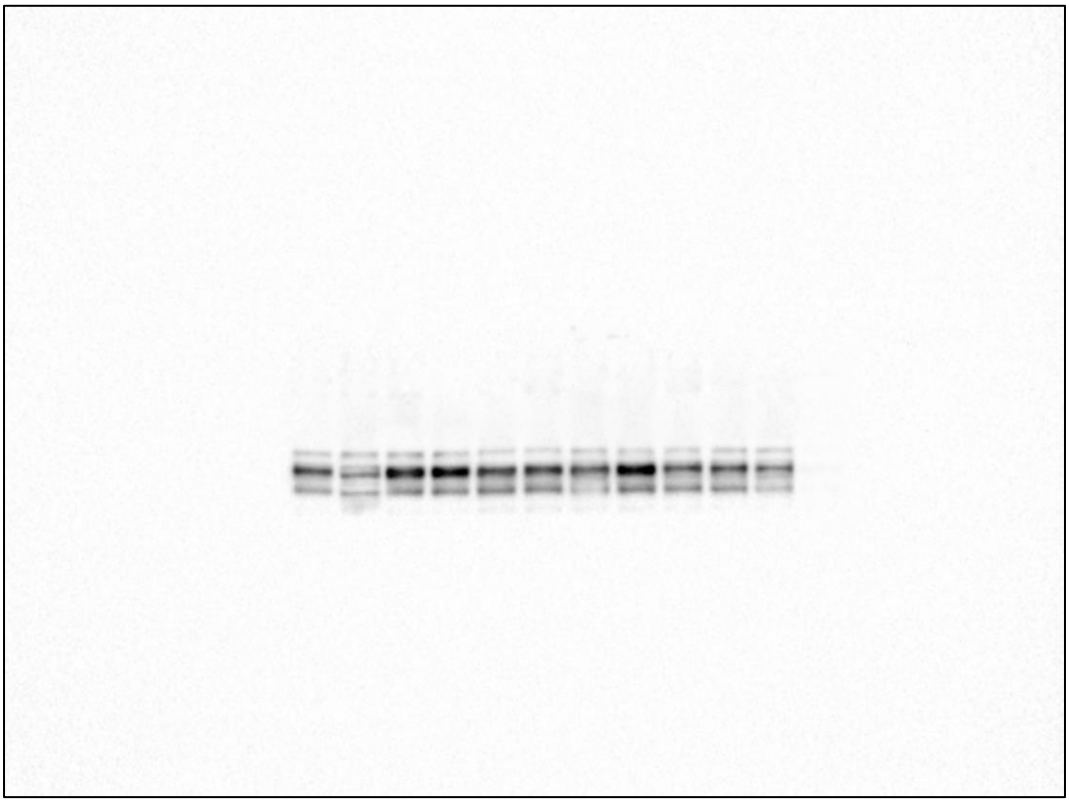

Repeat 3 of CLDN3, lanes from left to right: Control, Model, GOS 2.5, 5, 10 mg/ml, FOS 5, 10, 20 mg/ml, COS 2.5, 5, 10 mg/ml.

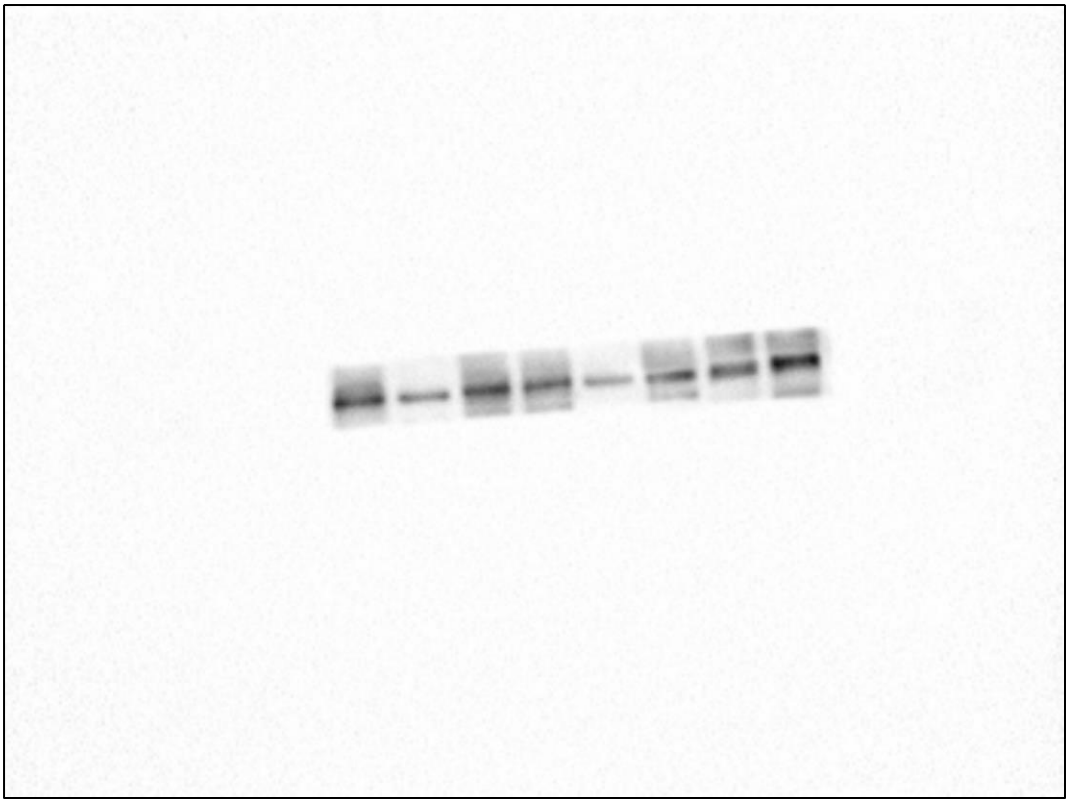

Repeat 3 of CLDN3, lanes from left to right: Control, Model, RES 25, 50, 100 µM, ALA 25, 50, 100 µM.

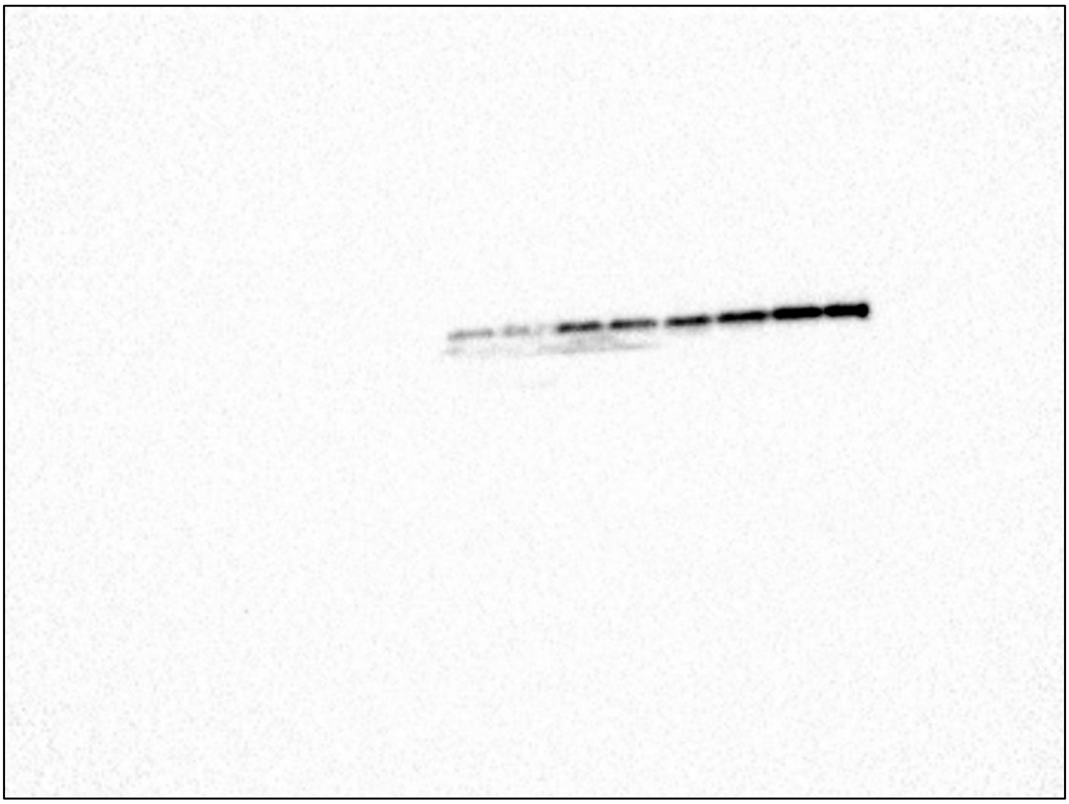

Repeat 3 of CLDN3, lanes from left to right: Control, Model, Arg 0.5, 1.0, 2.0 mM, EPA 12.5, 25, 50 µM.

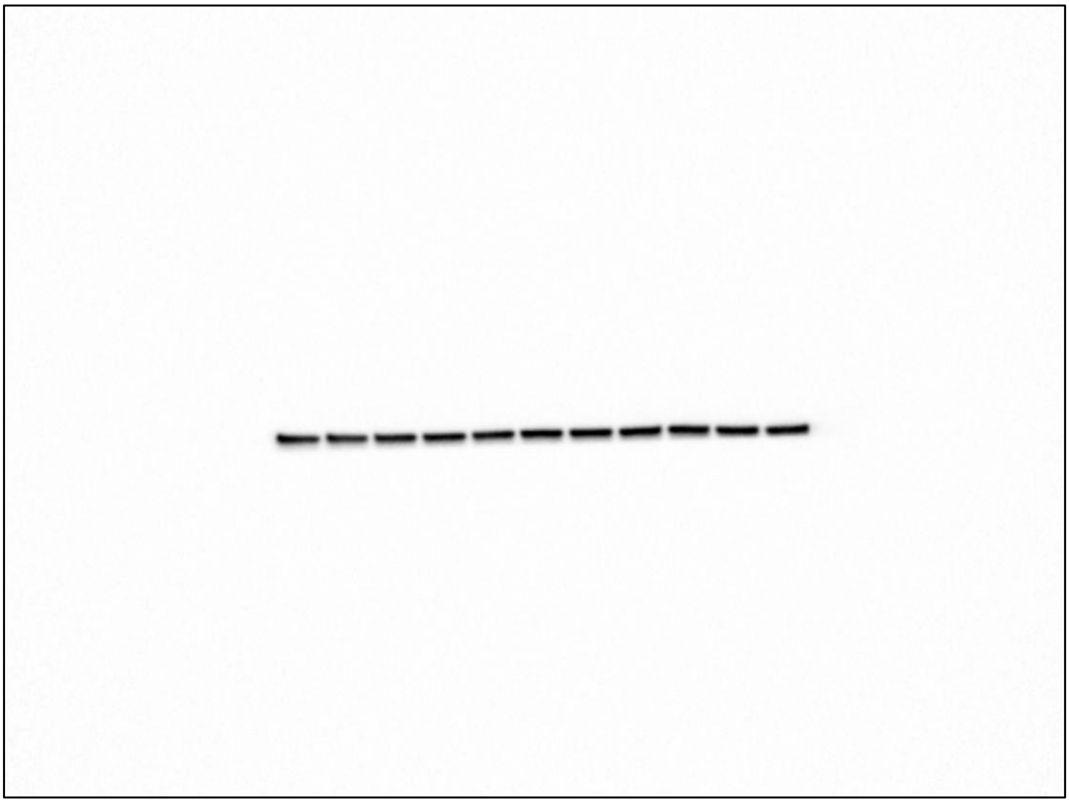

Repeat 1 of β-actin, lanes from left to right: Control, Model, GOS 2.5, 5, 10 mg/ml, FOS 5, 10, 20 mg/ml, COS 2.5, 5, 10 mg/ml.

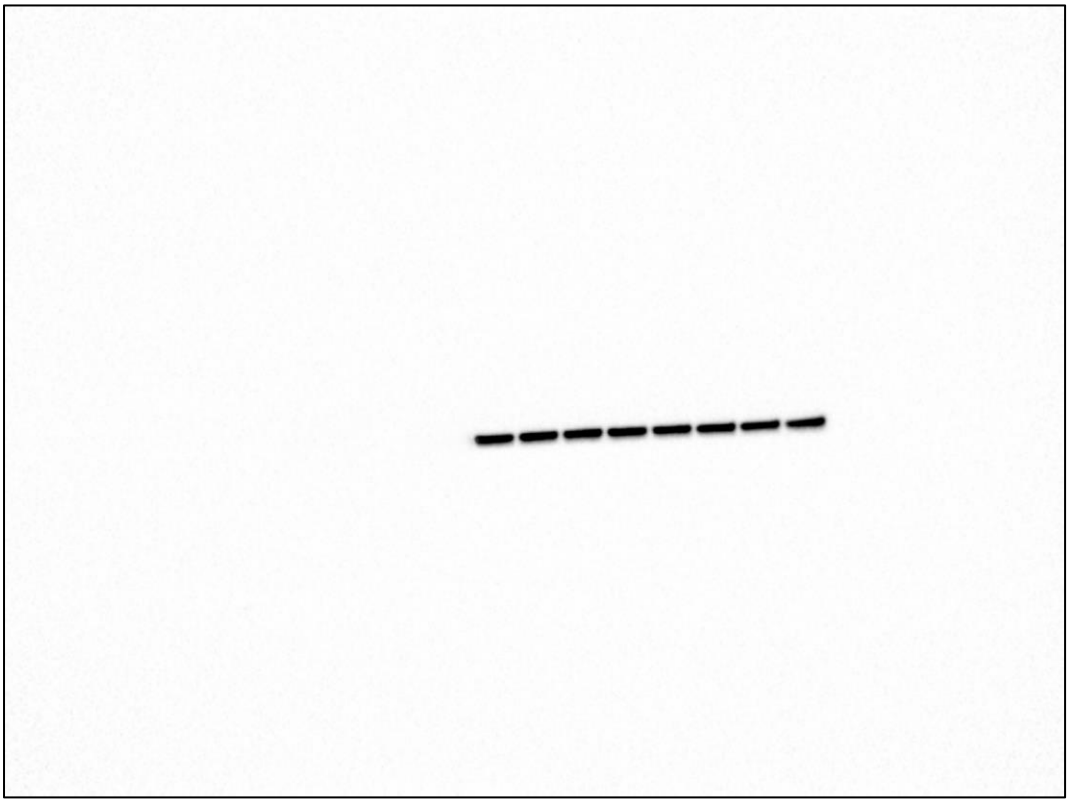

Repeat 1 of β-actin, lanes from left to right: Control, Model, RES 25, 50, 100 μM, ALA 25, 50, 100 μM.

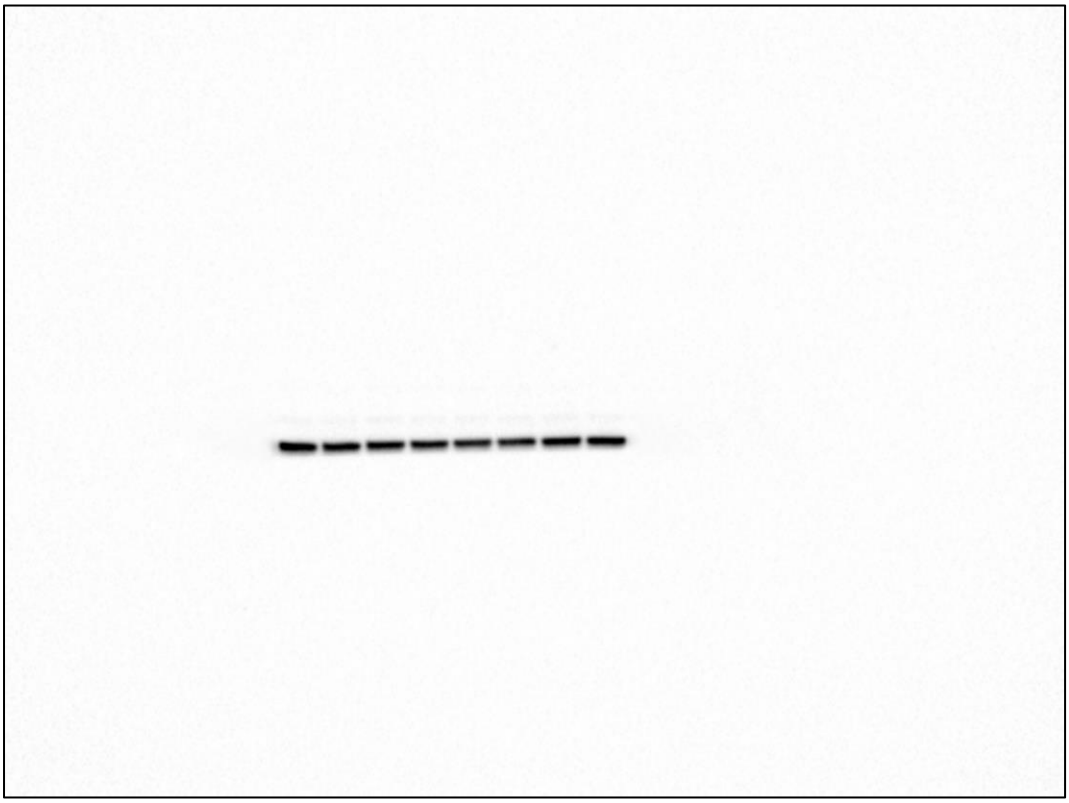

Repeat 1 of β-actin, lanes from left to right: Control, Model, Arg 0.5, 1.0, 2.0 mM, EPA 12.5, 25, 50 µM.

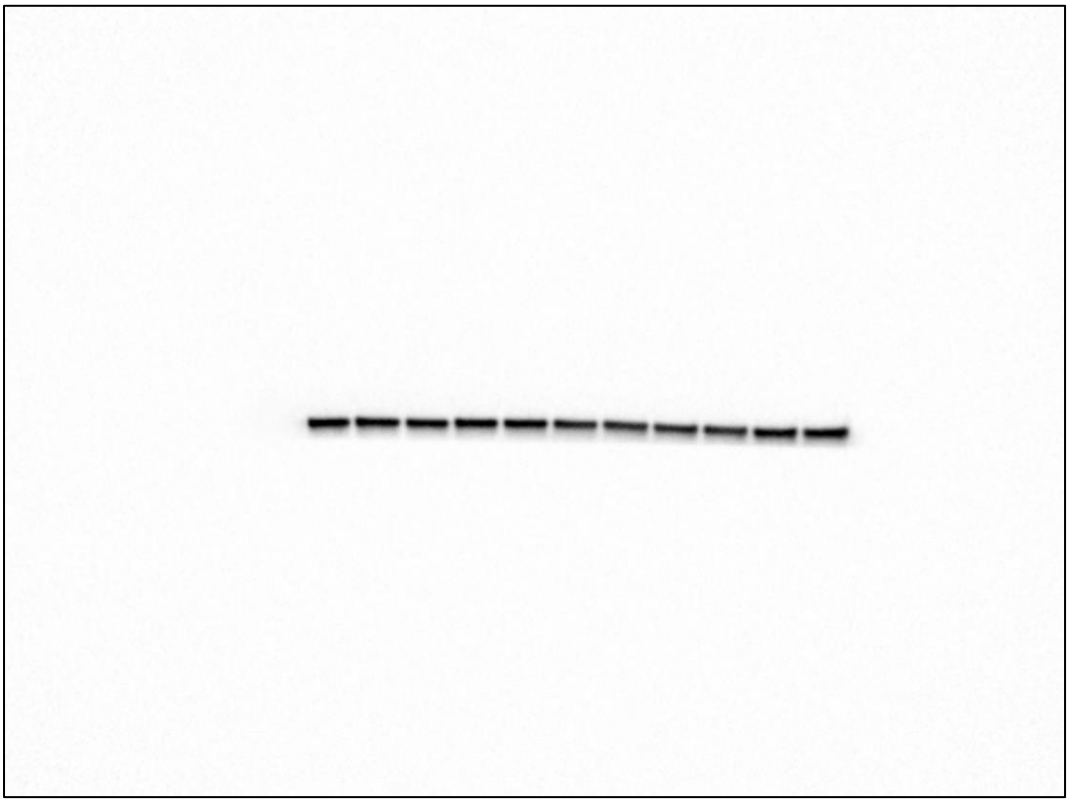

Repeat 2 of β-actin, lanes from left to right: Control, Model, GOS 2.5, 5, 10 mg/ml, FOS 5, 10, 20 mg/ml, COS 2.5, 5, 10 mg/ml.

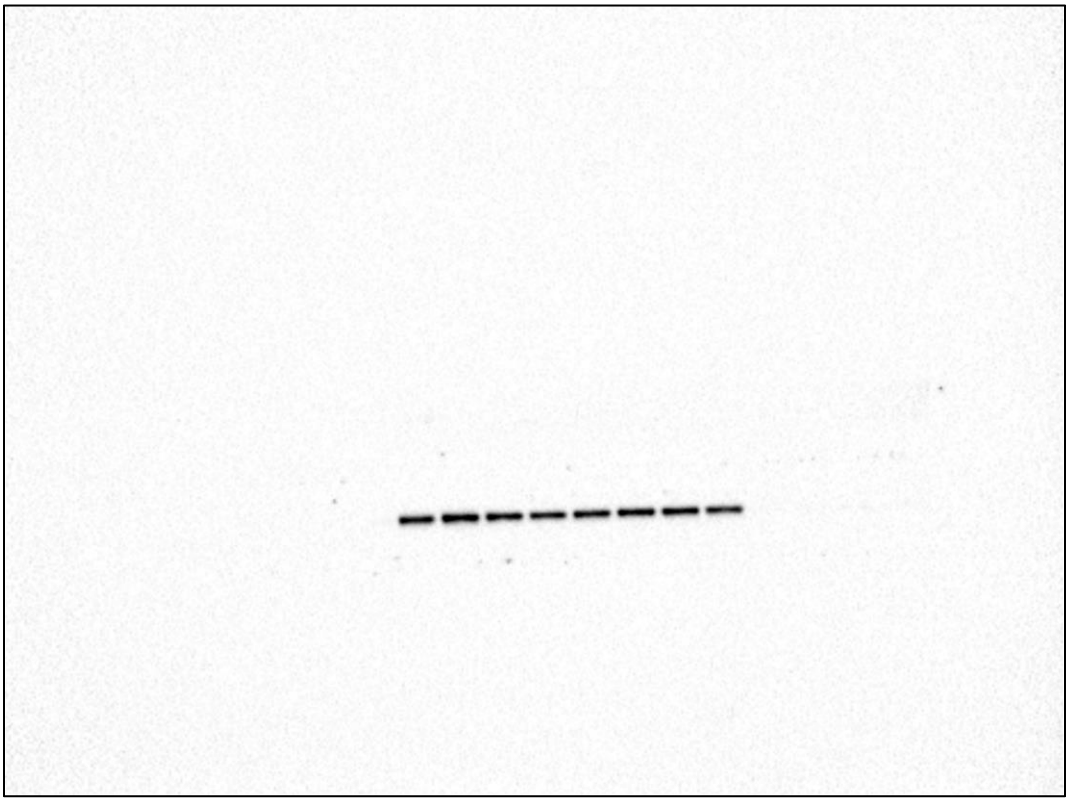

Repeat 2 of β-actin, lanes from left to right: Control, Model, RES 25, 50, 100 µM, ALA 25, 50, 100 µM.

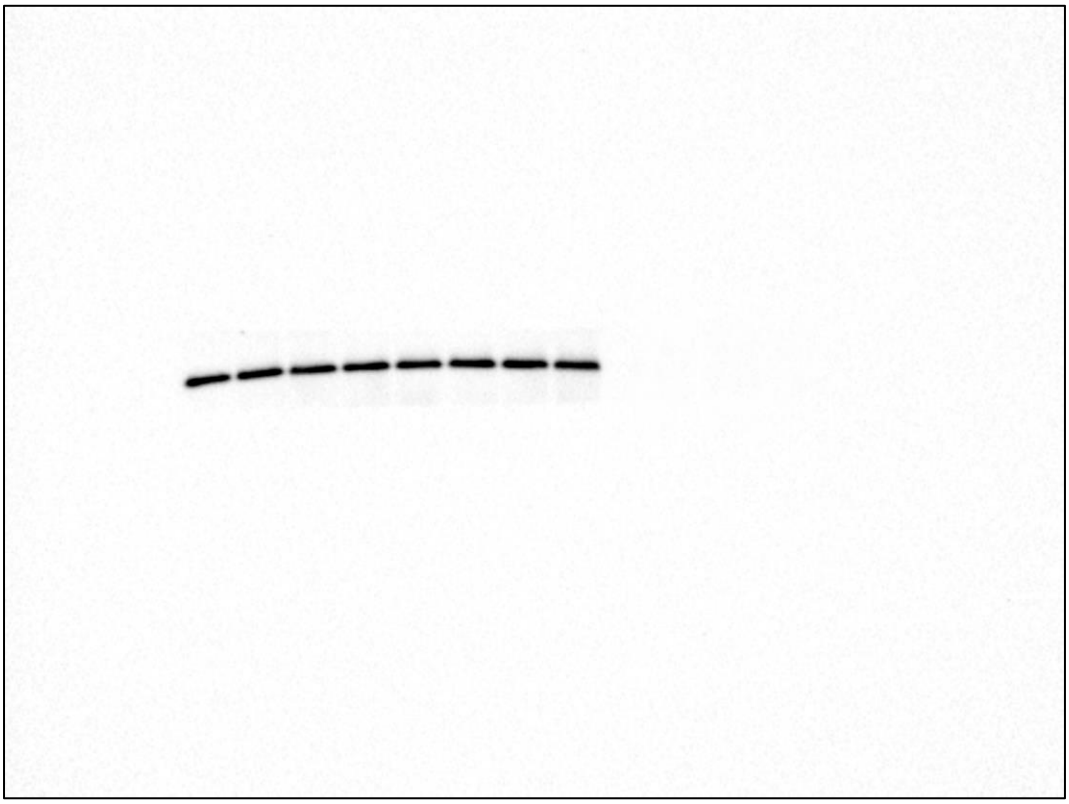

Repeat 2 of β-actin, lanes from left to right: Control, Model, Arg 0.5, 1.0, 2.0 mM, EPA 12.5, 25, 50 µM.

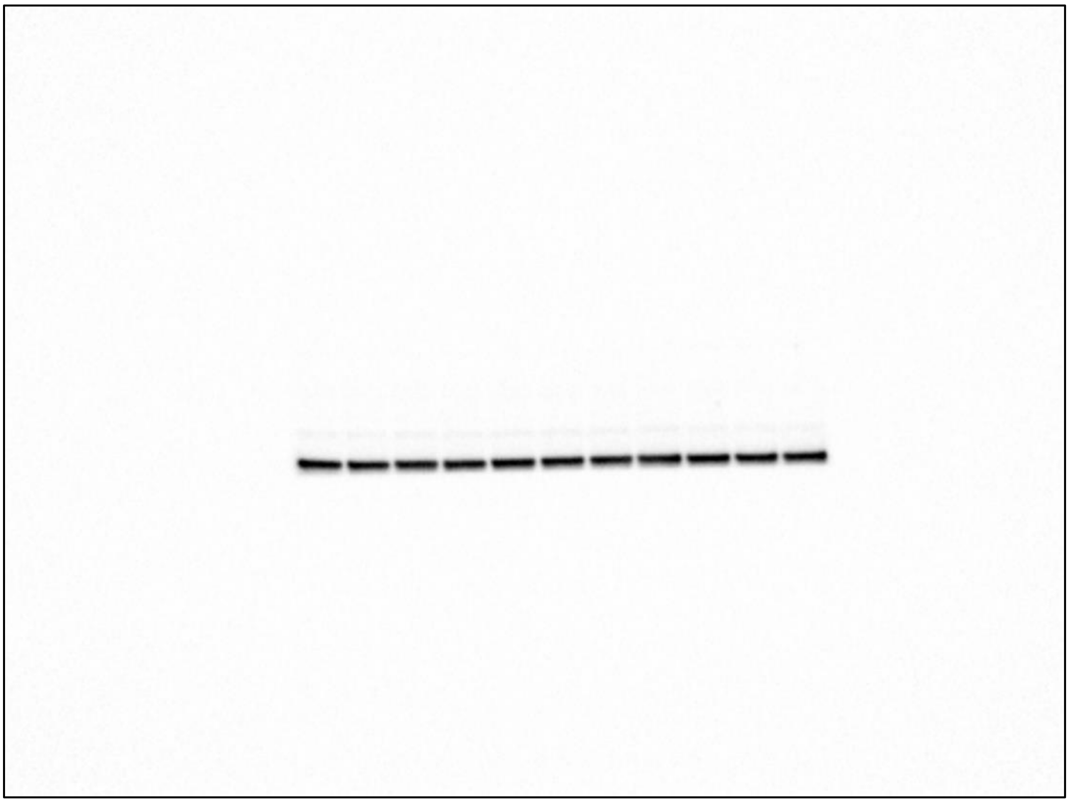

Repeat 3 of β-actin, lanes from left to right: Control, Model, GOS 2.5, 5, 10 mg/ml, FOS 5, 10, 20 mg/ml, COS 2.5, 5, 10 mg/ml.

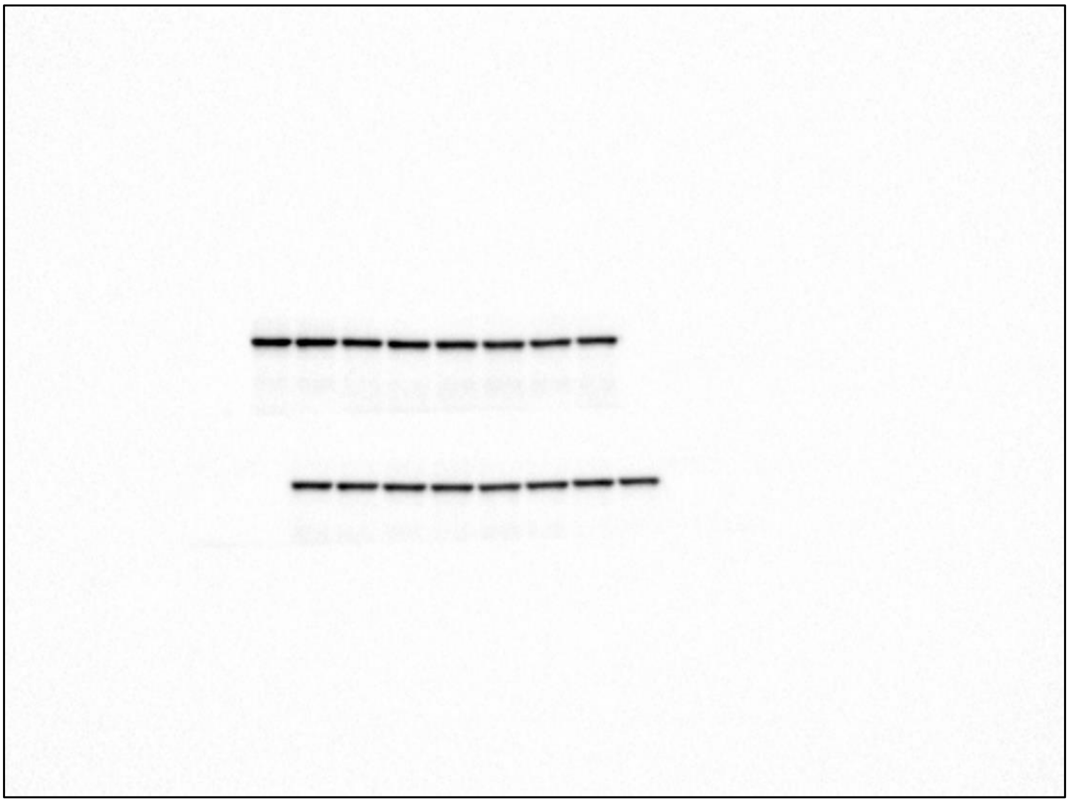

Repeat 3 of β-actin, upper lanes from left to right: Control, Model, RES 25, 50, 100 μM, ALA 25, 50, 100 μM; lower lanes from left to right: Control, Model, Arg 0.5, 1.0, 2.0 mM, EPA 12.5, 25, 50 μM.

Original blot images supporting Figure 7:

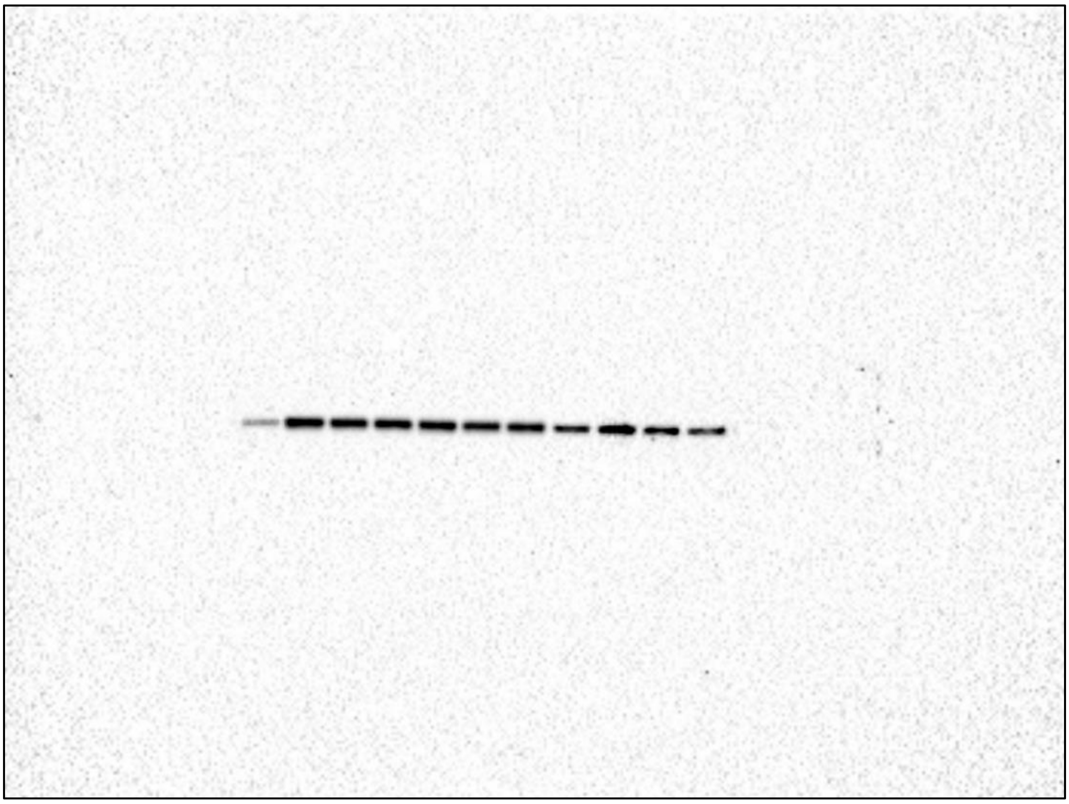

Repeat 1 of HSP-70, lanes from left to right: Control, Model, GOS 2.5, 5, 10 mg/ml, FOS 5, 10, 20 mg/ml, COS 2.5, 5, 10 mg/ml.

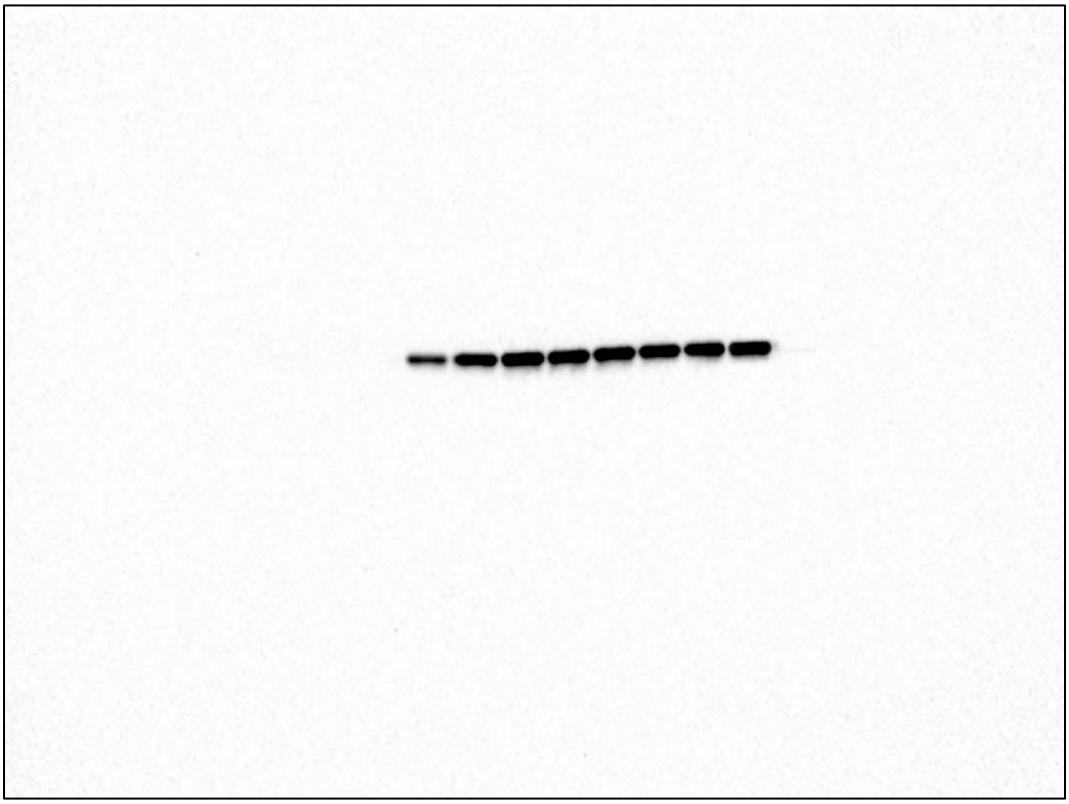

Repeat 1 of HSP-70, lanes from left to right: Control, Model, RES 25, 50, 100 µM, ALA 25, 50, 100 µM.

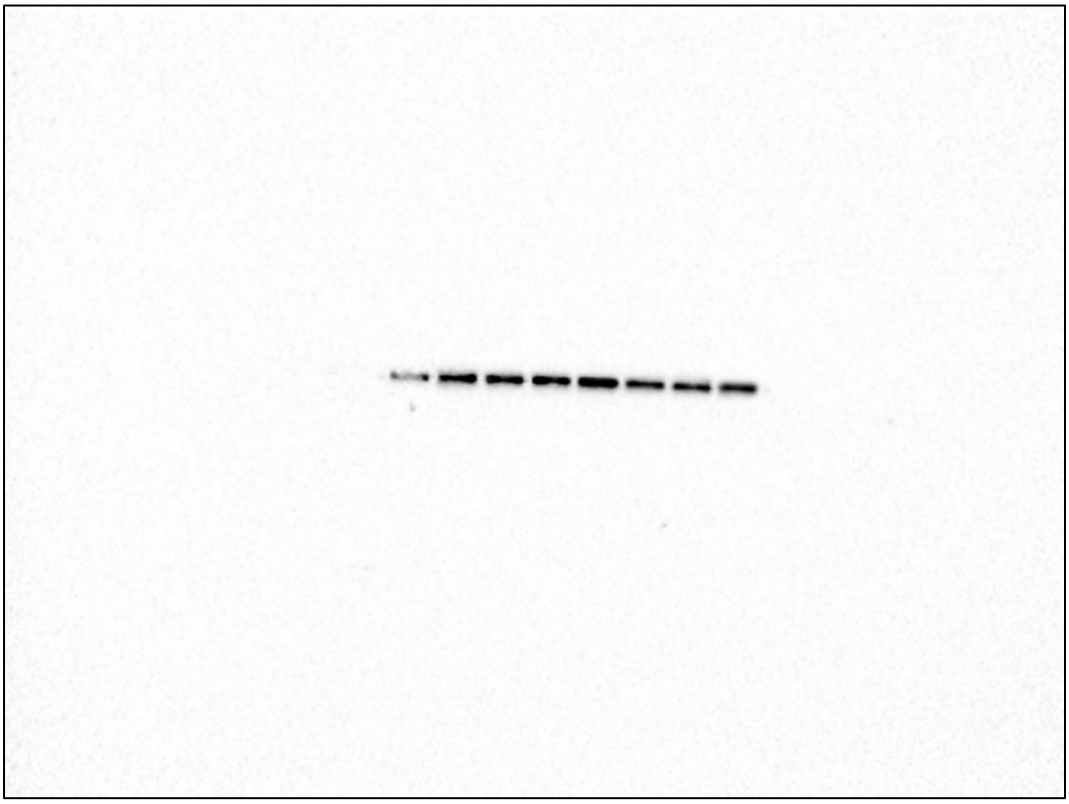

Repeat 1 of HSP-70, lanes from left to right: Control, Model, Arg 0.5, 1.0, 2.0 mM, EPA 12.5, 25, 50 µM.

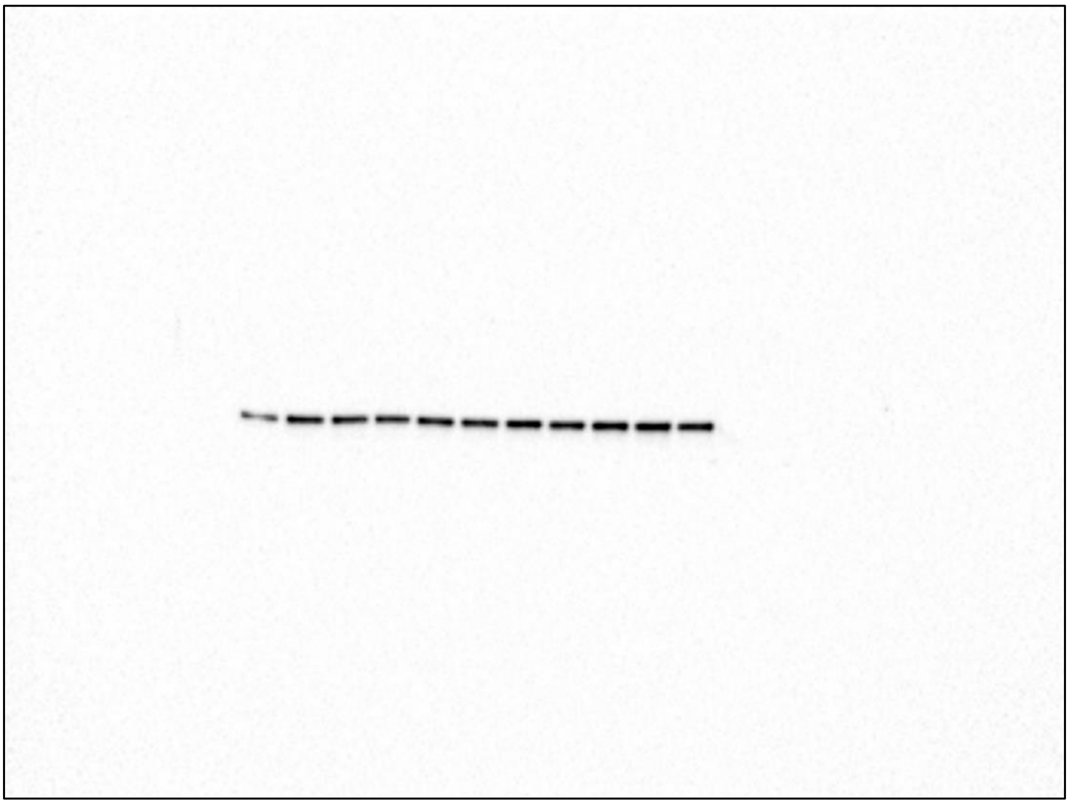

Repeat 2 of HSP-70, lanes from left to right: Control, Model, GOS 2.5, 5, 10 mg/ml, FOS 5, 10, 20 mg/ml, COS 2.5, 5, 10 mg/ml.

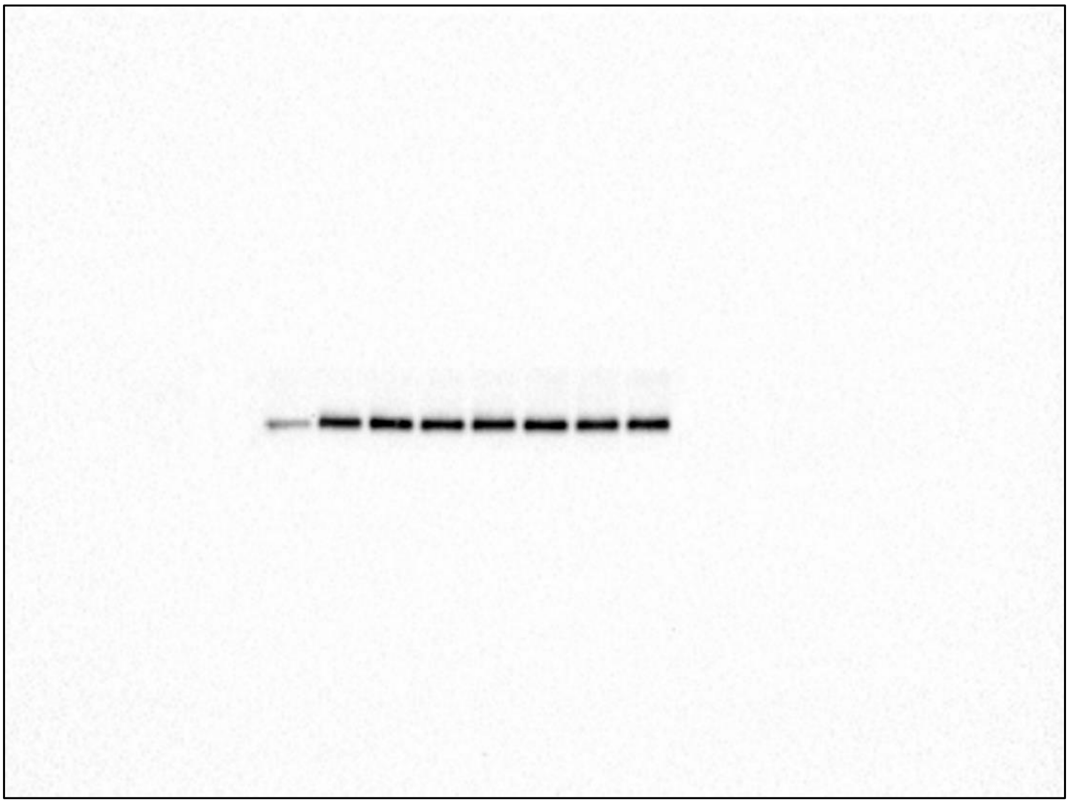

Repeat 2 of HSP-70, lanes from left to right: Control, Model, RES 25, 50, 100 μM, ALA 25, 50, 100 μM.

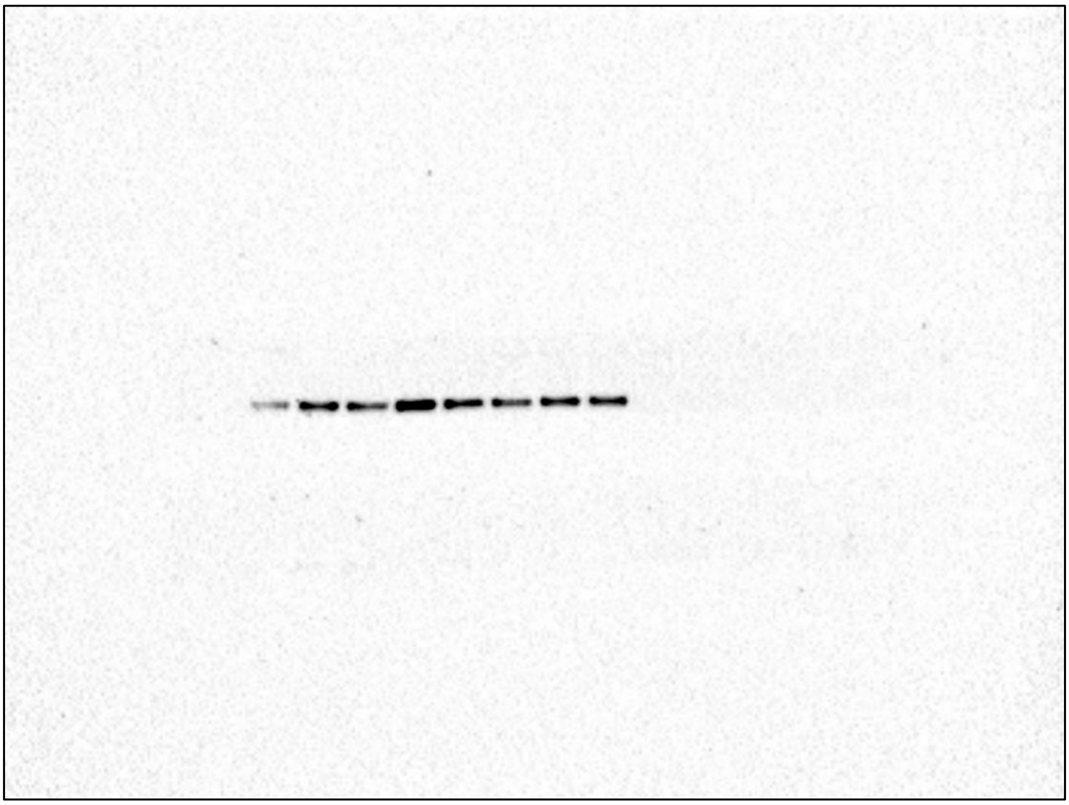

Repeat 2 of HSP-70, lanes from left to right: Control, Model, Arg 0.5, 1.0, 2.0 mM, EPA 12.5, 25, 50 μM.

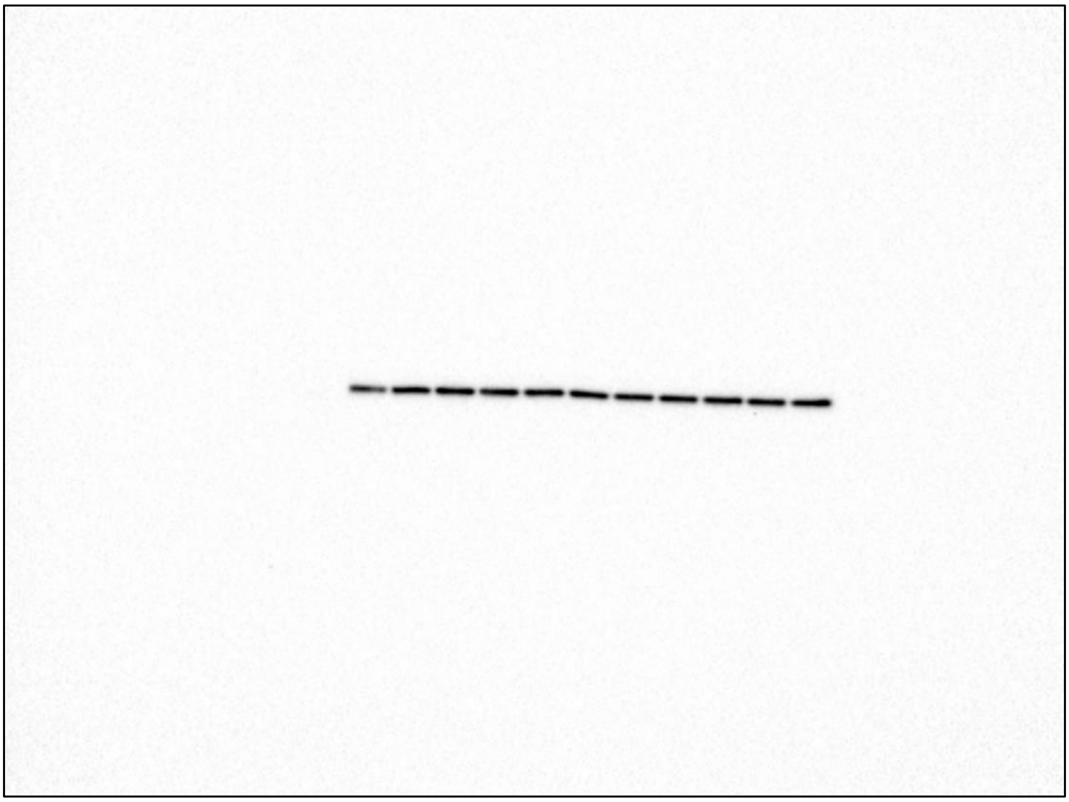

Repeat 3 of HSP-70, lanes from left to right: Control, Model, GOS 2.5, 5, 10 mg/ml, FOS 5, 10, 20 mg/ml, COS 2.5, 5, 10 mg/ml.

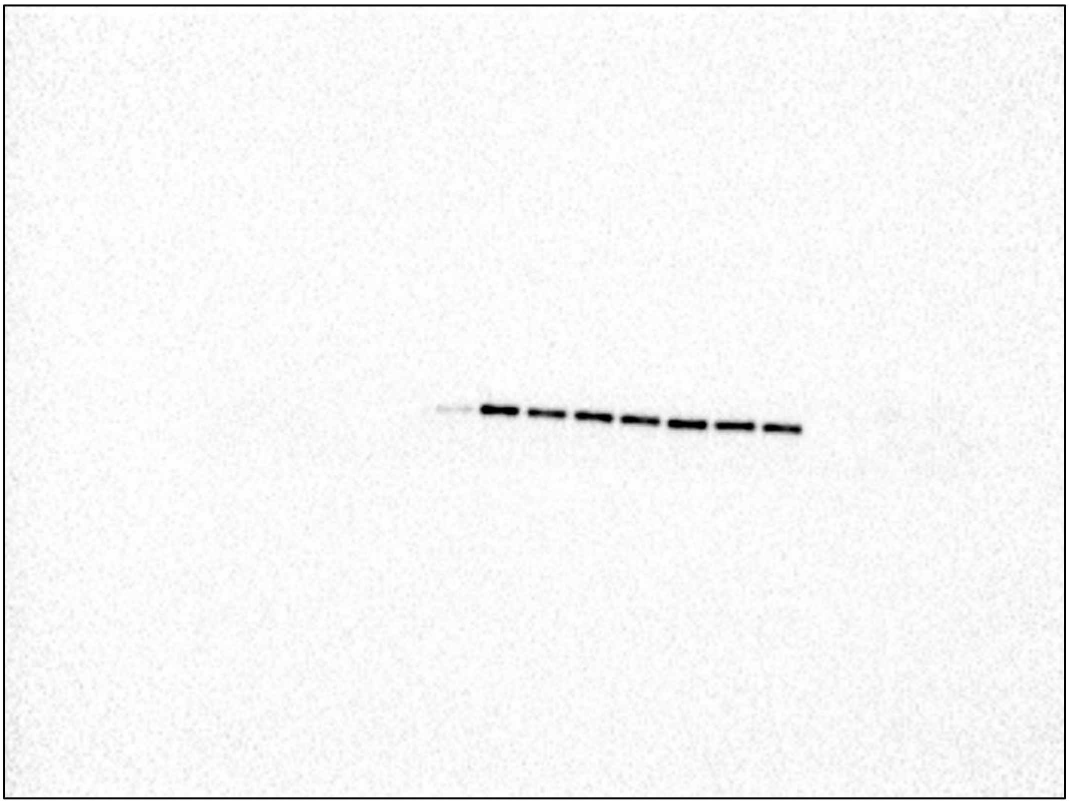

Repeat 3 of HSP-70, lanes from left to right: Control, Model, RES 25, 50, 100 μM, ALA 25, 50, 100 μM.

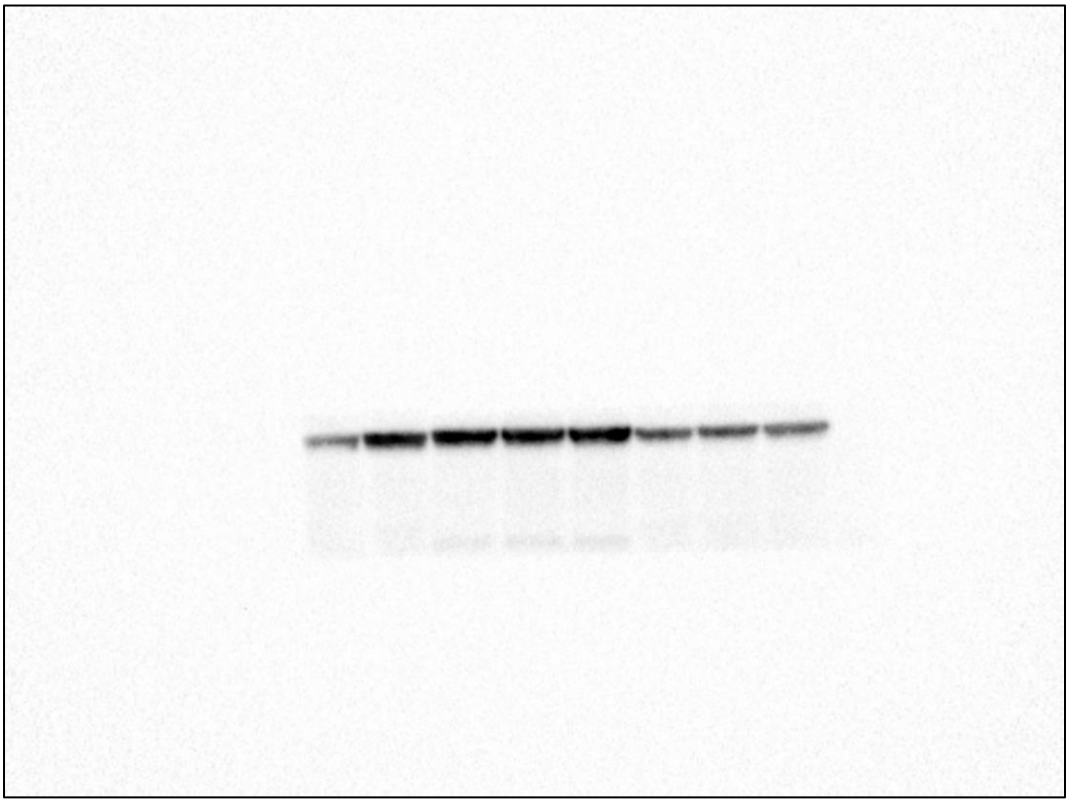

Repeat 3 of HSP-70, lanes from left to right: Control, Model, Arg 0.5, 1.0, 2.0 mM, EPA 12.5, 25, 50 μM.

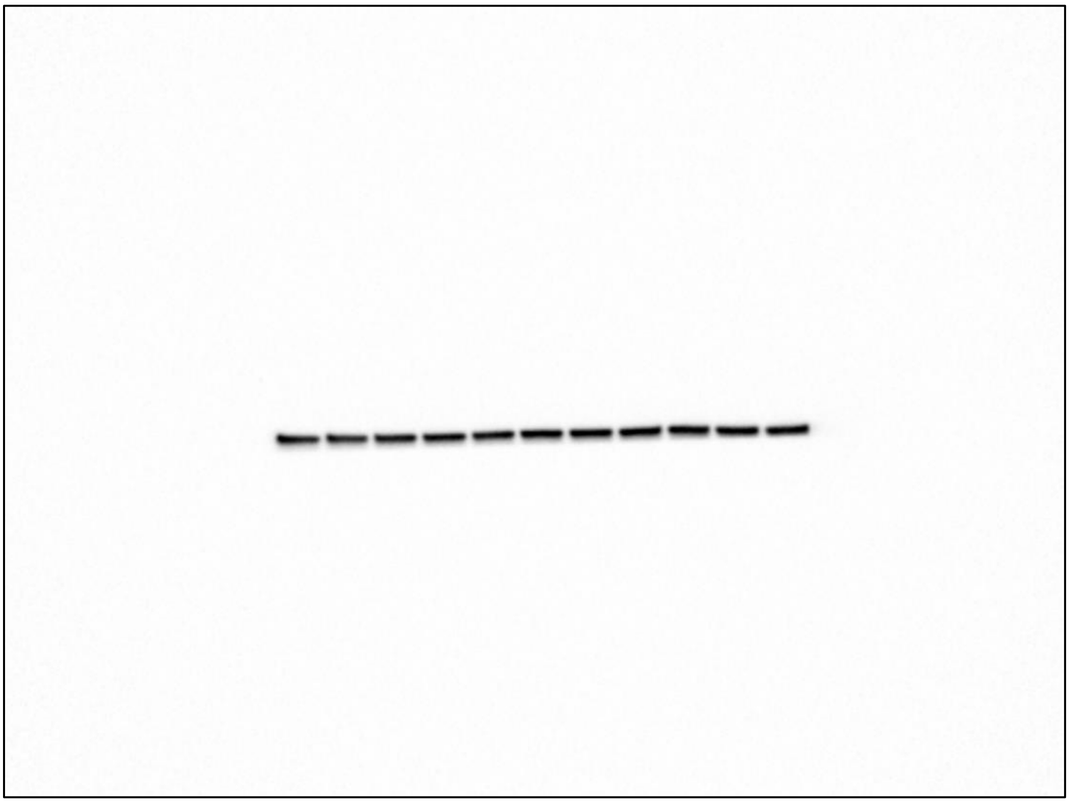

Repeat 1 of β-actin, lanes from left to right: Control, Model, GOS 2.5, 5, 10 mg/ml, FOS 5, 10, 20 mg/ml, COS 2.5, 5, 10 mg/ml.

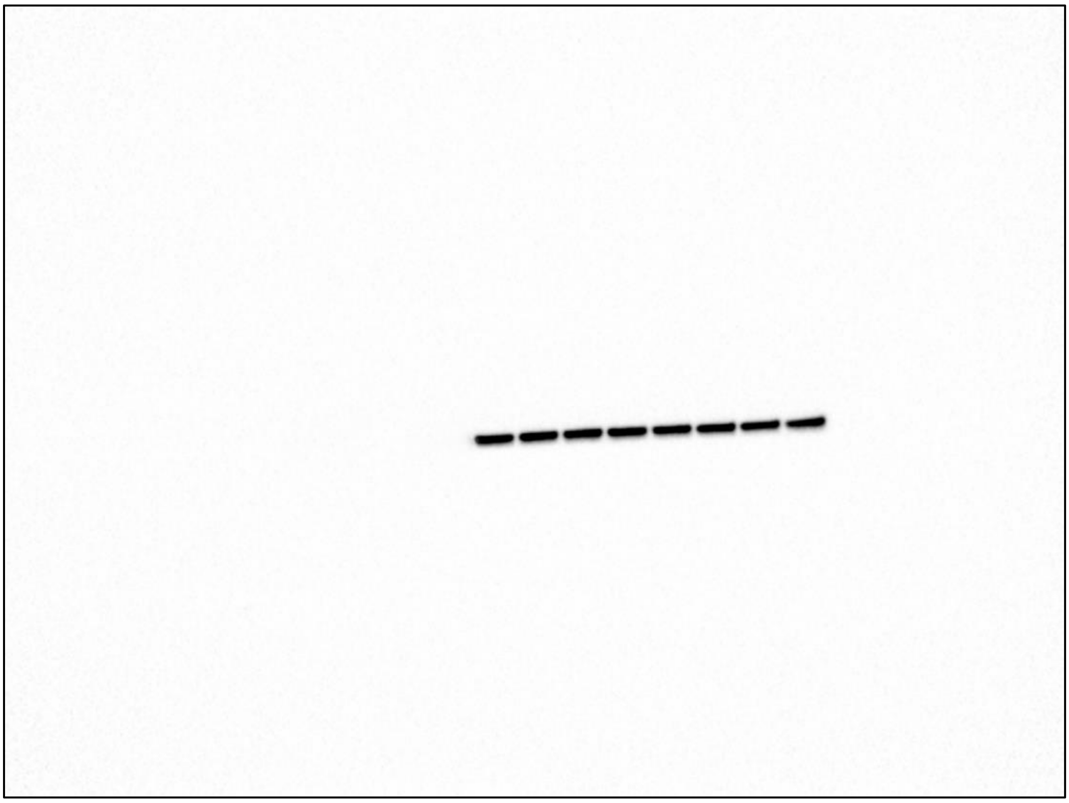

Repeat 1 of β-actin, lanes from left to right: Control, Model, RES 25, 50, 100 μM, ALA 25, 50, 100 μM.

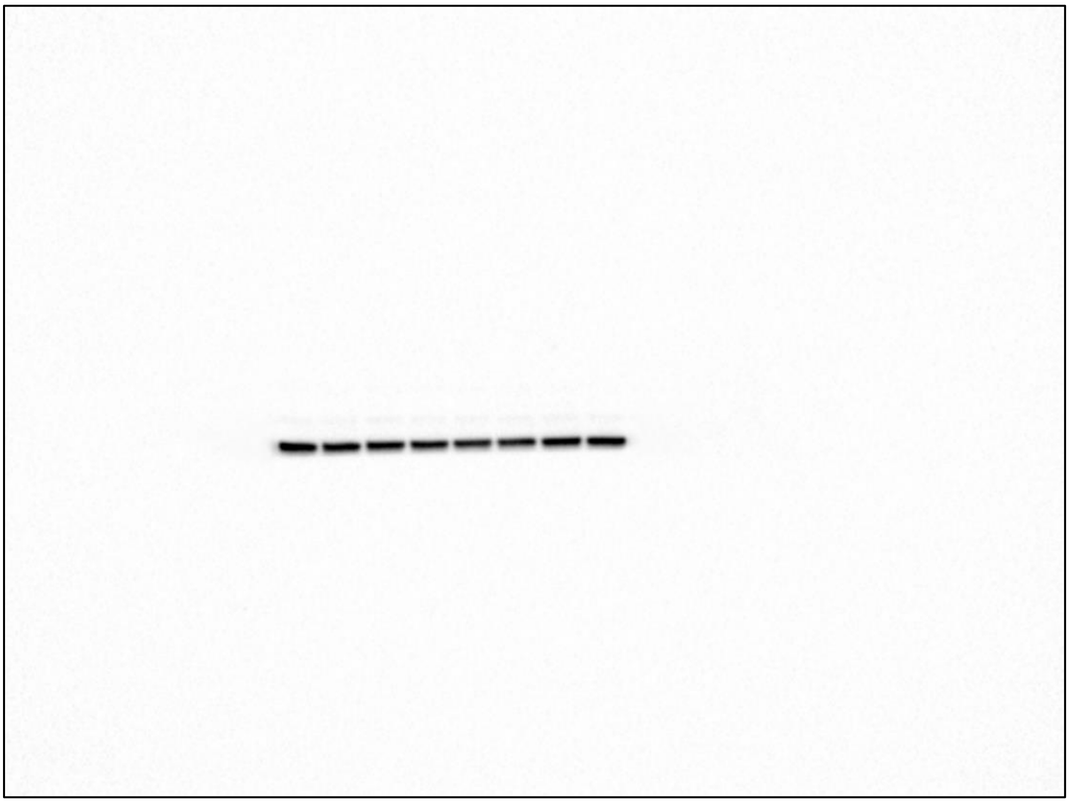

Repeat 1 of β-actin, lanes from left to right: Control, Model, Arg 0.5, 1.0, 2.0 mM, EPA 12.5, 25, 50 μM.

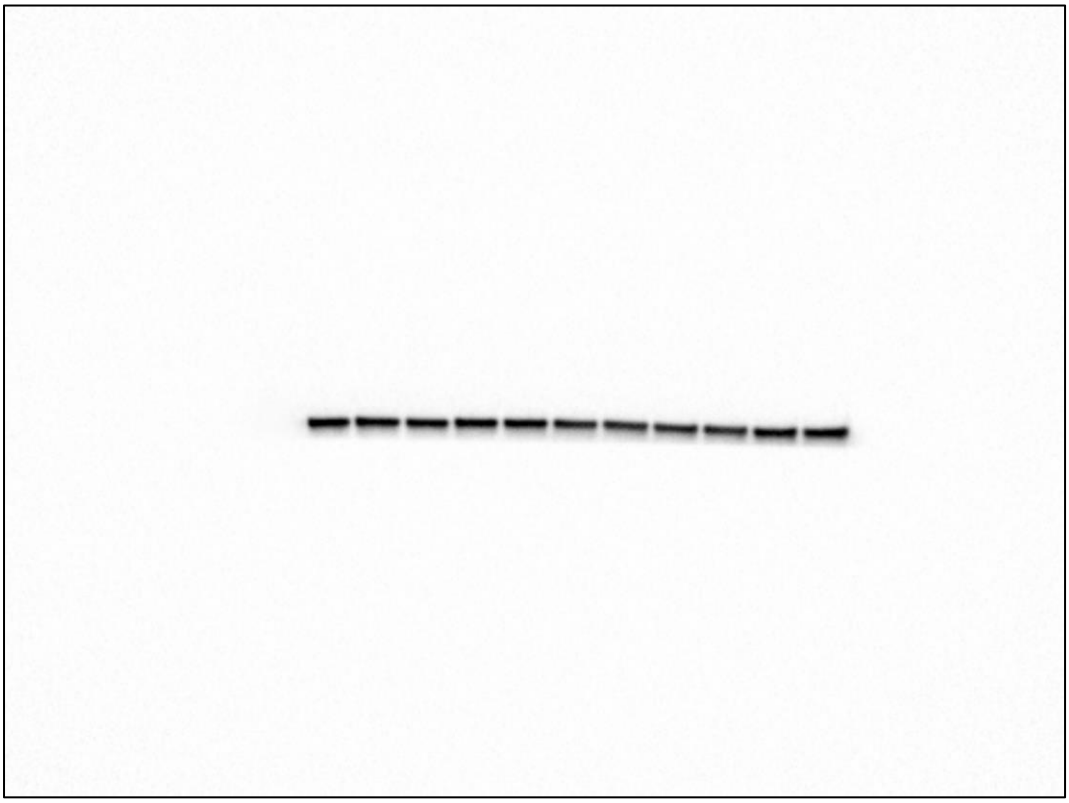

Repeat 2 of β-actin, lanes from left to right: Control, Model, GOS 2.5, 5, 10 mg/ml, FOS 5, 10, 20 mg/ml, COS 2.5, 5, 10 mg/ml.

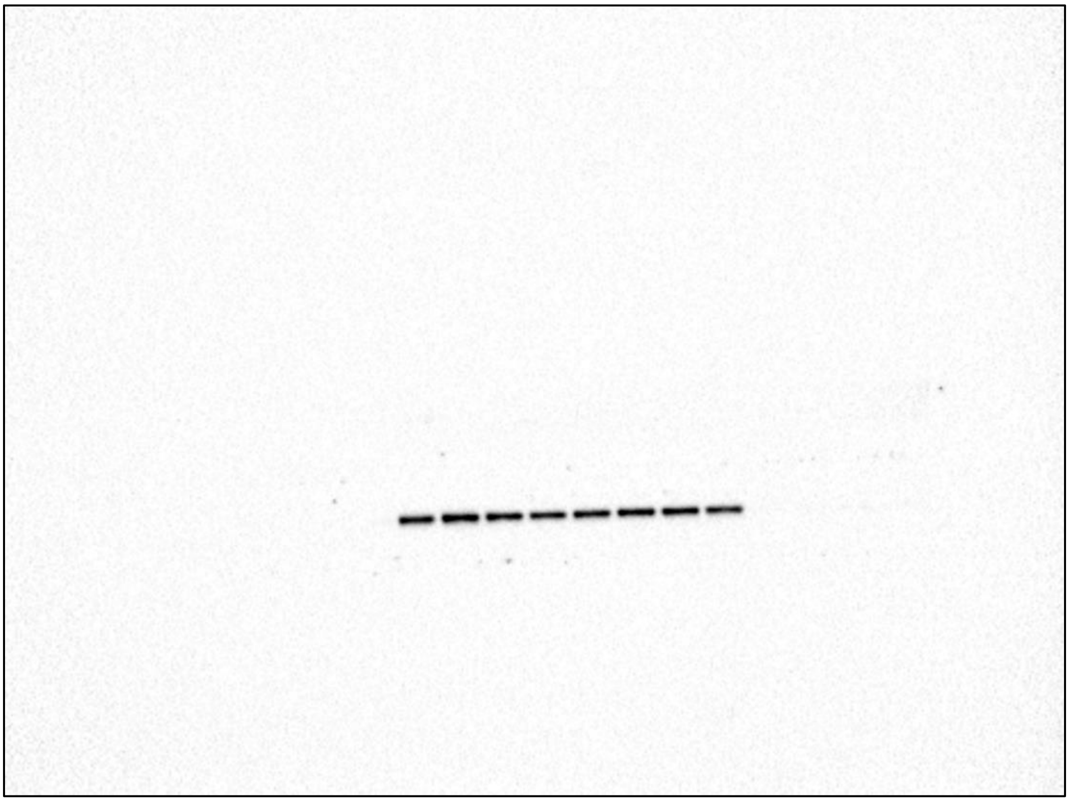

Repeat 2 of β-actin, lanes from left to right: Control, Model, RES 25, 50, 100 μM, ALA 25, 50, 100 μM.

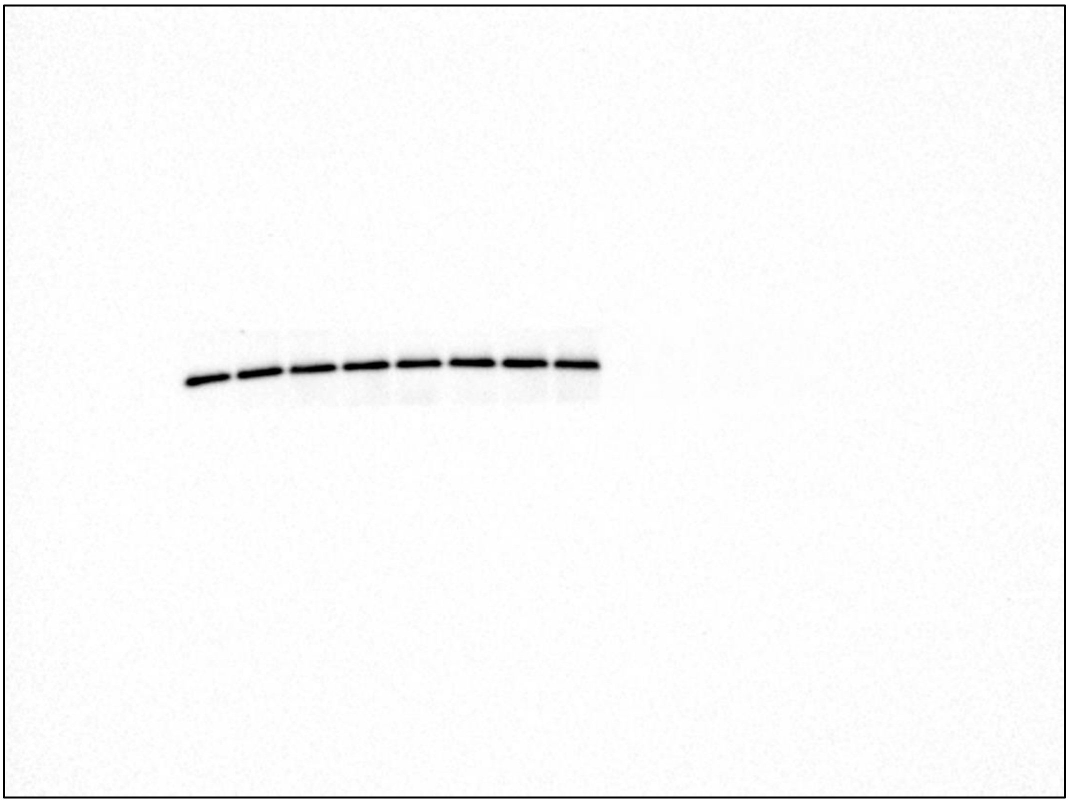

Repeat 2 of β-actin, lanes from left to right: Control, Model, Arg 0.5, 1.0, 2.0 mM, EPA 12.5, 25, 50 μM.

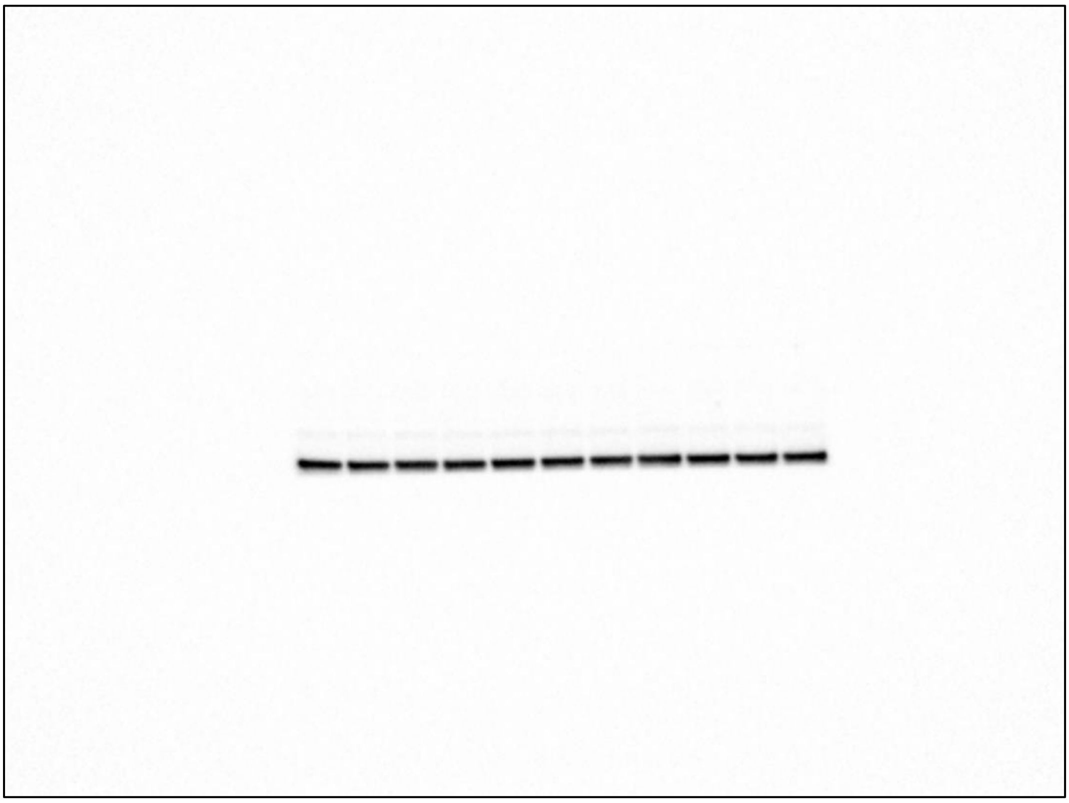

Repeat 3 of β-actin, lanes from left to right: Control, Model, GOS 2.5, 5, 10 mg/ml, FOS 5, 10, 20 mg/ml, COS 2.5, 5, 10 mg/ml.

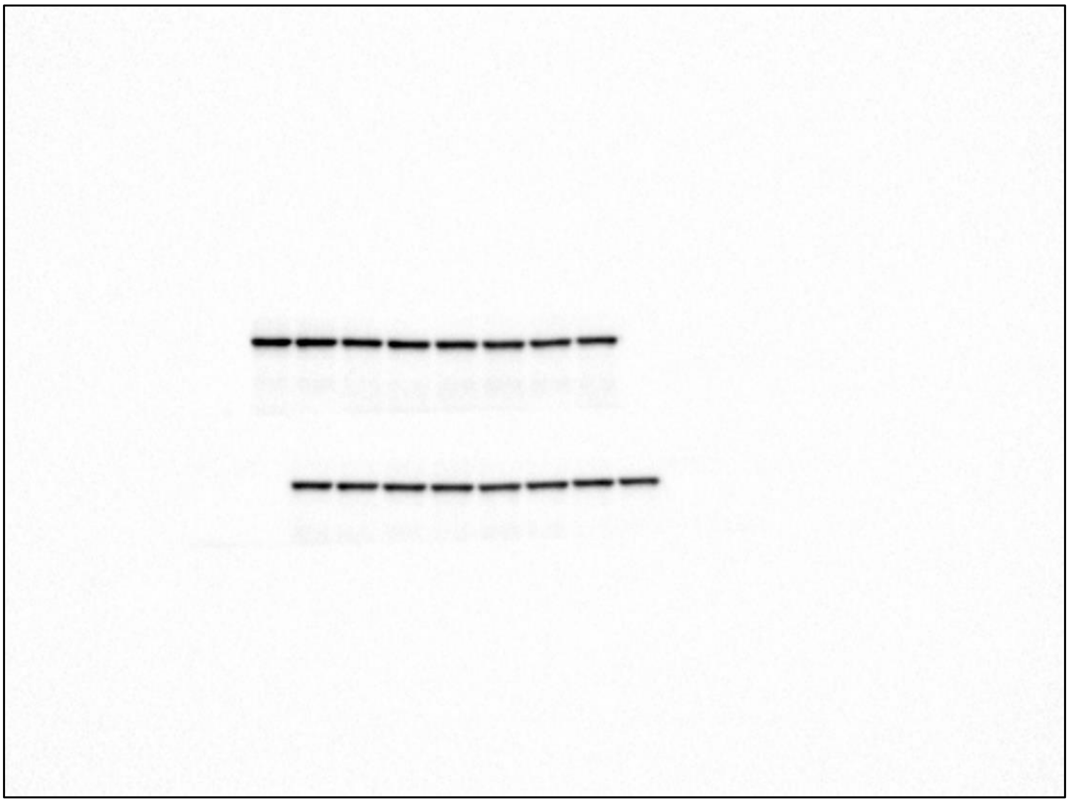

Repeat 3 of β-actin, upper lanes from left to right: Control, Model, RES 25, 50, 100 μM, ALA 25, 50, 100 μM; lower lanes from left to right: Control, Model, Arg 0.5, 1.0, 2.0 mM, EPA 12.5, 25, 50 μM.

Original blot images supporting Figure S3:

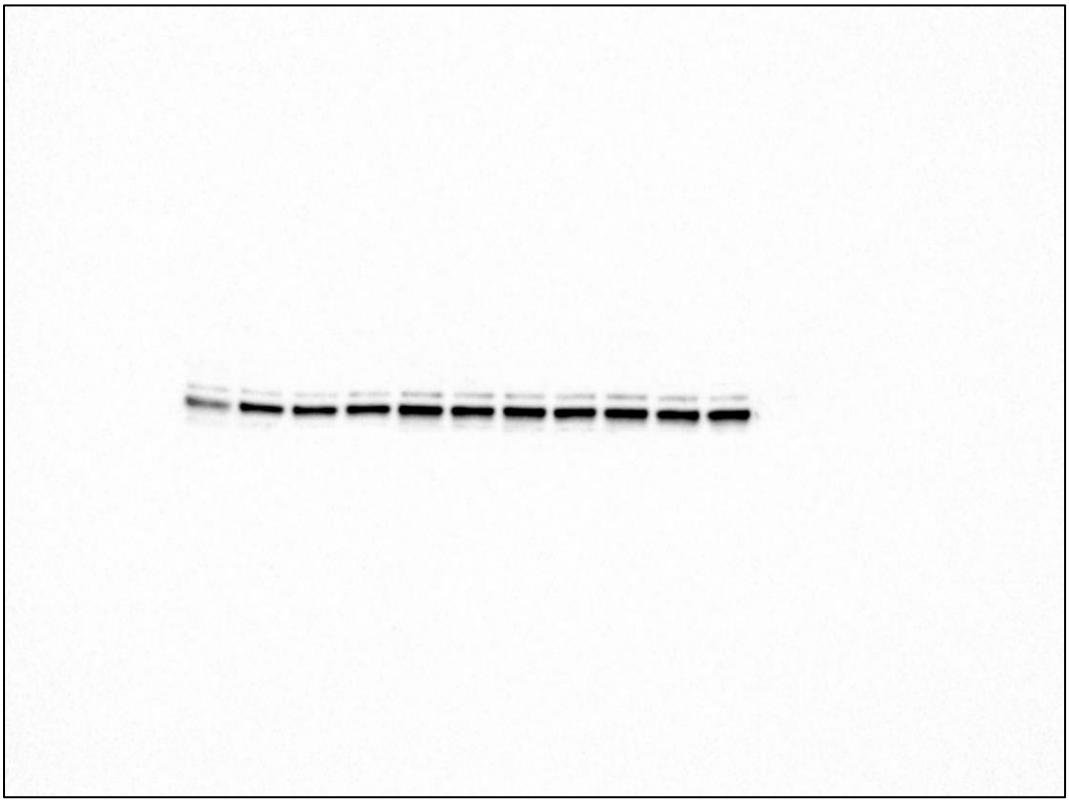

Repeat 1 of HIF-1α, lanes from left to right: Control, Model, GOS 2.5, 5, 10 mg/ml, FOS 5, 10, 20 mg/ml, COS 2.5, 5, 10 mg/ml.

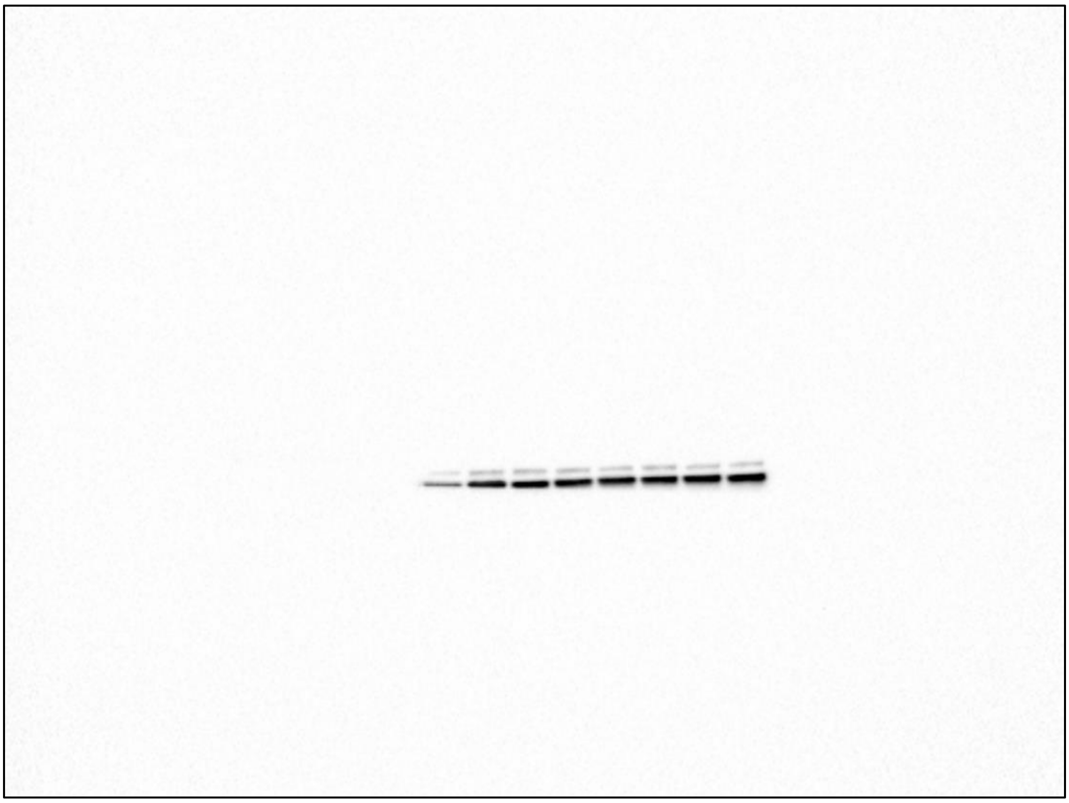

Repeat 1 of HIF-1α, lanes from left to right: Control, Model, RES 25, 50, 100 μM, ALA 25, 50, 100 μM.

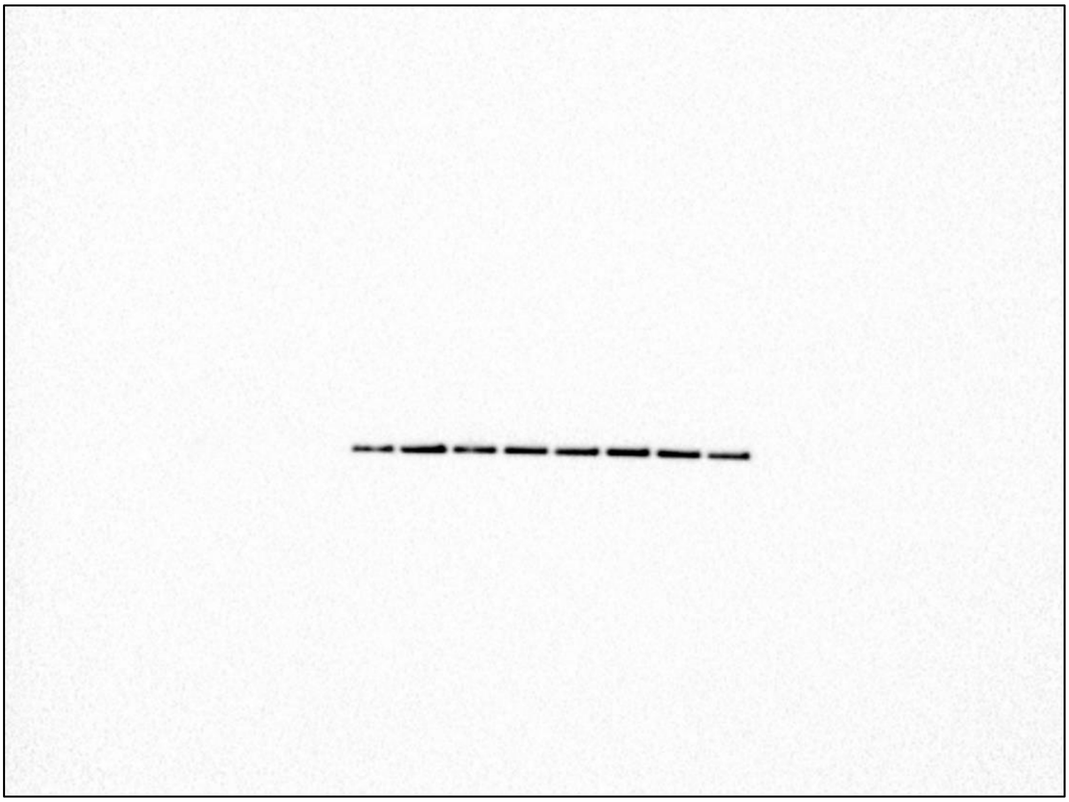

Repeat 1 of HIF-1α, lanes from left to right: Control, Model, Arg 0.5, 1.0, 2.0 mM, EPA 12.5, 25, 50 μM.

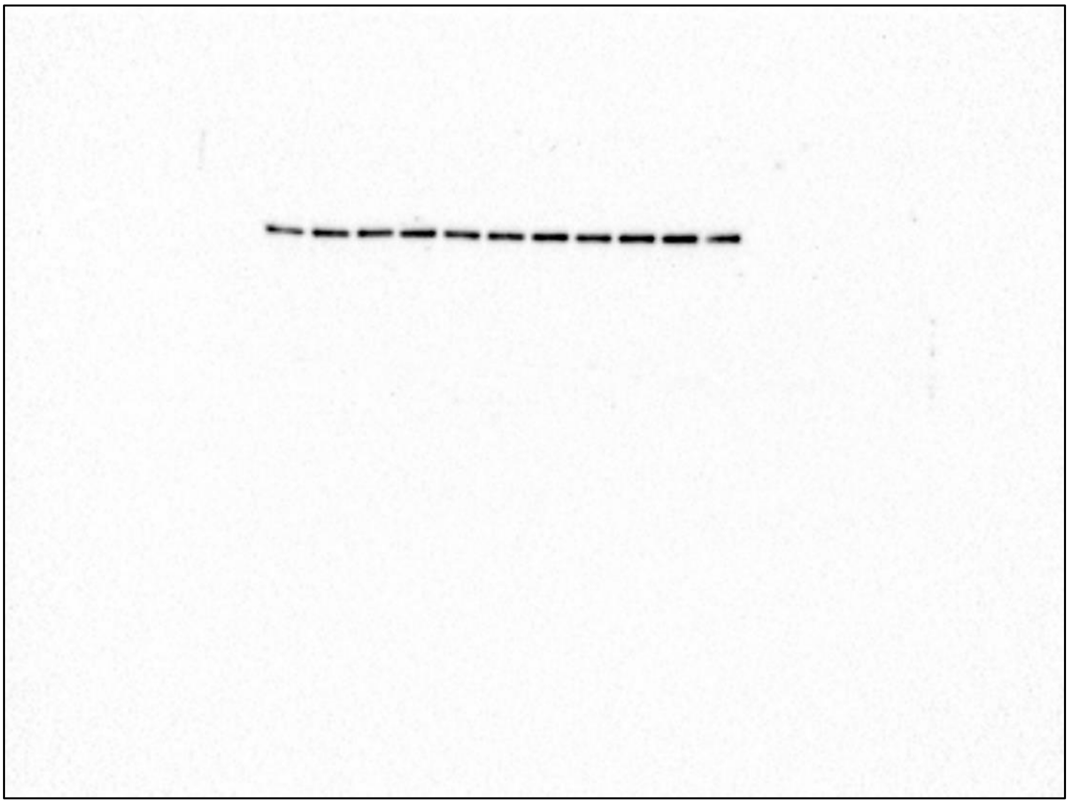

Repeat 2 of HIF-1α, lanes from left to right: Control, Model, GOS 2.5, 5, 10 mg/ml, FOS 5, 10, 20 mg/ml, COS 2.5, 5, 10 mg/ml.

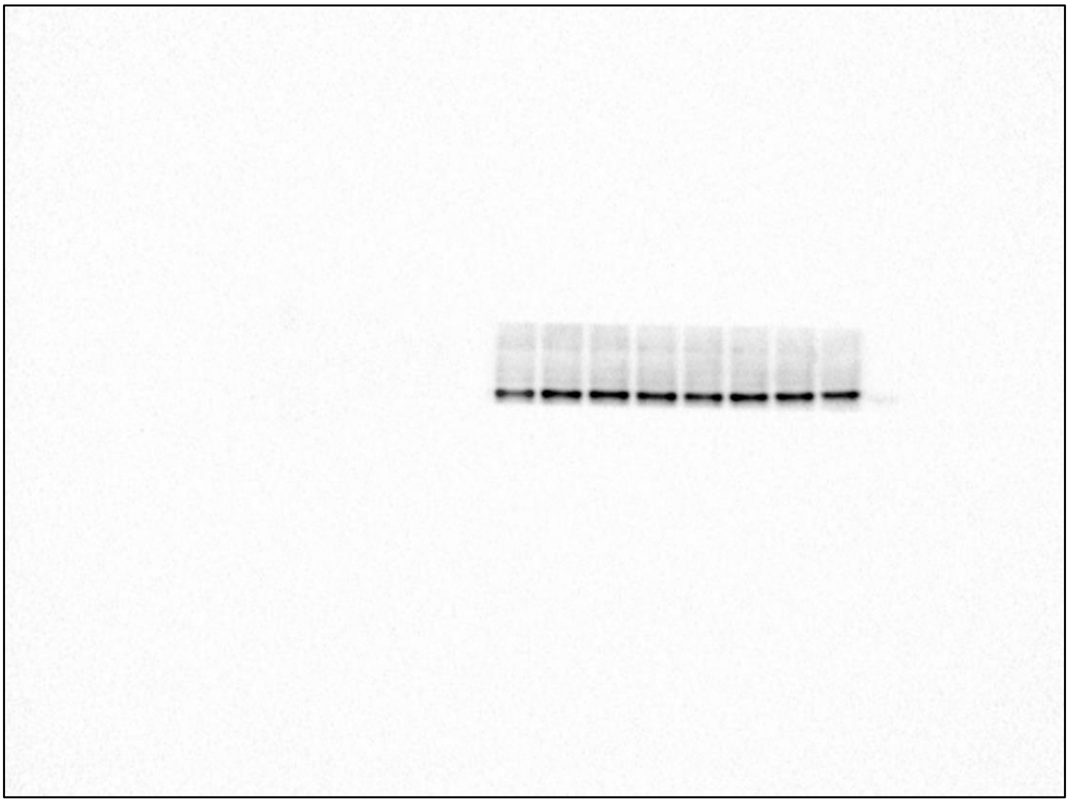

Repeat 2 of HIF-1α, lanes from left to right: Control, Model, RES 25, 50, 100 μM, ALA 25, 50, 100 μM.

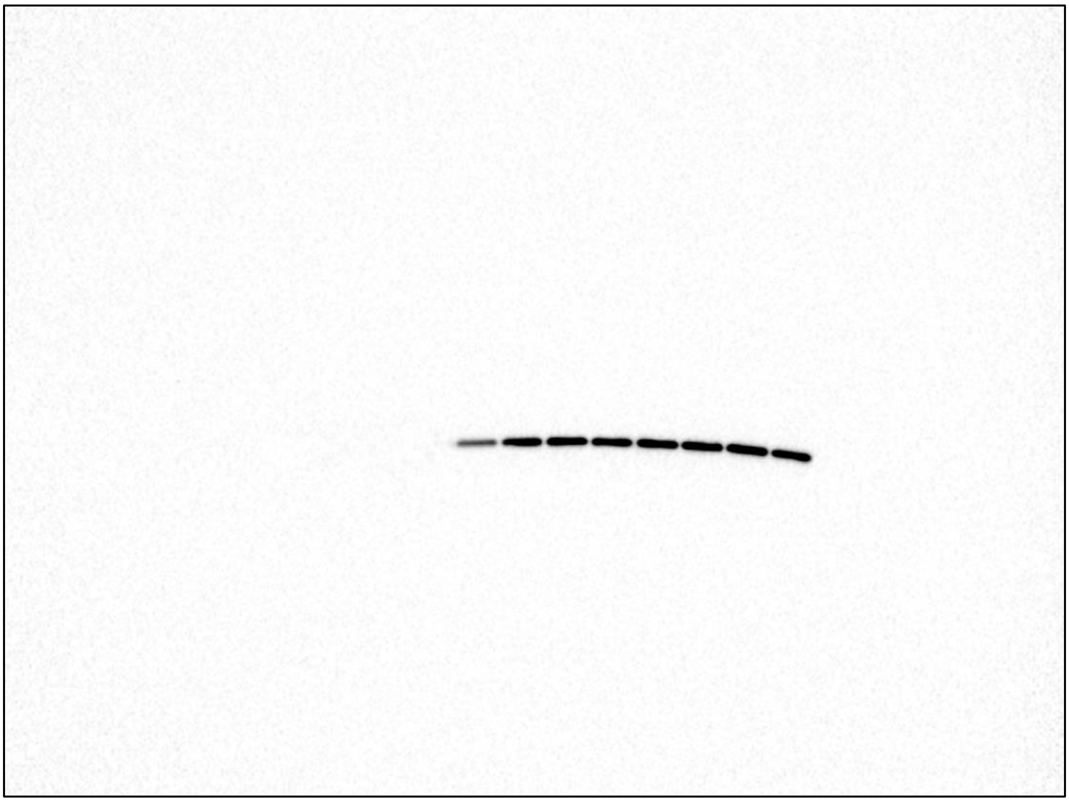

Repeat 2 of HIF-1α, lanes from left to right: Control, Model, Arg 0.5, 1.0, 2.0 mM, EPA 12.5, 25, 50 μM.

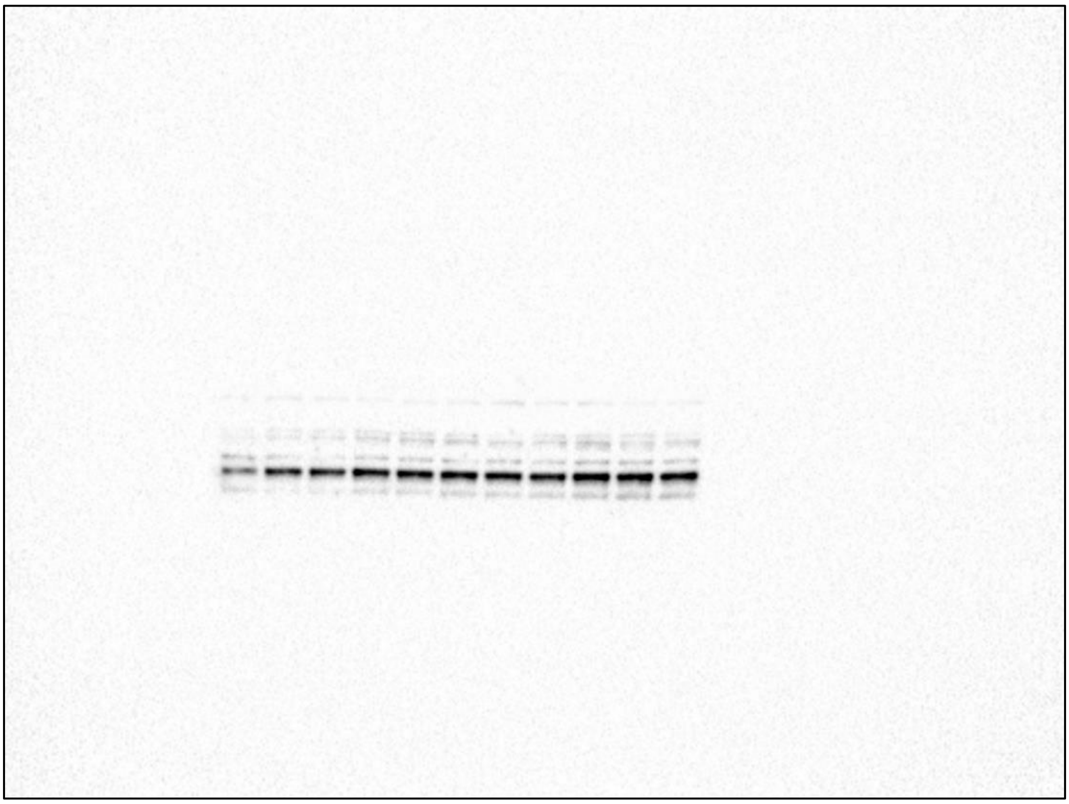

Repeat 3 of HIF-1α, lanes from left to right: Control, Model, GOS 2.5, 5, 10 mg/ml, FOS 5, 10, 20 mg/ml, COS 2.5, 5, 10 mg/ml.

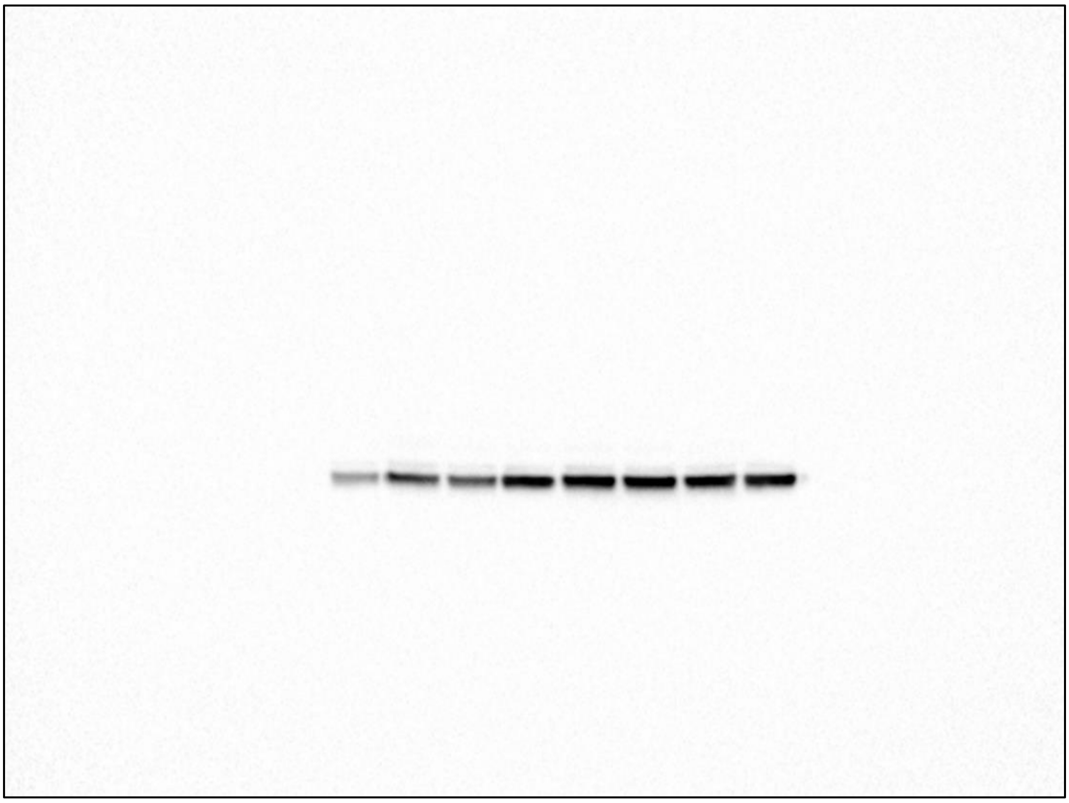

Repeat 3 of HIF-1α, lanes from left to right: Control, Model, RES 25, 50, 100 μM, ALA 25, 50, 100 μM.

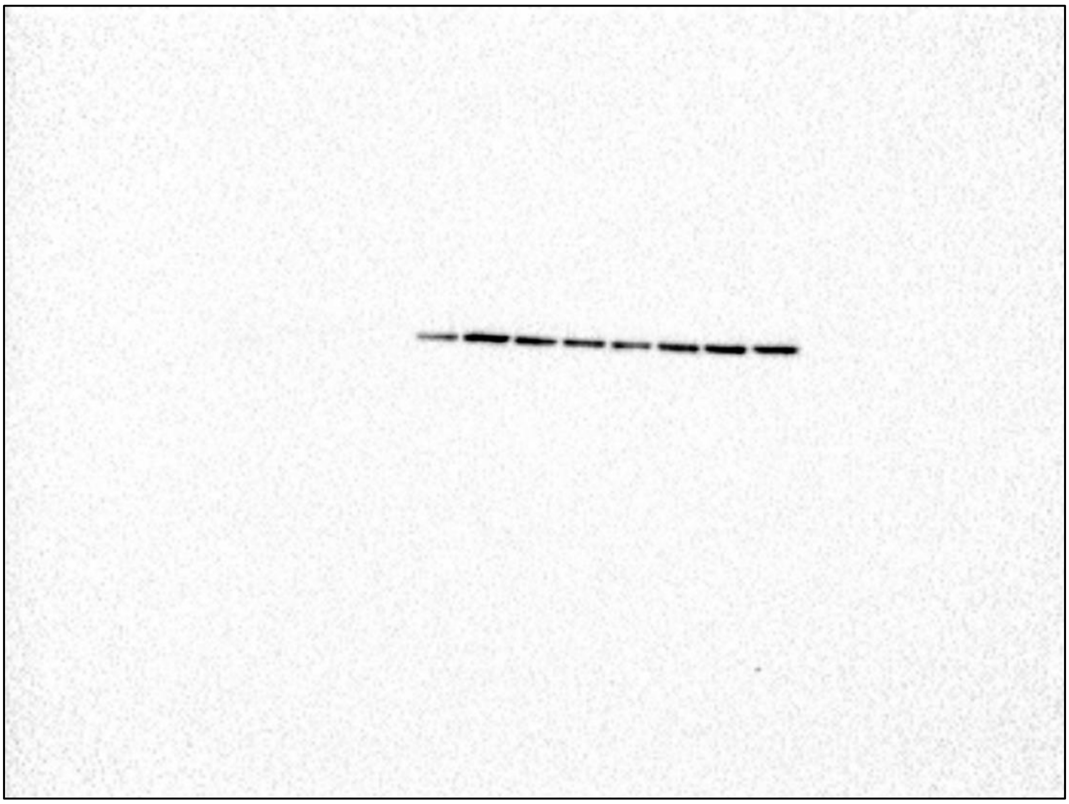

Repeat 3 of HIF-1α, lanes from left to right: Control, Model, Arg 0.5, 1.0, 2.0 mM, EPA 12.5, 25, 50 μM.

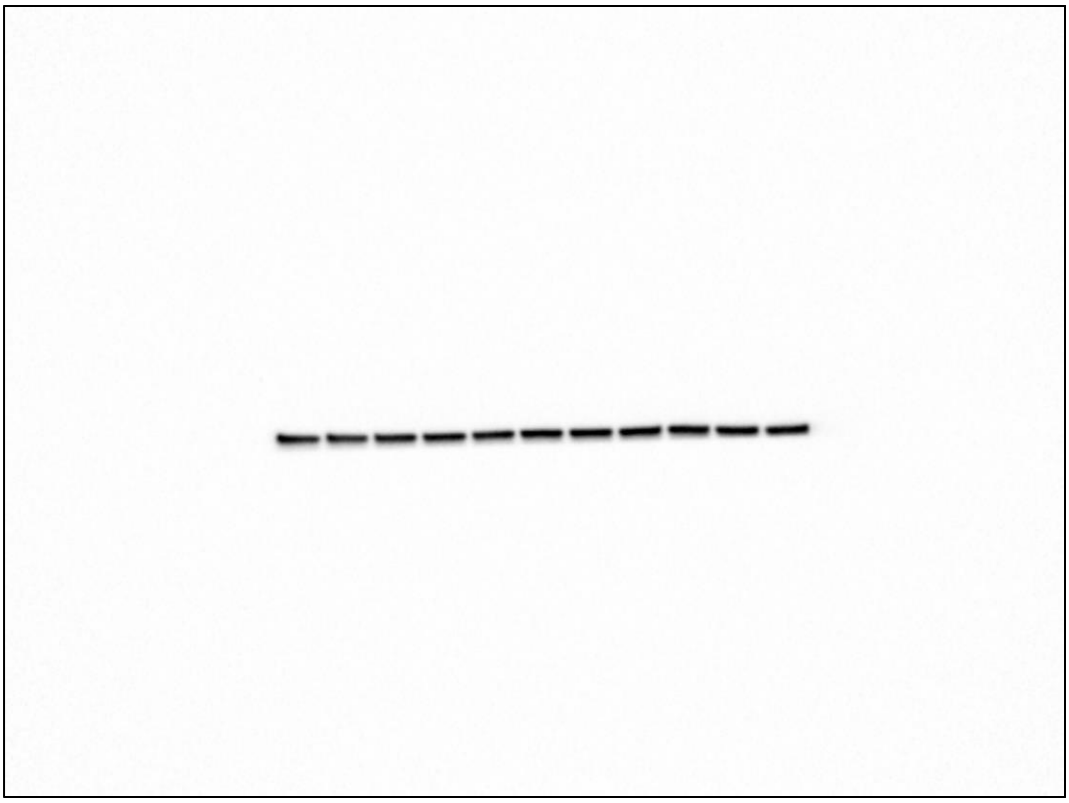

Repeat 1 of β-actin, lanes from left to right: Control, Model, GOS 2.5, 5, 10 mg/ml, FOS 5, 10, 20 mg/ml, COS 2.5, 5, 10 mg/ml.

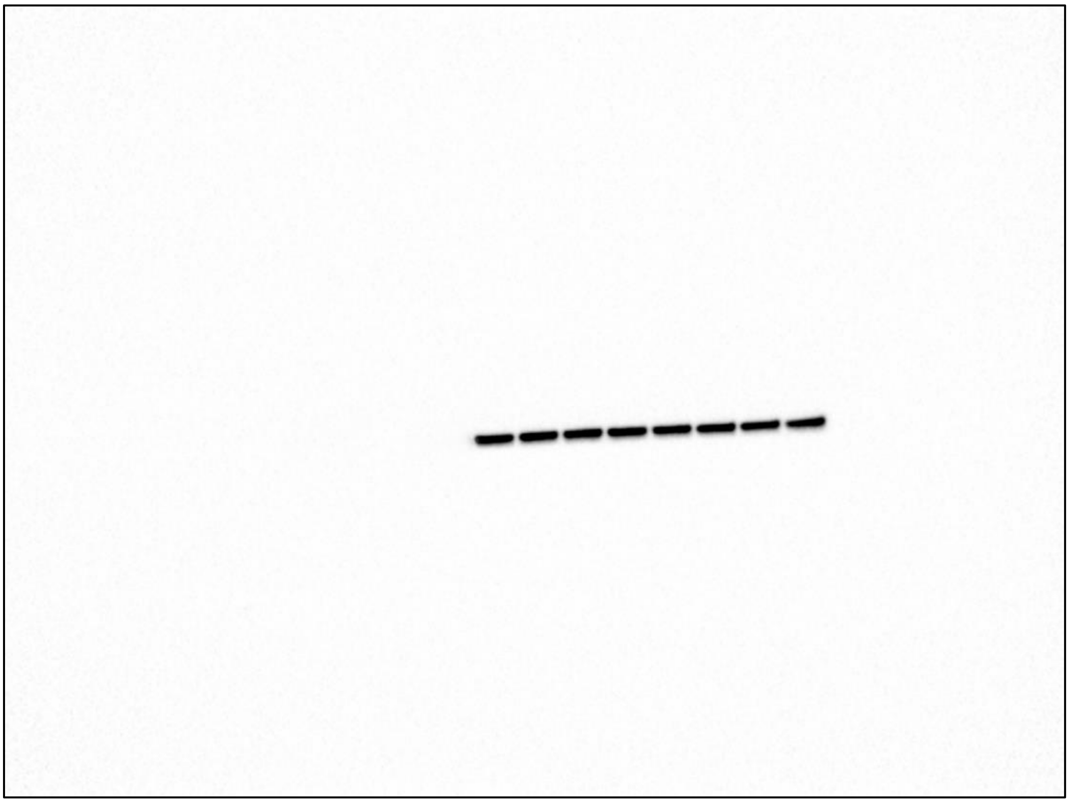

Repeat 1 of β-actin, lanes from left to right: Control, Model, RES 25, 50, 100 μM, ALA 25, 50, 100 μM.

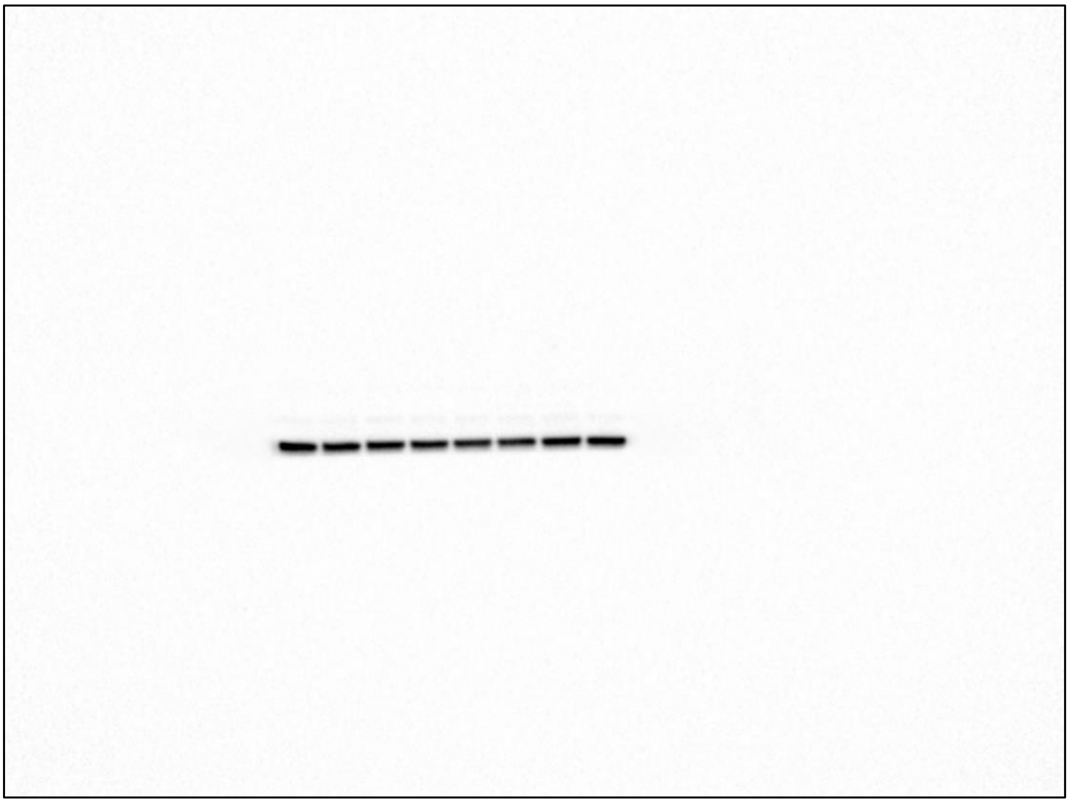

Repeat 1 of β-actin, lanes from left to right: Control, Model, Arg 0.5, 1.0, 2.0 mM, EPA 12.5, 25, 50 μM.

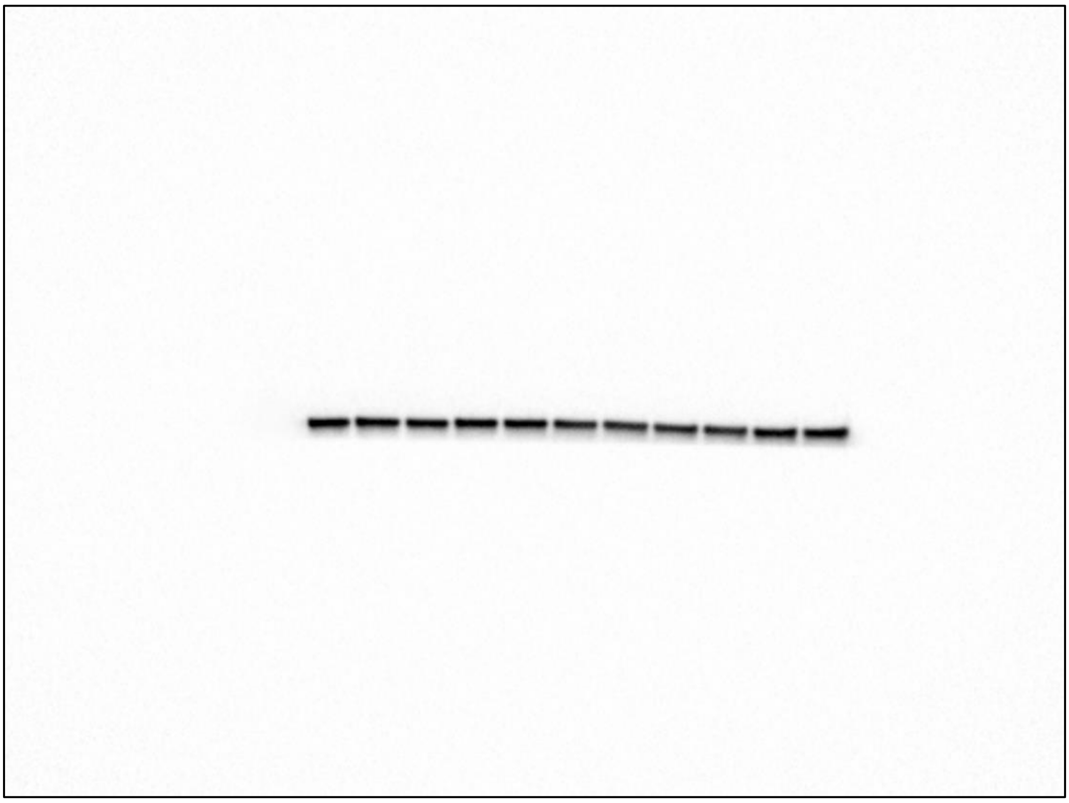

Repeat 2 of β-actin, lanes from left to right: Control, Model, GOS 2.5, 5, 10 mg/ml, FOS 5, 10, 20 mg/ml, COS 2.5, 5, 10 mg/ml.

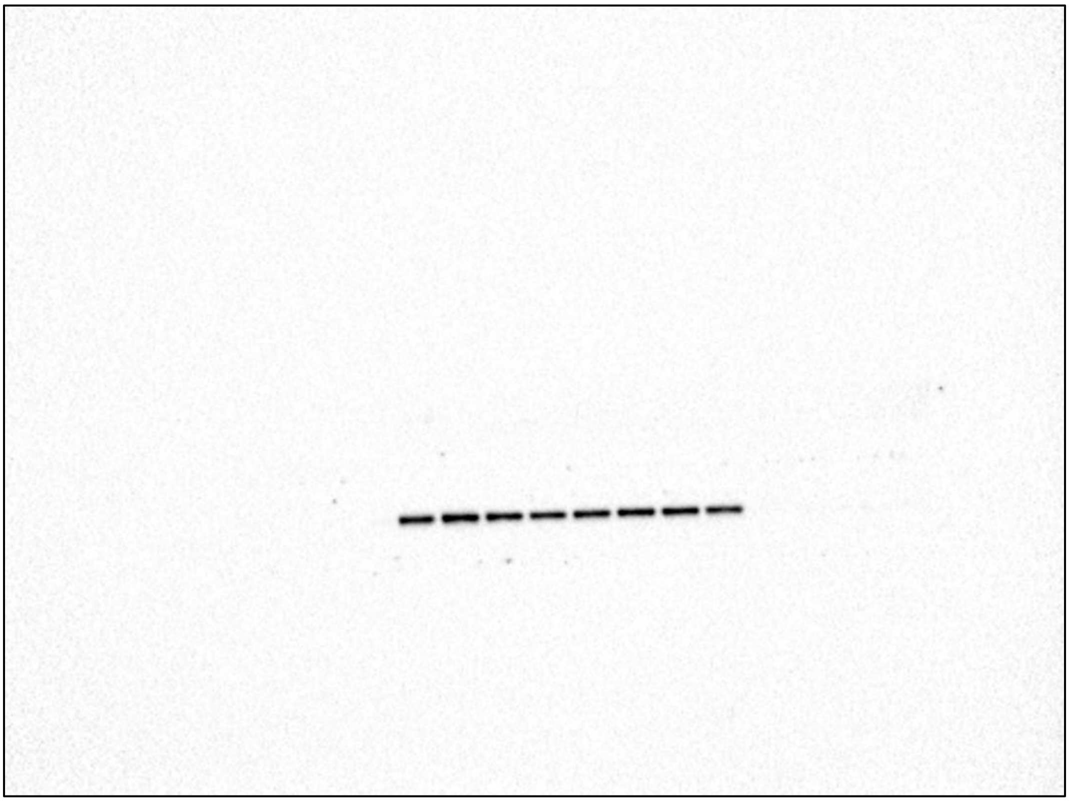

Repeat 2 of β-actin, lanes from left to right: Control, Model, RES 25, 50, 100 μM, ALA 25, 50, 100 μM.

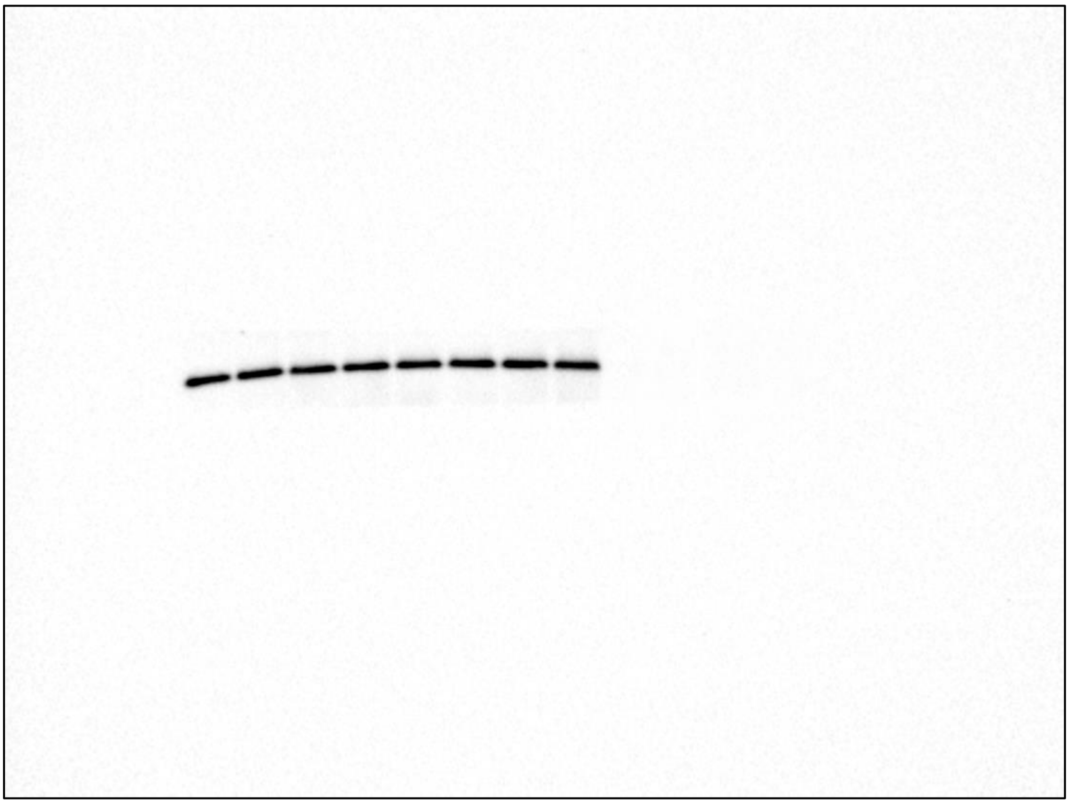

Repeat 2 of β-actin, lanes from left to right: Control, Model, Arg 0.5, 1.0, 2.0 mM, EPA 12.5, 25, 50 μM.

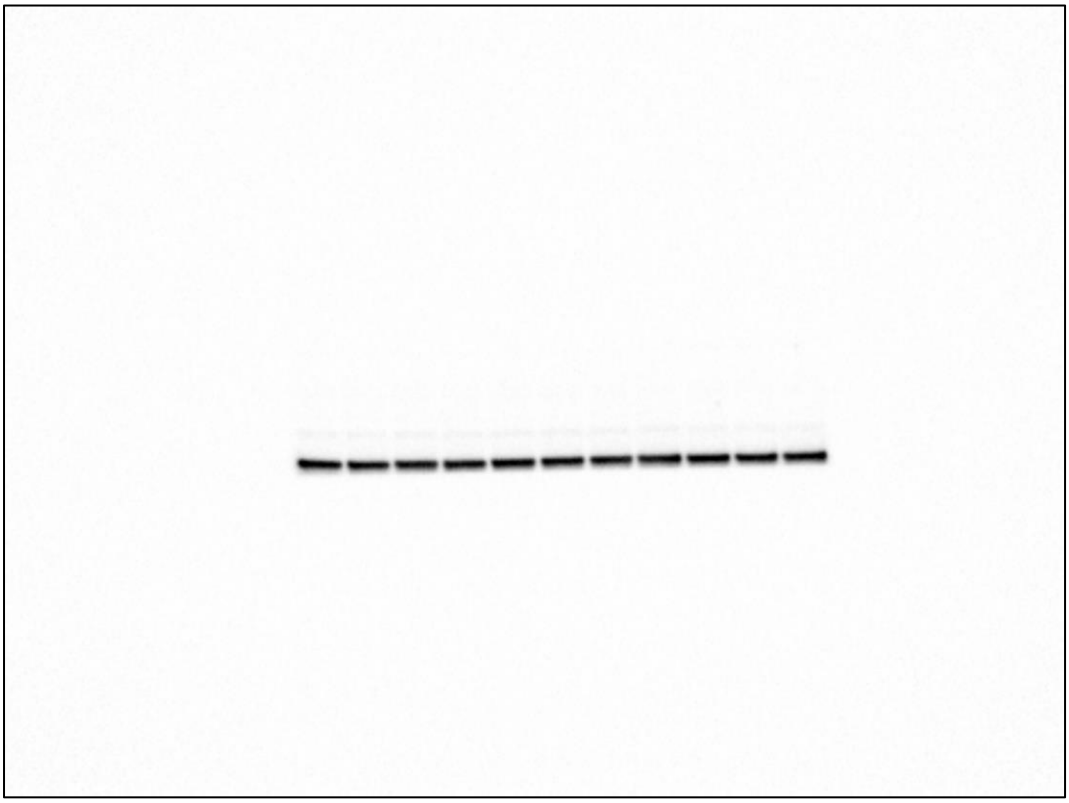

Repeat 3 of β-actin, lanes from left to right: Control, Model, GOS 2.5, 5, 10 mg/ml, FOS 5, 10, 20 mg/ml, COS 2.5, 5, 10 mg/ml.

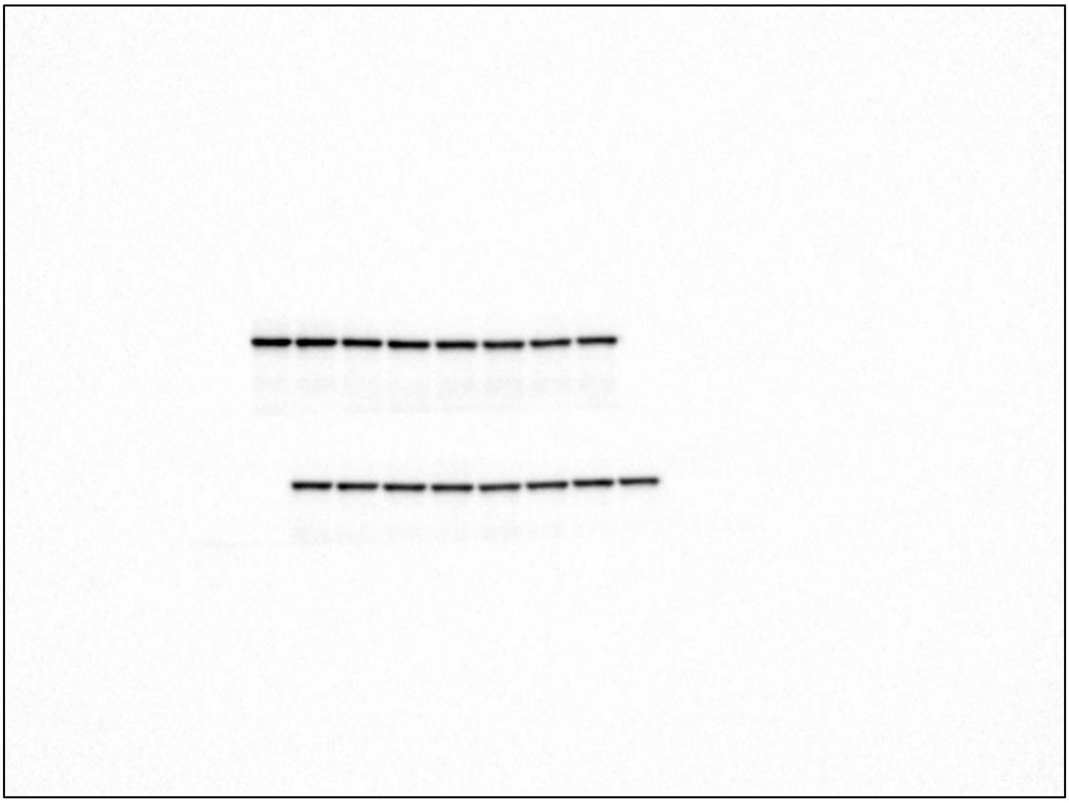

Repeat 3 of β-actin, upper lanes from left to right: Control, Model, RES 25, 50, 100 µM, ALA 25, 50, 100 µM; lower lanes from left to right: Control, Model, Arg 0.5, 1.0, 2.0 mM, EPA 12.5, 25, 50 µM.
