# Supplementary material for: Differential Effects of Oligosaccharides, Antioxidants, Amino Acids and PUFAs on Heat/Hypoxia-Induced Epithelial Injury in a Caco-2/HT-29 Co-Culture Model
Source: Int J Mol Sci. 2023 Jan 6;24(2):1111. doi: 10.3390/ijms24021111 (PMC9861987; doi:10.3390/ijms24021111)
Supplement: Supplementary file 1 [file ijms-24-01111-s001.zip › ijms-2089841-supplementary.pdf]

Supplementary materials to

"Differential effects of oligosaccharides, antioxidants, amino acids and PUFAs on heat/hypoxia-induced epithelial injury in a Caco-2/HT-29 co-culture model"

Puqiao Lian[1], Paul A. J. Henricks[1], Harry J. Wichers[2], Gert Folkerts[1], Saskia Braber[1]

[1] Division of Pharmacology, Utrecht Institute for Pharmaceutical Sciences, Faculty of Science, Utrecht University, Utrecht, The Netherlands

[2] Food & Biobased Research, Wageningen University, Wageningen, the Netherlands

Corresponding author:

S. Braber

Utrecht University, Department of Pharmaceutical Sciences, Division of Pharmacology

Universiteitsweg 99, 3584 CG, Utrecht, The Netherlands

Telephone: +31 302531599

Email: s.braber@uu.nl

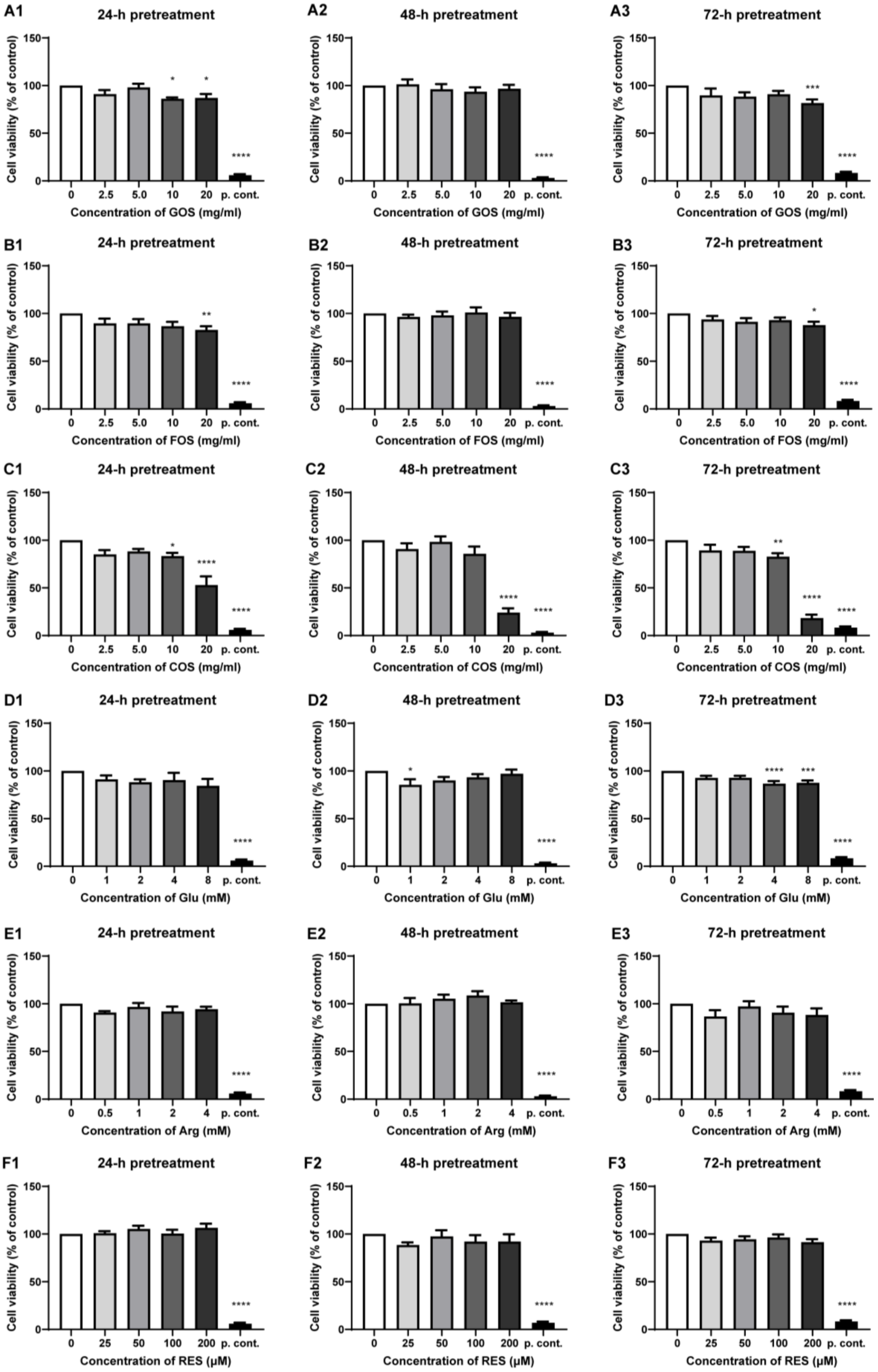
A1
24-h pretreatment
Cell viability (% of control)
150
100
50
0
*
*
****
0
2.5
5.0
10
20
p. cont.
Concentration of GOS (mg/ml)
A2
48-h pretreatment
Cell viability (% of control)
150
100
50
0
****
0
2.5
5.0
10
20
p. cont.
Concentration of GOS (mg/ml)
A3
72-h pretreatment
Cell viability (% of control)
150
100
50
0
***
****
0
2.5
5.0
10
20
p. cont.
Concentration of GOS (mg/ml)
B1
24-h pretreatment
Cell viability (% of control)
150
100
50
0
**
****
0
2.5
5.0
10
20
p. cont.
Concentration of FOS (mg/ml)
B2
48-h pretreatment
Cell viability (% of control)
150
100
50
0
****
0
2.5
5.0
10
20
p. cont.
Concentration of FOS (mg/ml)
B3
72-h pretreatment
Cell viability (% of control)
150
100
50
0
*
****
0
2.5
5.0
10
20
p. cont.
Concentration of FOS (mg/ml)
C1
24-h pretreatment
Cell viability (% of control)
150
100
50
0
*
****
****
0
2.5
5.0
10
20
p. cont.
Concentration of COS (mg/ml)
C2
48-h pretreatment
Cell viability (% of control)
150
100
50
0
****
****
0
2.5
5.0
10
20
p. cont.
Concentration of COS (mg/ml)
C3
72-h pretreatment
Cell viability (% of control)
150
100
50
0
**
****
****
0
2.5
5.0
10
20
p. cont.
Concentration of COS (mg/ml)
D1
24-h pretreatment
Cell viability (% of control)
150
100
50
0
****
0
1
2
4
8
p. cont.
Concentration of Glu (mM)
D2
48-h pretreatment
Cell viability (% of control)
150
100
50
0
*
****
0
1
2
4
8
p. cont.
Concentration of Glu (mM)
D3
72-h pretreatment
Cell viability (% of control)
150
100
50
0
****
***
****
0
1
2
4
8
p. cont.
Concentration of Glu (mM)
E1
24-h pretreatment
Cell viability (% of control)
150
100
50
0
****
0
0.5
1
2
4
p. cont.
Concentration of Arg (mM)
E2
48-h pretreatment
Cell viability (% of control)
150
100
50
0
****
0
0.5
1
2
4
p. cont.
Concentration of Arg (mM)
E3
72-h pretreatment
Cell viability (% of control)
150
100
50
0
****
0
0.5
1
2
4
p. cont.
Concentration of Arg (mM)
F1
24-h pretreatment
Cell viability (% of control)
150
100
50
0
****
0
25
50
100
200
p. cont.
Concentration of RES (μM)
F2
48-h pretreatment
Cell viability (% of control)
150
100
50
0
****
0
25
50
100
200
p. cont.
Concentration of RES (μM)
F3
72-h pretreatment
Cell viability (% of control)
150
100
50
0
****
0
25
50
100
200
p. cont.
Concentration of RES (μM)

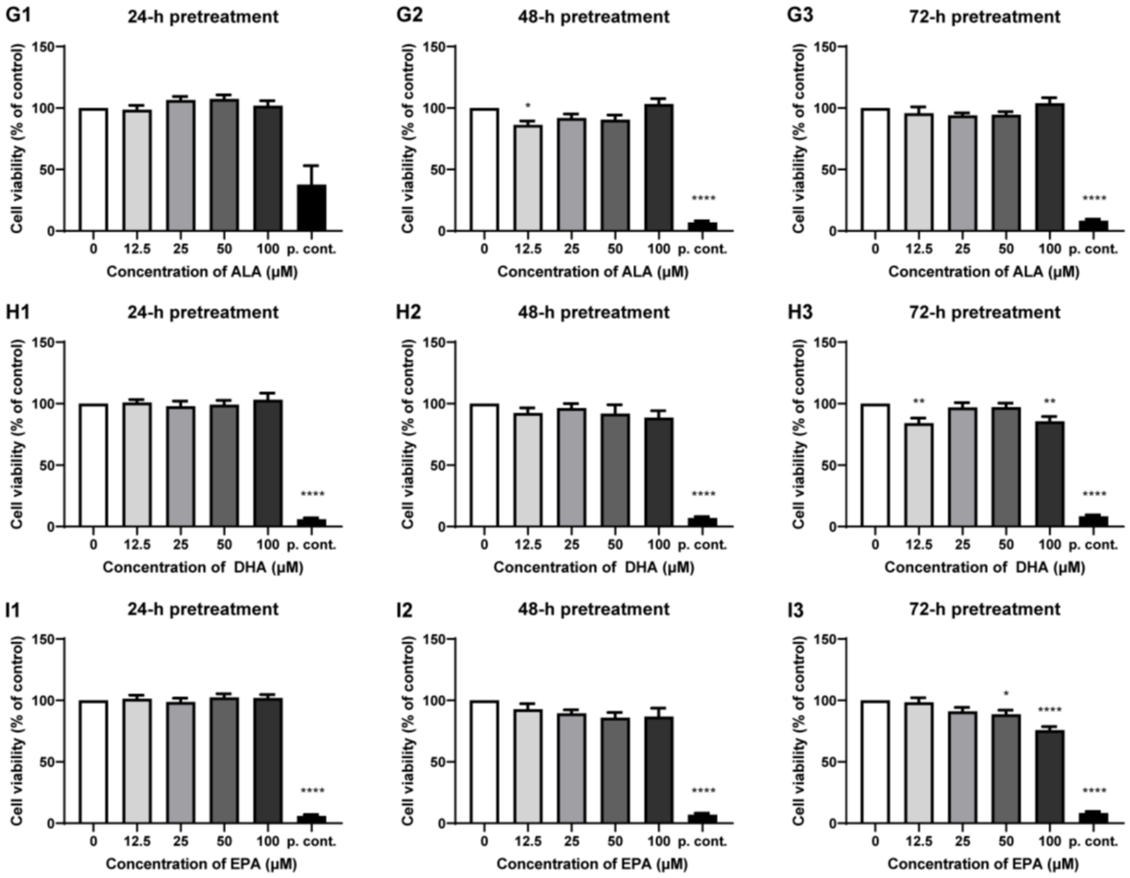

Figure S1. Cytotoxicity of the 9 nutritional components, GOS (**A1**-**A3**), FOS (**B1**-**B3**), COS (**C1**-**C3**), Glu (**D1**-**D3**), Arg (**E1**-**E3**), RES (**F1**-**F3**), ALA (**G1**-**G3**), DHA (**H1**-**H3**) and EPA (**I1**-**I3**), was evaluated by using a MTT assay without hypoxia and heat treatment. Four concentrations of the nutritional components were co-incubated with Caco-2/HT29 cells for 24, 48 and 72 h. All values were presented as means ± SEM (N=3, n=6 (negative control, positive control, the lowest and the highest concentration groups in each figure) and n=3 (all other groups)). Statistical differences were analyzed by two-way ANOVA followed by the Bonferroni's multiple comparison test. *p<0.05, **p<0.01, ***p<0.001 and ****p<0.0001 versus control. GOS, galacto-oligosaccharides; FOS, fructo-oligosaccharides, COS, chitosan oligosaccharides; Glu, l-glutamine; Arg, l-arginine; RES, resveratrol; ALA, α-lipoic acid; DHA, docosahexaenoic acid; EPA, eicosapentaenoic acid. p. cont, positive control.

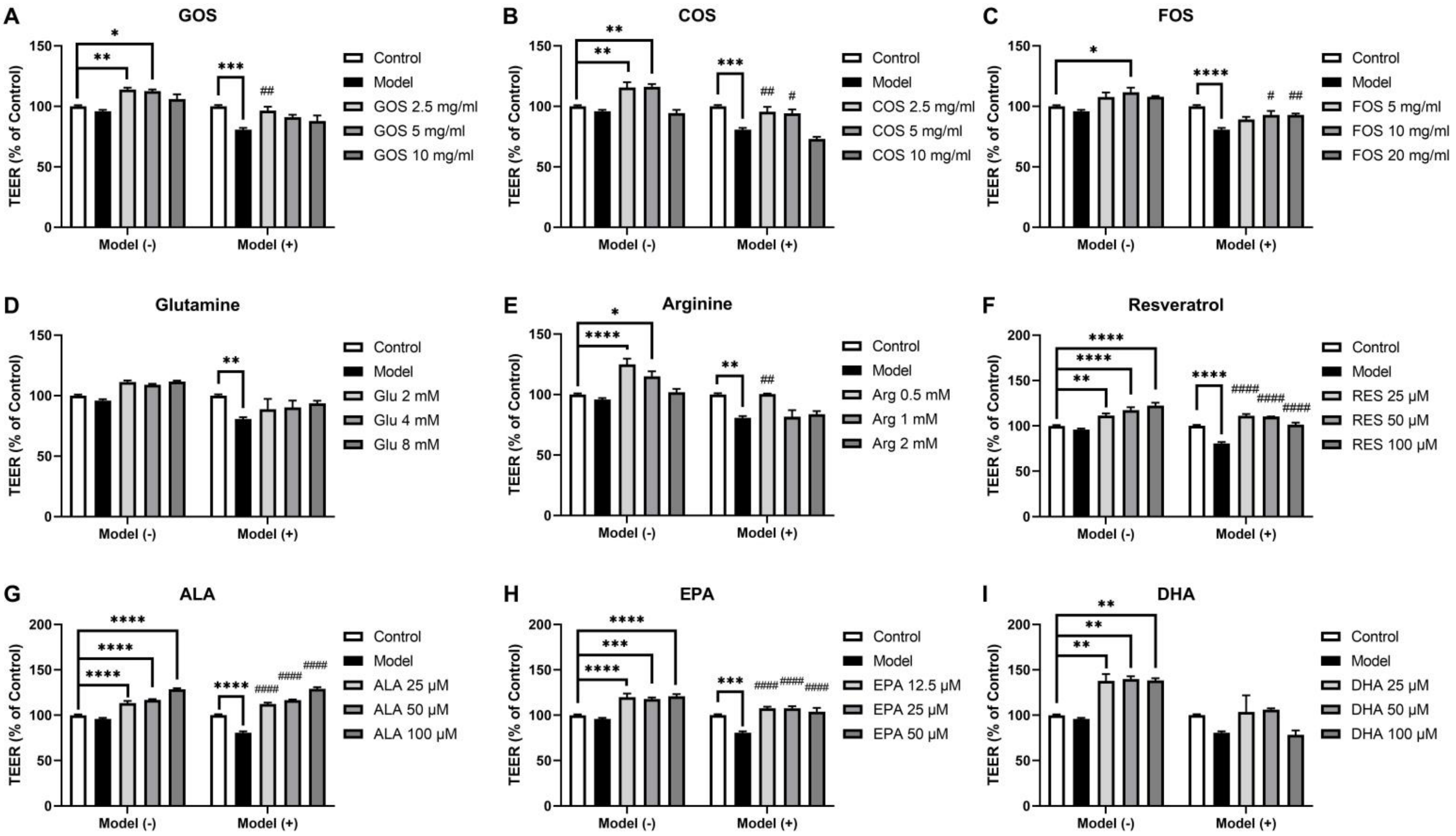

A
GOS
B
COS
C
FOS
D
Glutamine
E
Arginine
F
Resveratrol
G
ALA
H
EPA
I
DHA
TEER (% of Control)
Model (-)
Model (+)
Control
Model
GOS 2.5 mg/ml
GOS 5 mg/ml
GOS 10 mg/ml
COS 2.5 mg/ml
COS 5 mg/ml
COS 10 mg/ml
FOS 5 mg/ml
FOS 10 mg/ml
FOS 20 mg/ml
Glu 2 mM
Glu 4 mM
Glu 8 mM
Arg 0.5 mM
Arg 1 mM
Arg 2 mM
RES 25 μM
RES 50 μM
RES 100 μM
ALA 25 μM
ALA 50 μM
ALA 100 μM
EPA 12.5 μM
EPA 25 μM
EPA 50 μM
DHA 25 μM
DHA 50 μM
DHA 100 μM

Figure S2. TEER values of Caco-2/HT-29 cell monolayer pre-treated with GOS (**A**), FOS (**B**), COS (**C**), Glu (**D**), Arg (**E**), RES (**F**), ALA (**G**), DHA (**H**) and EPA (**I**) for 48 h prior to hypoxia and heat exposure (2 h). Before and after hypoxia and heat exposure, TEER values were determined by using an epithelial volt-ohm meter. All values were presented as means ± SEM (N=3, n=3). Statistical differences were analyzed by two-way ANOVA followed by the Bonferroni's multiple comparison test. *p<0.05, **p<0.01, ***p<0.001 and ****p<0.0001 versus control; #p<0.05, ##p<0.01 and ####p<0.0001 versus model. GOS, galacto-oligosaccharides; FOS, fructo-oligosaccharides, COS, chitosan oligosaccharides; Glu, l-glutamine; Arg, l-arginine; RES, resveratrol; ALA, α-lipoic acid; DHA, docosahexaenoic acid; EPA, eicosapentaenoic acid.

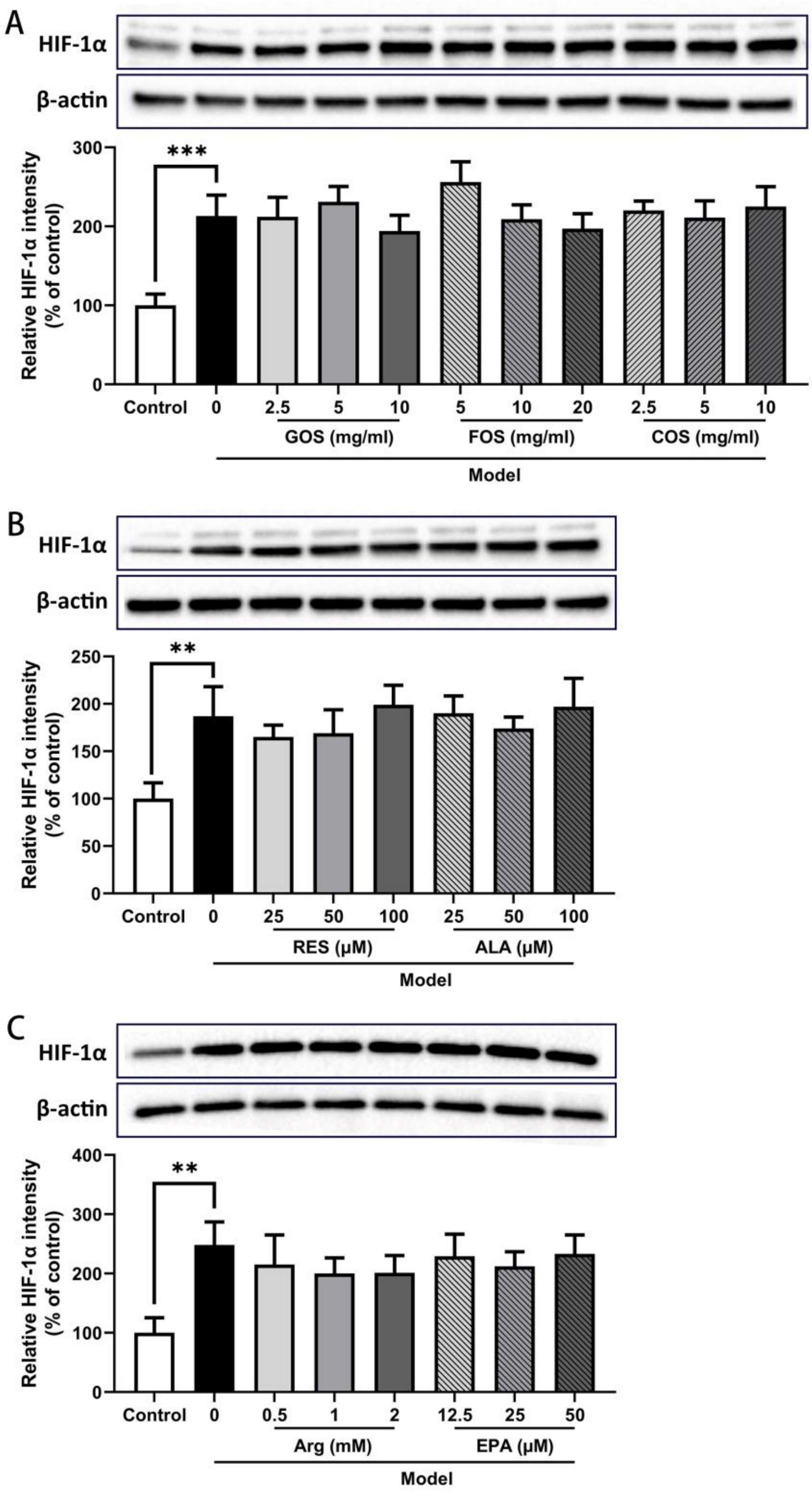

Figure S3. Relative HIF-1α protein expression level. Caco-2/HT-29 cell monolayer was pre-incubated with GOS, FOS, COS (**A**), RES, ALA (**B**), Arg and EPA (**C**) for 48 h then exposed to hypoxia and heat treatment (2 h). HIF-1α preotein expression was determined by WB and nomalized to β-actin. All values were presented as means ± SEM (N=3, n=2 (GOS, FOS, COS group) and n=3 (all other groups)). Statistical differences were analyzed by two-way ANOVA followed by the Bonferroni's multiple comparison test. **p<0.01 and ***p<0.001 versus control. GOS, galacto-oligosaccharides; FOS, fructo-oligosaccharides, COS, chitosan oligosaccharides; ALA, α-lipoic acid; RES, resveratrol; Arg, l-arginine; EPA, eicosapentaenoic acid.

	Control			Model			GOS 2.5 mg/ml			GOS 5 mg/ml			GOS 10 mg/ml					
	**Repeat 1**	**Repeat 2**	**Repeat 3**	**Repeat 1**	**Repeat 2**	**Repeat 3**	**Repeat 1**	**Repeat 2**	**Repeat 3**	**Repeat 1**	**Repeat 2**	**Repeat 3**	**Repeat 1**	**Repeat 2**	**Repeat 3**			
**Triplicate 1**	0.65215	0.590798	0.385412	0.857454	0.37345	0.681506	0.639685	0.592056	0.485574	0.855532	0.540792	0.306502	0.829279	0.417007	0.434575			
**Triplicate 2**	0.746664	0.412425	0.606056	0.886951	0.467435	0.334645	0.741071	0.454847	0.783766	0.952022	0.656096	0.811684	0.915328	0.496069	0.423537			
**Triplicate 3**	0.716546	0.525277	0.915536	0.501995	0.594627	0.667246												
	**FOS 5 mg/ml**			**FOS 10 mg/ml**			**FOS 20 mg/ml**			**COS 2.5 mg/ml**			**COS 5 mg/ml**			**COS 10 mg/ml**		
	**Repeat 1**	**Repeat 2**	**Repeat 3**	**Repeat 1**	**Repeat 2**	**Repeat 3**	**Repeat 1**	**Repeat 2**	**Repeat 3**	**Repeat 1**	**Repeat 2**	**Repeat 3**	**Repeat 1**	**Repeat 2**	**Repeat 3**	**Repeat 1**	**Repeat 2**	**Repeat 3**
**Triplicate 1**	0.806847	0.437334	1.01971	0.640745	0.431409	0.107668	0.633731	0.538809	0.849447	0.762655	0.444423	0.234186	0.775662	0.526018	0.848737	0.65248	0.453781	0.241862
**Triplicate 2**	0.692147	0.596983	0.613176	0.853525	0.503728	0.179078	0.721767	0.371478	0.647205	0.715558	0.389561	0.822279	0.50905	0.497272	0.69312	0.618186	0.568789	0.804996
**Triplicate 3**																		
	**Control**			**Model**			**Arg 1 mM**			**Arg 2 mM**			**Arg 4 mM**					
	**Repeat 1**	**Repeat 2**	**Repeat 3**	**Repeat 1**	**Repeat 2**	**Repeat 3**	**Repeat 1**	**Repeat 2**	**Repeat 3**	**Repeat 1**	**Repeat 2**	**Repeat 3**	**Repeat 1**	**Repeat 2**	**Repeat 3**			
**Triplicate 1**	0.703108	0.571894	0.752331	0.844359	0.501425	0.75124	0.591313	0.484435	1.040087	0.741574	0.656786	0.334978	0.826482	0.41451	0.715579			
**Triplicate 2**	0.592899	0.621237	0.810731	0.679031	0.4153	0.657683	0.635697	0.50129	0.480523	0.62127	0.451432	0.397084	0.709482	0.419014	0.733387			
**Triplicate 3**	0.753833	0.381863	0.064433	0.556377	0.64277	0.72169	0.980823	0.542776	0.498197	0.665155	0.559764	0.56002	0.601236	0.694976	0.51394			
	**EPA 12.5 μM**			**EPA 25 μM**			**EPA 50 μM**											
	**Repeat 1**	**Repeat 2**	**Repeat 3**	**Repeat 1**	**Repeat 2**	**Repeat 3**	**Repeat 1**	**Repeat 2**	**Repeat 3**									
**Triplicate 1**	0.786702	0.526818	0.620663	0.776487	0.622313	0.860252	0.6508	0.610861	0.641418									
**Triplicate 2**	0.832175	0.523734	0.577311	0.630657	0.433649	0.24335	0.673795	0.578835	0.577947									
**Triplicate 3**	0.605684	0.524441	0.373618	0.708215	0.503534	0.803402	0.834444	0.52478	0.743541									
	**Control**			**Model**			**RES 25 μM**			**RES 50 μM**			**RES 100 μM**					
	**Repeat 1**	**Repeat 2**	**Repeat 3**	**Repeat 1**	**Repeat 2**	**Repeat 3**	**Repeat 1**	**Repeat 2**	**Repeat 3**	**Repeat 1**	**Repeat 2**	**Repeat 3**	**Repeat 1**	**Repeat 2**	**Repeat 3**			
**Triplicate 1**	0.624697	0.537771	0.976209	0.766089	0.538665	0.729742	0.676842	0.584785	0.374659	0.764653	0.536859	0.478188	0.757792	0.56839	0.470817			
**Triplicate 2**	0.624241	0.594578	0.406261	0.610101	0.556851	0.636793	0.711745	0.521842	0.625496	0.700092	0.657244	0.780962	0.70105	0.536125	0.46535			
**Triplicate 3**	0.822742	0.396152	0.636338	0.703576	0.61896	0.764077	0.819246	0.421873	0.795045	1.042872	0.473878	0.032931	0.678358	0.423984	0.355916			
	**ALA 25 μM**			**ALA 50 μM**			**ALA 100 μM**											
	**Repeat 1**	**Repeat 2**	**Repeat 3**	**Repeat 1**	**Repeat 2**	**Repeat 3**	**Repeat 1**	**Repeat 2**	**Repeat 3**									
**Triplicate 1**	0.641279	0.617514	0.54595	0.819939	0.501634	0.828766	0.851962	0.628547	0.343331									
**Triplicate 2**	0.746518	0.550037	0.505543	0.628341	0.587599	0.843728	0.892256	0.505138	0.976269									
**Triplicate 3**	0.836764	0.407442	1.414531	0.66708	0.439267	0.23451	0.611383	0.580791	0.643305									

The standard curves: $y=0.00052x-0.00832$, $r^2=0.921$ (repeat 1); $y=0.000369x-0.0071$, $r^2=0.9831$ (repeat 2); $y=0.001331x-1.265$, $r^2=0.899$ (repeat 3); y: LDH concentration (μg/ml), x: OD value.

Table S1. Raw colorimetric readings from three repeats of the LDH experiments using triplicates.

	Control			Model			GOS 2.5 mg/ml			GOS 5 mg/ml			GOS 10 mg/ml					
	**Repeat 1**	**Repeat 2**	**Repeat 3**	**Repeat 1**	**Repeat 2**	**Repeat 3**	**Repeat 1**	**Repeat 2**	**Repeat 3**	**Repeat 1**	**Repeat 2**	**Repeat 3**	**Repeat 1**	**Repeat 2**	**Repeat 3**			
**Triplicate 1**	1135	1169	1202	892	977	981	1139	1075	1191	1002	1066	1126	1118	1012	978			
**Triplicate 2**	1161	1184	1179	910	990	949	1119	1065	1215	992	1076	1130	1111	1031	946			
**Triplicate 3**	1162	1172	1202	906	968	962	1125	1042	1226	1026	1088	1116	1102	1019	954			

	FOS 5 mg/ml			FOS 10 mg/ml			FOS 20 mg/ml			COS 2.5 mg/ml			COS 5 mg/ml			COS 10 mg/ml		
	**Repeat 1**	**Repeat 2**	**Repeat 3**	**Repeat 1**	**Repeat 2**	**Repeat 3**	**Repeat 1**	**Repeat 2**	**Repeat 3**	**Repeat 1**	**Repeat 2**	**Repeat 3**	**Repeat 1**	**Repeat 2**	**Repeat 3**	**Repeat 1**	**Repeat 2**	**Repeat 3**
**Triplicate 1**	1030	986	1113	1056	1017	1174	1050	1080	1135	1058	1065	1252	1115	1114	1068	811	886	853
**Triplicate 2**	1018	984	1105	1080	1021	1159	1048	1105	1136	1069	1068	1218	1142	1135	1034	808	882	862
**Triplicate 3**	1022	1037	1117	1064	1030	1204	1042	1111	1115	1071	1057	1243	1144	1148	1044	807	917	896

	Arg 1 mM			Arg 2 mM			Arg 4 mM			EPA 12.5 µM			EPA 25 µM			EPA 50 µM		
	**Repeat 1**	**Repeat 2**	**Repeat 3**	**Repeat 1**	**Repeat 2**	**Repeat 3**	**Repeat 1**	**Repeat 2**	**Repeat 3**	**Repeat 1**	**Repeat 2**	**Repeat 3**	**Repeat 1**	**Repeat 2**	**Repeat 3**	**Repeat 1**	**Repeat 2**	**Repeat 3**
**Triplicate 1**	1148	1209	1201	1026	1011	837	1010	984	938	1208	1299	1305	1286	1235	1250	1121	1273	1271
**Triplicate 2**	1167	1202	1170	1008	1019	861	1026	974	944	1181	1279	1312	1277	1245	1277	1118	1293	1279
**Triplicate 3**	1135	1164	1199	1010	1006	835	995	1022	948	1205	1276	1304	1312	1247	1245	1076	1277	1287

	RES 25 µM			RES 50 µM			RES 100 µM			ALA 25 µM			ALA 50 µM			ALA 100 µM		
	**Repeat 1**	**Repeat 2**	**Repeat 3**	**Repeat 1**	**Repeat 2**	**Repeat 3**	**Repeat 1**	**Repeat 2**	**Repeat 3**	**Repeat 1**	**Repeat 2**	**Repeat 3**	**Repeat 1**	**Repeat 2**	**Repeat 3**	**Repeat 1**	**Repeat 2**	**Repeat 3**
**Triplicate 1**	1269	1301	1379	1266	1283	1310	1204	1188	1178	1247	1325	1391	1329	1373	1389	1456	1523	1570
**Triplicate 2**	1252	1289	1392	1288	1296	1321	1188	1197	1166	1277	1315	1360	1316	1373	1384	1465	1530	1574
**Triplicate 3**	1242	1264	1349	1256	1296	1330	1217	1230	1147	1263	1320	1369	1337	1408	1415	1416	1547	1562

Table S2. Raw TEER readings from three repeats of the experiments using triplicates.

	Control			Model			GOS 2.5 mg/ml			GOS 5 mg/ml			GOS 10 mg/ml					
	**Repeat 1**	**Repeat 2**	**Repeat 3**	**Repeat 1**	**Repeat 2**	**Repeat 3**	**Repeat 1**	**Repeat 2**	**Repeat 3**	**Repeat 1**	**Repeat 2**	**Repeat 3**	**Repeat 1**	**Repeat 2**	**Repeat 3**			
**Triplicate 1**	1.937746	1.410663	2.40579	2.100993	1.712118	3.0289	2.533384	1.66104	3.379464	1.979836	1.167967	2.815662	1.668027	1.17793	2.640906			
**Triplicate 2**	1.954747	1.424867	2.344435	2.142643	1.73866	3.05489	2.55359	1.689727	3.384672	1.98793	1.255182	2.697947	1.714981	1.196474	2.453487			
**Triplicate 3**	1.918914	1.365438	2.383693	2.20342	1.739544	2.786418												
	**FOS 5 mg/ml**			**FOS 10 mg/ml**			**FOS 20 mg/ml**			**COS 2.5 mg/ml**			**COS 5 mg/ml**			**COS 10 mg/ml**		
	**Repeat 1**	**Repeat 2**	**Repeat 3**	**Repeat 1**	**Repeat 2**	**Repeat 3**	**Repeat 1**	**Repeat 2**	**Repeat 3**	**Repeat 1**	**Repeat 2**	**Repeat 3**	**Repeat 1**	**Repeat 2**	**Repeat 3**	**Repeat 1**	**Repeat 2**	**Repeat 3**
**Triplicate 1**	2.002069	2.003014	2.938484	1.607397	1.316788	3.122225	1.436521	1.47586	2.221965	0.983459	1.458692	3.290038	1.38701	1.597586	2.425953	3.137661	1.506052	3.493746
**Triplicate 2**	2.08483	2.118604	2.892928	1.610529	1.318357	3.092005	1.427254	1.519794	2.118479	0.999161	1.440905	3.215464	1.408184	1.641812	2.399113	3.120383	1.526367	3.625816
**Triplicate 3**																		
	**Control**			**Model**			**Arg 1 mM**			**Arg 2 mM**			**Arg 4 mM**					
	**Repeat 1**	**Repeat 2**	**Repeat 3**	**Repeat 1**	**Repeat 2**	**Repeat 3**	**Repeat 1**	**Repeat 2**	**Repeat 3**	**Repeat 1**	**Repeat 2**	**Repeat 3**	**Repeat 1**	**Repeat 2**	**Repeat 3**			
**Triplicate 1**	1.545292	1.087839	2.125845	2.020768	1.646481	3.072199	2.186092	1.88052	3.890652	1.954258	1.08887	2.464539	1.908676	1.087171	3.575166			
**Triplicate 2**	1.535959	1.05797	2.085976	2.108253	1.621947	3.049029	2.171463	1.874215	3.931743	1.91963	1.169221	2.532516	1.972356	1.114675	3.471175			
**Triplicate 3**	1.594763	1.178899	2.286685	2.050526	1.5887	2.966064	2.22898	1.928044	4.055808	2.056803	1.091462	2.468642	1.96312	1.118426	3.482476			
	**EPA 12.5 µM**			**EPA 25 µM**			**EPA 50 µM**											
	**Repeat 1**	**Repeat 2**	**Repeat 3**	**Repeat 1**	**Repeat 2**	**Repeat 3**	**Repeat 1**	**Repeat 2**	**Repeat 3**									
**Triplicate 1**	1.814645	1.502482	2.789068	1.251522	1.539419	2.275219	1.713399	1.228477	1.960899									
**Triplicate 2**	1.769937	1.436944	2.720324	1.139568	1.598585	2.359314	1.730948	1.214716	2.022146									
**Triplicate 3**	1.680747	1.476457	2.647333	1.181867	1.547053	2.159161	1.853721	1.236572	1.929987									
	**Control**			**Model**			**RES 25 µM**			**RES 50 µM**			**RES 100 µM**					
	**Repeat 1**	**Repeat 2**	**Repeat 3**	**Repeat 1**	**Repeat 2**	**Repeat 3**	**Repeat 1**	**Repeat 2**	**Repeat 3**	**Repeat 1**	**Repeat 2**	**Repeat 3**	**Repeat 1**	**Repeat 2**	**Repeat 3**			
**Triplicate 1**	2.073549	1.755045	2.93939	3.776967	3.554346	5.730498	4.017644	2.384356	5.045137	2.427119	3.071869	2.713741	2.824694	1.991358	4.932203			
**Triplicate 2**	2.122422	1.680146	3.108845	3.803694	3.583298	5.70345	3.982898	2.418664	4.98923	2.292998	3.076078	2.818499	2.846985	1.934822	4.987529			
**Triplicate 3**	1.942467	1.803865	2.951171	3.635897	3.650364	5.746251	4.088521	2.337125	4.994588	2.396473	3.079912	2.659198	2.981481	2.07573	4.749617			
	**ALA 25 µM**			**ALA 50 µM**			**ALA 100 µM**											
	**Repeat 1**	**Repeat 2**	**Repeat 3**	**Repeat 1**	**Repeat 2**	**Repeat 3**	**Repeat 1**	**Repeat 2**	**Repeat 3**									
**Triplicate 1**	2.979108	3.706805	5.562992	1.585809	2.014222	2.059531	1.892697	1.880755	3.649									
**Triplicate 2**	2.995642	3.783994	5.505855	1.553478	1.940332	2.063936	1.956666	1.824613	3.589768									
**Triplicate 3**	3.032285	3.715876	5.372982	1.672299	2.041398	2.092912	1.999304	1.917889	3.885288									

The standard curves: $y=0.1214x+1.9$, $r^2=0.977$ (repeat 1); $y=0.0981x-2.29$, $r^2=0.9991$ (repeat 2); $y=0.1715x+0.2658$, $r^2=0.9892$ (repeat 3); y: LY concentration (μM), x: relative fluorescent intensity.

Table S3. Raw fluorometric readings from three repeats of the LY permeability experiments using triplicates.

	Control			Model			GOS 2.5 mg/ml			GOS 5 mg/ml			GOS 10 mg/ml		
	Repeat 1	Repeat 2	Repeat 3	Repeat 1	Repeat 2	Repeat 3	Repeat 1	Repeat 2	Repeat 3	Repeat 1	Repeat 2	Repeat 3	Repeat 1	Repeat 2	Repeat 3
**Triplicate 1**	0.19091	0.302814	0.263512	0.236126	0.385692	0.293685	0.234344	0.473364	0.279846	0.261249	0.373033	0.269951	0.257164	0.41859	0.263342
**Triplicate 2**	0.222291	0.273923	0.261608	0.248023	0.401791	0.291946	0.224174	0.482294	0.277826	0.243601	0.371414	0.266337	0.24906	0.39645	0.264979
**Triplicate 3**	0.195605	0.355998	0.270773	0.220899	0.38502	0.289729									

	FOS 5 mg/ml			FOS 10 mg/ml			FOS 20 mg/ml			COS 2.5 mg/ml			COS 5 mg/ml			COS 10 mg/ml		
	Repeat 1	Repeat 2	Repeat 3	Repeat 1	Repeat 2	Repeat 3	Repeat 1	Repeat 2	Repeat 3	Repeat 1	Repeat 2	Repeat 3	Repeat 1	Repeat 2	Repeat 3	Repeat 1	Repeat 2	Repeat 3
**Triplicate 1**	0.299109	0.398544	0.285948	0.236603	0.430679	0.253684	0.264908	0.494392	0.275744	0.236704	0.284011	0.254348	0.254397	0.424758	0.252756	0.353923	0.514836	0.303018
**Triplicate 2**	0.263753	0.42235	0.283351	0.203681	0.424224	0.253462	0.283813	0.474061	0.280292	0.260333	0.307194	0.242583	0.236792	0.411465	0.259391	0.348626	0.447013	0.30042
**Triplicate 3**																		

	Control			Model			Arg 1 mM			Arg 2 mM			Arg 4 mM		
	Repeat 1	Repeat 2	Repeat 3	Repeat 1	Repeat 2	Repeat 3	Repeat 1	Repeat 2	Repeat 3	Repeat 1	Repeat 2	Repeat 3	Repeat 1	Repeat 2	Repeat 3
**Triplicate 1**	0.197166	0.369674	0.26478	0.337966	0.462137	0.27956	0.224225	0.421634	0.274998	0.247216	0.358442	0.276168	0.305872	0.366852	0.279022
**Triplicate 2**	0.194175	0.35095	0.264664	0.312304	0.471744	0.276644	0.239481	0.437961	0.281662	0.24515	0.291077	0.272744	0.331217	0.383151	0.283886
**Triplicate 3**	0.214092	0.332932	0.254946	0.321589	0.430819	0.273453	0.24828	0.350722	0.273938	0.289464	0.323978	0.267899	0.35526	0.354519	0.272672

	EPA 12.5 µM			EPA 25 µM			EPA 50 µM		
	Repeat 1	Repeat 2	Repeat 3	Repeat 1	Repeat 2	Repeat 3	Repeat 1	Repeat 2	Repeat 3
**Triplicate 1**	0.318178	0.285335	0.265809	0.246563	0.363887	0.277227	0.244534	0.285972	0.262163
**Triplicate 2**	0.349469	0.3102	0.273667	0.227804	0.335475	0.275478	0.233624	0.322174	0.263761
**Triplicate 3**	0.328757	0.219717	0.272003	0.252791	0.333494	0.261047	0.212314	0.248129	0.272364

	Control			Model			RES 25 µM			RES 50 µM			RES 100 µM		
	Repeat 1	Repeat 2	Repeat 3	Repeat 1	Repeat 2	Repeat 3	Repeat 1	Repeat 2	Repeat 3	Repeat 1	Repeat 2	Repeat 3	Repeat 1	Repeat 2	Repeat 3
**Triplicate 1**	0.270063	0.315693	0.254749	0.366147	0.403963	0.3026	0.297372	0.427639	0.269831	0.245992	0.330704	0.280607	0.319579	0.380413	0.264316
**Triplicate 2**	0.272428	0.266526	0.257555	0.389232	0.454841	0.31257	0.306816	0.4458	0.269417	0.220407	0.335294	0.270595	0.289072	0.354632	0.263608
**Triplicate 3**	0.21876	0.313126	0.258571	0.40575	0.456249	0.308982	0.266223	0.453149	0.271903	0.237104	0.414184	0.272964	0.279944	0.379198	0.24767

	ALA 25 µM			ALA 50 µM			ALA 100 µM		
	Repeat 1	Repeat 2	Repeat 3	Repeat 1	Repeat 2	Repeat 3	Repeat 1	Repeat 2	Repeat 3
**Triplicate 1**	0.271186	0.368813	0.276572	0.177041	0.337913	0.305258	0.321753	0.308921	0.286896
**Triplicate 2**	0.301911	0.313058	0.276626	0.190005	0.391449	0.29836	0.330698	0.349952	0.283721
**Triplicate 3**	0.312415	0.352439	0.270056	0.211135	0.332838	0.309219	0.358486	0.318204	0.278149

The standard curves: $y=0.508x+0.0019$, $r^2=0.994$ (repeat 1); $y=0.0791x-0.0221$, $r^2=0.9881$ (repeat 2); $y=0.0121x+0.2113$, $r^2=0.991$ (repeat 3); y: MDA concentration (nM), x: OD value.

Table S4. Raw colorimetric readings from the three repeats of the MDA experiments using triplicates.
